# Electrifying Friedel–Crafts Intramolecular Alkylation toward 1,1-Disubstituted Tetrahydronaphthalenes

**DOI:** 10.1021/acs.joc.3c01281

**Published:** 2023-11-30

**Authors:** Enrico Lunghi, Pietro Ronco, Federico Della Negra, Beatrice Trucchi, Massimo Verzini, Daniele Merli, Emanuele Casali, C. Oliver Kappe, David Cantillo, Giuseppe Zanoni

**Affiliations:** † Department of Chemistry, University of Pavia, Viale Taramelli, 27100 Pavia, Italy; ‡ Flamma S.p.A., Via Bedeschi, 24040 Chignolo D’isola, Italy; § Institute of Chemistry, University of Graz, NAWI Graz, Graz 8010, Austria; ∥ Center for Continuous Flow Synthesis and Processing (CCFLOW), Research Center Pharmaceutical Engineering GmbH (RCPE), Graz 8010, Austria

## Abstract

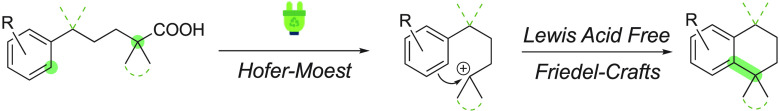

In this work, we successfully employed electrochemical conditions to promote a Hofer–Moest, intramolecular Friedel–Crafts alkylation sequence. The reaction proceeds under mild conditions, employing carboxylic acids as starting materials. Notably, the electrochemical process performed in batch was adapted to a continuous flow electrolysis apparatus to provide a significant improvement. This catalyst-free, electrochemical approach produces an array of tetrahydronaphthalenes that could be used for API synthesis.

## Introduction

The Friedel–Crafts alkylation of arenes and heteroarenes, serendipitously discovered in 1877 by Charles Friedel and James Mason Crafts, is one of the most powerful C–C bond-forming processes in organic synthesis.^1^ Thus, during the past nearly 150 years, the Friedel–Crafts reaction has been established as a fundamental reaction in chemistry, having an enormous impact on the petrochemical, pharmaceutical, dye, and agrochemical industries.^2^ The generally accepted mechanism for the Friedel–Crafts alkylation involves the intermediacy of a carbocation, whose formation is promoted by strong Lewis or Brønsted acids. Unfortunately, the employment of harsh conditions, generation of corrosive waste, and use of hazardous solvents have all raised concerns about the application of the Friedel–Crafts reaction in the modern era of organic chemistry. It is therefore not surprising that a report in 2007 from the ACS Green Chemistry Pharmaceutical Roundtable identified Friedel–Crafts reactions as a top research priority,^3^ for which the development of milder and environmentally benign alternatives would be advantageous.^4^

Electroorganic synthesis has become one of the most attractive research topics in recent years.^5^ It provides highly versatile synthetic methodologies while adhering to most principles of green chemistry.^6^ Electrochemical methods are based on the use of electrical current instead of chemical reagents to drive organic transformations and, therefore, are intrinsically clean, featuring very high atom economy. Moreover, if the electrical power is produced from renewable resources, the chemical process is even greener, significantly reducing the carbon footprint typical of the chemical industry.^7^ Electrochemical alkylation of arenes was described in 2003 by the Yoshida group using the “cation pool” method, in which an anodically generated *N*-acyliminium ion was reacted with electron-rich aromatics.^8^ More recently, Lielpetere and Jirgensons developed an electrochemical Friedel–Crafts alkylation using the trimethylstannylmethyl group as an electroauxiliary, in which tin acts as the electrochemically active moiety and leaving group, thus facilitating the generation of the carbocationic intermediate.^9^ However, the use of a toxic trimethylstannylmethyl group as an electroauxiliary poses safety and environmental concerns. An interesting alternative for the electrochemical generation of carbocations, known since 1902, is the Hofer–Moest reaction; this transformation is based on the electrolysis of carboxylic acids under mildly alkaline conditions^10^ and presents two significant advantages. (1) Carboxylic acids, and their derivatives, are highly abundant among natural and synthetic organic compounds, and (2) carbocations are generated in a non-acidic environment due to the cathodic reduction of the “proton” to hydrogen. However, to the best of our knowledge, Hofer–Moest-type generation of carbocations from aliphatic carboxylic acids coupled with intramolecular trapping with arenes has not been reported.

Herein, we report a novel, reagent-free electrochemical method for the synthesis of tetralins based on Friedel–Crafts-type decarboxylative cycloalkylation. The electrochemical procedure was transferred to a flow electrolysis cell, which provided significantly higher yields in comparison to those of standard batch electrolysis.

## Results and Discussion

A preliminary exploration of the experimental conditions was carried out on the basis of the method reported by Baran and co-workers^11^ using acid **2b** as the model substrate. Unfortunately, mixtures of decomposition products were observed in this case. In addition, these conditions required the use of stoichiometric amounts of Ag. Alternatively, conditions developed by Ley and Zhang were employed,^12^ using an undivided cell, carbon felt and a platinum plate as the anode and cathode, respectively, nBu_4_NOAc as the supporting electrolyte, and HFIP as the solvent, to obtain the corresponding Friedel–Crafts product **2**, albeit in low yield (15%). Therefore, an optimization study, including screening of several supporting electrolytes, bases, solvents, electrodes, current density, and amount of charge (F/mol), was performed (Table 1). All reactions were carried out in batch at room temperature using a commercial undivided cell [IKA ElectraSyn 2.0, 10 mL vial (see the Supporting Information for details)]. The optimal conditions for the electrochemical decarboxylation–alkylation cascade used a graphite anode and a stainless-steel cathode, a 14:1 DCM/HFIP solvent mixture, Bu_4_NPF_6_ as the supporting electrolyte, and 2,4,6-collidine (2 equiv) as an additive base. Under these conditions, a current of 7.5 mA (∼5 mA/cm^2^) provided a 65% GC yield (62% isolated) after 2.1 F/mol had been passed through the cell (entry 1).

**Table 1:**
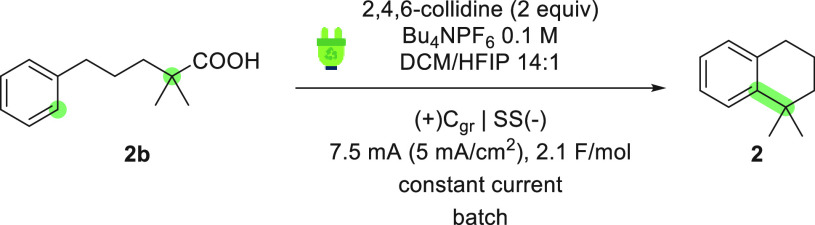
Optimization of the Reaction Conditions for the Electrochemical Decarboxylative Cycloalkylation of **2b**^a^

entry	variation from the optimal conditions	yield (%)^b^
1	none	65 (62)^c^
2	*n*Bu_4_NClO_4_	21
3	*n*Bu_4_NBF_4_	30
4	Pt anode	0
5	RVC anode	0
6	Zn cathode	60
7	graphite cathode	59
8	5 mA	51
9	10 mA	38
10	2.5 F/mol	49
11	1.5 F/mol	48
12	14:1 acetone/HFIP	0
13	14:1 MeCN/HFIP	0
14	14:1 DMSO/HFIP	0
15	2,6-lutidine	43
16	DBU	46
17	4-methoxypyridine	53
18	2,4,6-collidine (1 equiv)	40
19	2,4,6-collidine (4 equiv)	45

a Conditions: 0.2 mmol of **2b** in a 10 mL reaction mixture, 10 mL ElectraSyn 2.0 vial, constant current, 1.45 h for 0.2 mmol. C_gr_ = graphite; SS = stainless steel.

b Calibrated GC yield.

c Isolated yield.

Both the nature of the anion present in the supporting electrolyte and the electrode materials proved to be critical in achieving high yields (entries 2 and 3, respectively). When Pt or reticulated vitreous carbon (RVC) was used as the anode material, no product was observed (entry 4 or 5, respectively). This was not surprising, as these materials typically favor radical (Kolbe) pathways, and the mechanism of this reaction was expected to follow a cationic intermediate (*vide infra*). A change in the cathode material had a weaker effect on the reaction yield (entries 6 and 7). The yield of product **2** was significantly diminished by changing the current density (entries 8 and 9), amount of charge passed (entries 10 and 11), or the solvents employed in conjunction with HFIP (entries 12–14). Notably, no conversion was observed with solvents other than DCM. Any base other than 2,4,6-collidine provided only lower yields (entries 15–17). An increase or a decrease in the amount of base was also detrimental (entry 18 or 19, respectively). Despite extensive optimization attempts, no more than 75% conversion or 65% yield (entry 1) could be achieved.

**Scheme 1:**
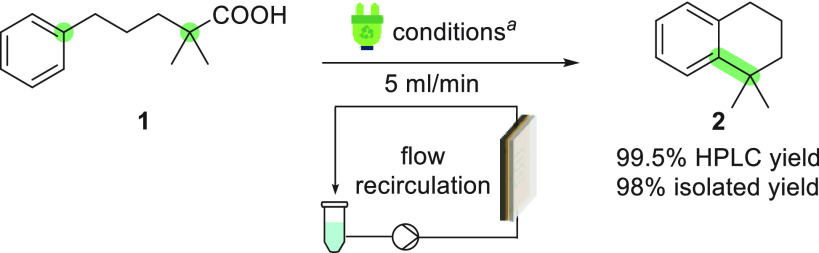
Optimized Flow Recirculation Procedure Conditions: **2b** (0.2 mmol), 2,4,6-collidine (0.4 mmol), *n*Bu_4_NPF_6_ (0.1 mmol), 14:1 DCM/HFIP (10.7 mL), undivided flow cell, 10 mA, 2.3 F/mol, (+)C_gr_|SS(−), 5 mL/min, flow recirculation mode, 3.30 h for 0.4 mmol.

In an attempt to enhance the performance of the reaction, the electrochemical procedure was transferred to a flow electrolysis cell. Flow cells exhibit a very short interelectrode distance as well as a high electrode surface area to reactor volume ratio and improved mass transfer.^13^ These features typically provide more efficient transformations. We anticipated that the enhanced mass transfer in a narrow gap electrolysis cell might have a particularly positive influence on the conversion and selectivity of the reaction. Flow experiments were carried out in a previously described cell.^14^ Initially, a recirculation approach with a flow rate of 5 mL/min was applied. Gratifyingly, after the reaction conditions had been fine-tuned in a flow electrolysis cell featuring a 0.3 mm interelectrode gap, a nearly quantitative yield of the desired product **2** was obtained (Scheme 1). Notably, this excellent yield was obtained under a relatively low current (10 mA, ∼1.6 mA/cm^2^), in contrast with the batch results, which showed that a decreased current density diminishes the yield (Table 1, entry 8).

**Scheme 2:**
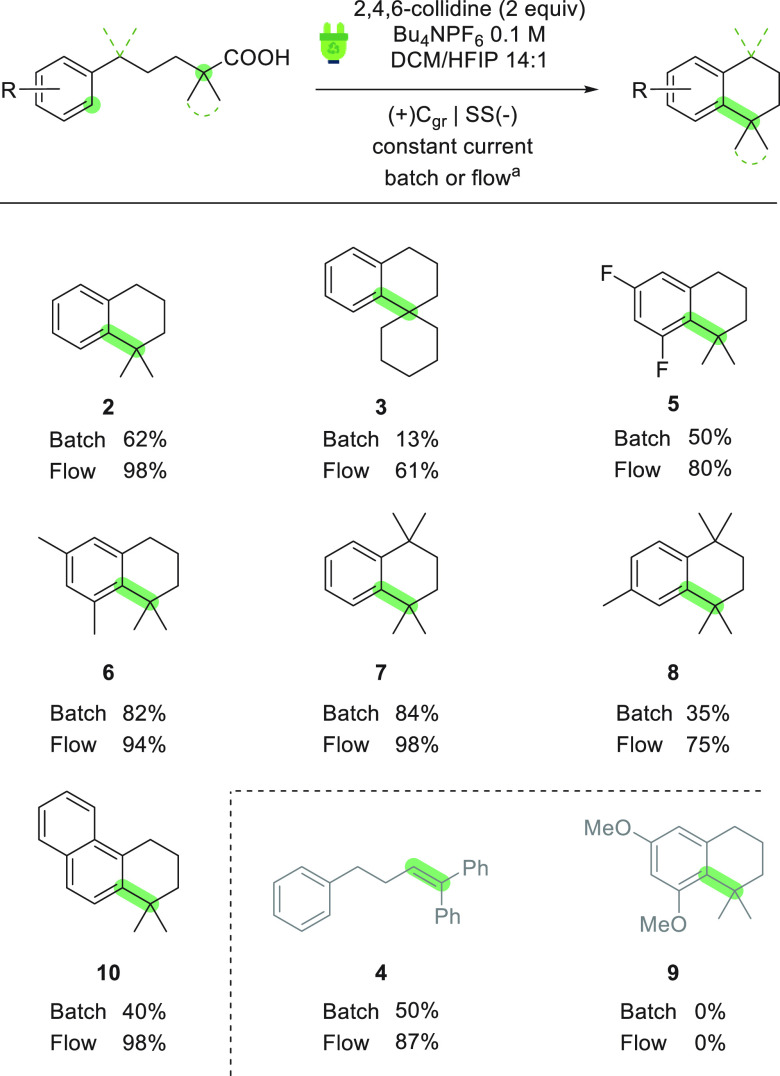
Scope of the Electrochemical Decarboxylative Cycloalkylation Conditions: **2b** (0.2 mmol), solvent (10.7 mL), constant current electrolysis. Batch: 10 mL IKA ElectraSyn 2.0 vial, 7.5 mA, 2.1 F/mol. Flow: 10 mA, 2.3 F/mol, 5 mL/min recirculation.

With the optimized reaction conditions in hand, the scope of the electrochemical decarboxylative cycloalkylation was evaluated under both batch and flow conditions (Scheme 2). Unsurprisingly, the electrochemical process performed significantly better in the flow reactor than in the batch cell in all cases. Spirocyclic compound **3**, for example, which was obtained in modest yield in a batch (13%), could be isolated in good yield (61%) in flow. The study of the reaction scope mainly focused on the introduction of methyl groups into the aliphatic chain, a characteristic feature of active ingredients containing the tetrahydronaphthalene core such as synthetic retinoids,^15^ as well as fluorine-substituted aromatics. As expected, the introduction of fluorine into the arene resulted in lower yield (**5**, 50% and 80% in batch and flow, respectively) compared to **2** due to the less nucleophilic character of the aromatic ring. On the contrary, decoration of the aliphatic chain with methyl groups did not have a significant impact on the reaction outcome (i.e., **7** and **8**). As might be expected, compound **9** could not be obtained using this methodology, under either batch or flow conditions, likely due to the low oxidation potential of the aromatic ring, which may lead to oligomerization (a cyclic voltammogram of **2b** is available in the Supporting Information).^16^

Interestingly, substrates containing two phenyl groups in the α-position to the carboxylate afforded alkene **4**. This is due to stabilization of the carbocation intermediate by the two aromatic groups, resulting in elimination of a β-proton as the preferred pathway.

**Figure 1:**
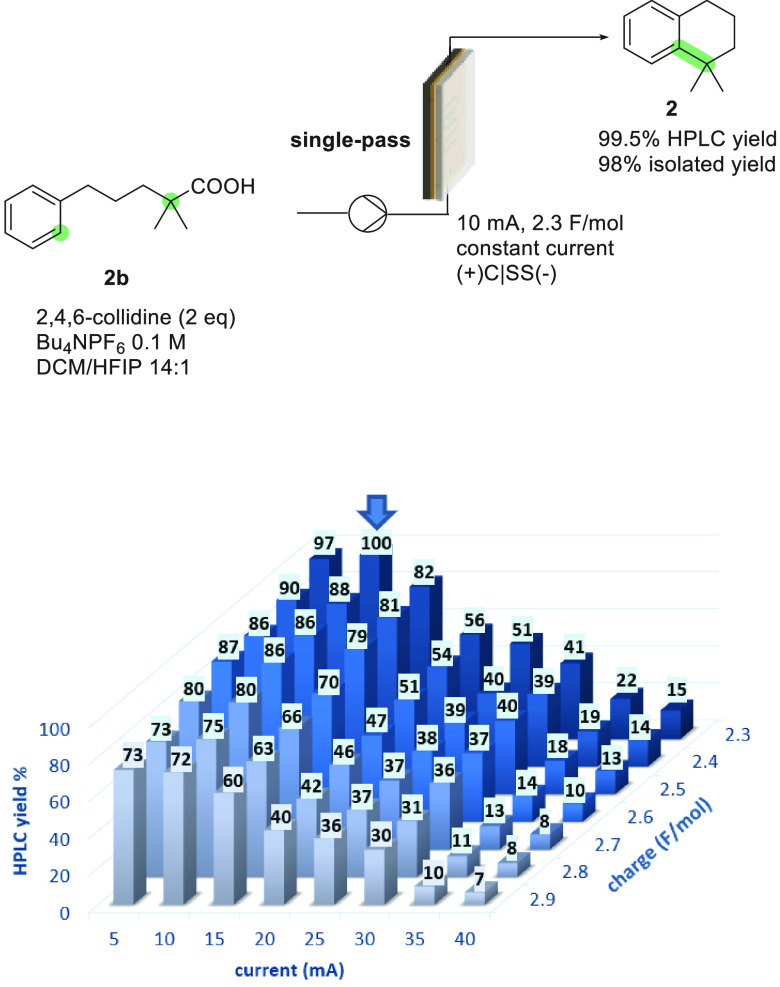
Optimization of the electrolysis parameters in a flow electrolysis cell operating in single-pass mode.

We next turned our attention to achieving a continuous, single-pass flow electrolysis protocol. Single-pass electrolysis can be very interesting from the process chemistry viewpoint, as it enables continuous generation of synthetic intermediates and products and the possibility of integrating several synthetic and purification steps into a continuous stream.^16^ Moreover, the small reactor volume resulting from the narrow gap in flow cells typically provides short residence times of the reaction mixture within the reaction zone, enabling a rapid screening of the reaction conditions. Indeed, a wide range of currents and amounts of charge could be readily screened (Figure 1). In this optimization experiment, a stock solution containing **2b**, 2,4,6-collidine (2 equiv), and *n*Bu_4_NPF_6_ (0.1 M) in a 14:1 DCM/HFIP mixture was continuously pumped through the electrolysis cell.

The electrolysis parameters (cell current and pump flow rate, which determines the amount of charge) were successively adjusted, and after steady state conditions had been reached, aliquots of the crude mixture obtained from the reactor output were analyzed by HPLC (Figure 1; see Table S12 for additional data). As expected from the results in recirculation mode, lower current densities provided the best results. Notably, a quantitative yield was observed under electrolysis parameters (10 mA, 2.3 F/mol) analogous to those used in recirculation mode (cf. Scheme 1), also implying that both recirculation and single-pass operation presented the same current efficiency.

Another relevant parameter in single-pass flow electrochemical processing is the reactor stability, i.e., the performance of the electrolysis cell over prolonged reaction times. In some cases, both electrode fouling and corrosion may affect reaction performance over time, preventing the obtention of the target amounts and quality of material in a continuous manner. Thus, under the optimal reaction conditions (Figure 1, 10 mA, 0.108 mL/min, and 2.3 F/mol), the single-pass continuous process was carried out without interruption for 24 h. Gratifyingly, monitoring the crude reaction mixture obtained from the reactor output by HPLC confirmed that a quantitative yield (>98%) had been obtained over the entire period (see Figure S60); workup of the reaction produced 980 mg (7.2 mmol, 98%) of the pure material. Notably, the flow electrolysis experiments performed very well and were very analogous in both operation modes. In both cases, 10 mA of current and 2.3 F/mol of charge were applied, which means that the cell productivity was equal in both cases.

**Figure 2:**
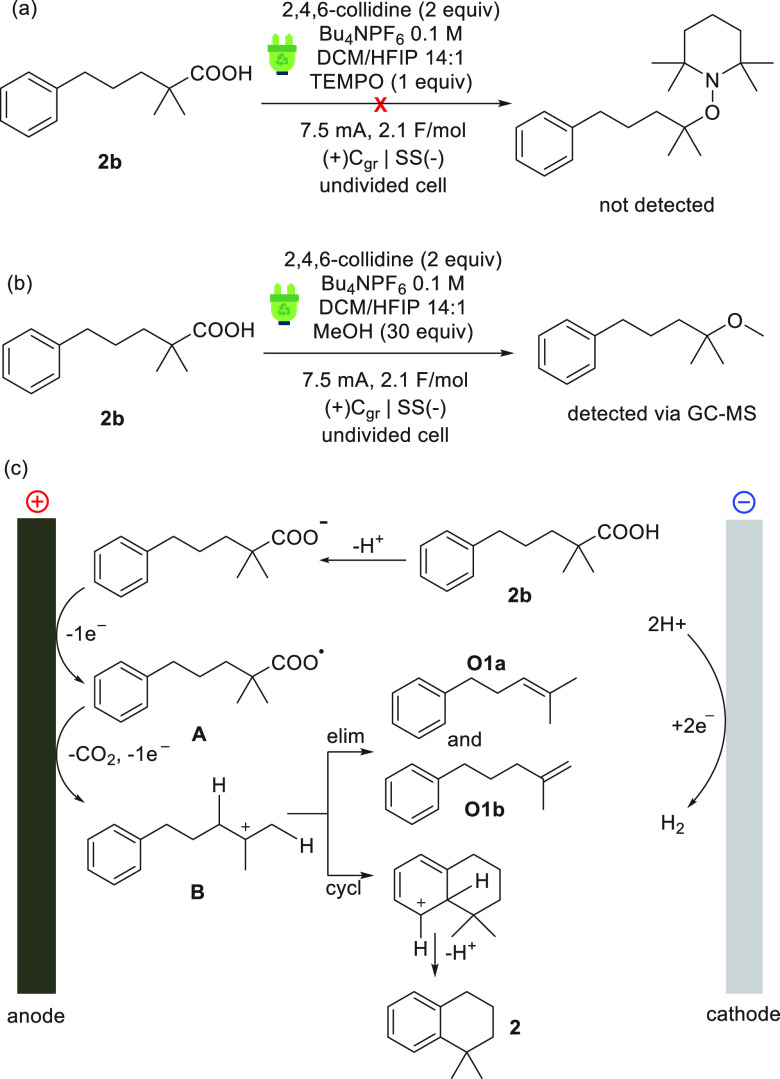
(a) Radical trapping experiment, (b) carbocation trapping experiment, and (c) suggested mechanism for the electrochemical decarboxylative cycloalkylation.

On the basis of the literature on the Hofer–Moest reaction,^11,17^ radical trapping experiments, and density functional theory (DFT) calculations, a plausible mechanistic scenario for the electrochemical transformation was proposed (Figure 2). First, an experiment under the optimal conditions was carried out using radical trapping agent TEMPO as an additive and provided no traces of the substrate–TEMPO adduct (Figure 2a), supporting the idea that the reaction does not follow a radical mechanism. To probe for the formation of a carbocation, the employment of MeOH as an additive (Figure 2b) led to the corresponding methyl ether, an intermediate observed by GC-MS.

On the basis of this mechanistic evidence, the electrochemical reaction likely commences with the deprotonation of carboxylic acid in the presence of 2,4,6-collidine. Anodic oxidation of the corresponding carboxylate generates radical intermediate **A**, which undergoes decarboxylation followed by a second anodic oxidation to form carbocation **B**. In the absence of other nucleophiles, the aromatic system traps the carbocation in a cycloalkylation, forming product **2** by deprotonation. Intermediate **B** can also eliminate a β-proton, giving elimination side product **O1a** or **O1b**. The cathodic reduction at the counter electrode is the reduction of two protons to hydrogen, thus keeping the pH of the solution constant.

**Figure 3:**
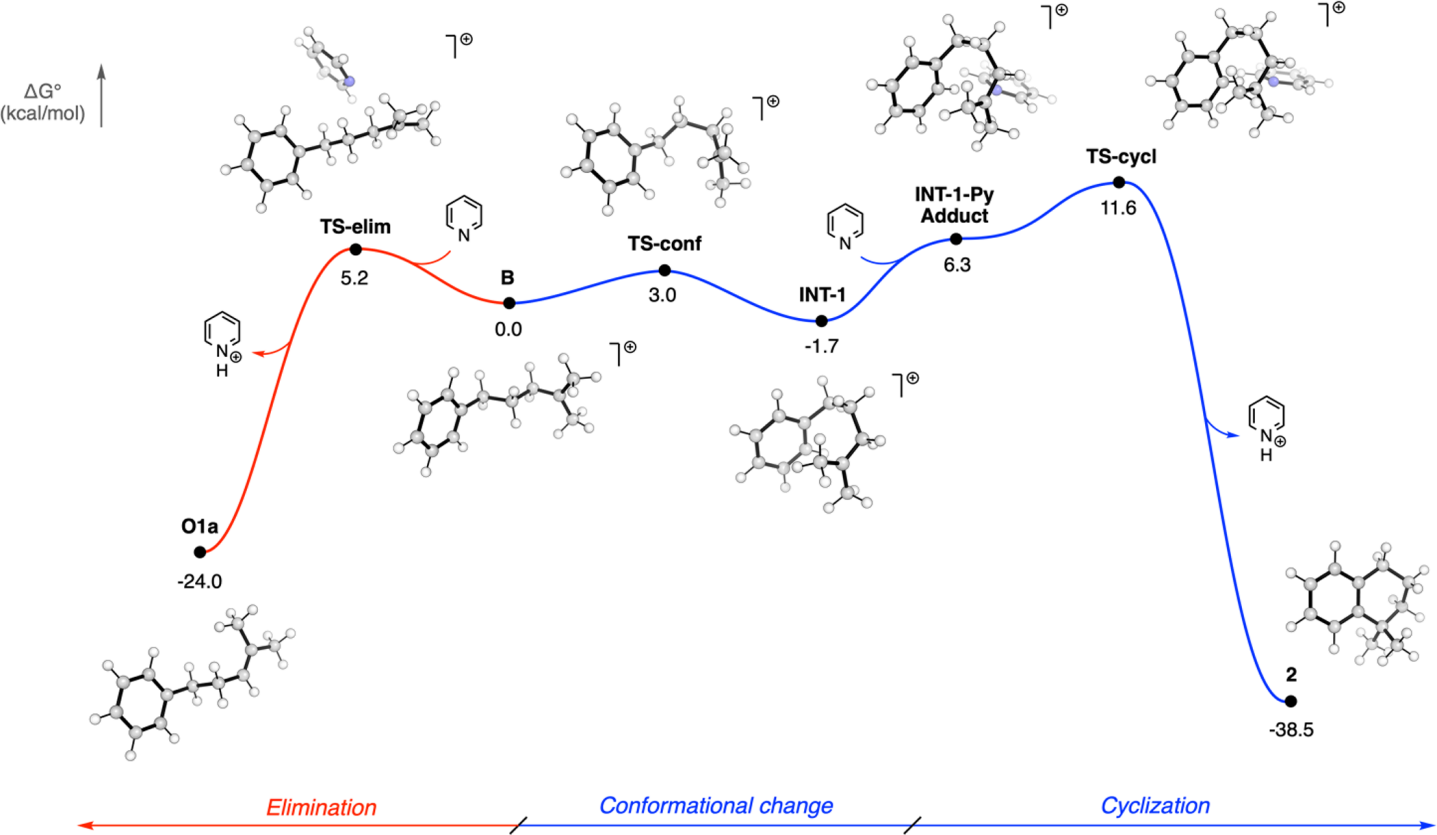
Electronic free energy profile (kilocalories per mole) for both the cyclization and the elimination reactions [SMD(DCM)-wB97XD/def2TZVP//SMD(DCM)-M06-2x/6-31+g(d,p)].

By looking closely at the mechanism we proposed above, we can clearly see that two side products were observed together with the cyclized one: namely, terminal bis-substituted olefin **O1b** and tris-substituted **O1a**. To further support the mechanistic hypothesis and generatation of elimination products, DFT calculations were performed. The mechanisms for both the cyclization and the elimination reaction steps were investigated using Gaussian 16 in the framework of DFT.^18^ The cationic substrate was modeled without any simplifications to remain as close as possible to the real condition, while the role of the non-oxidizable base 2,4,6-collidine was played by pyridine. To better sample the conformational space for the starting cationic substrate, we employed the semiempirical extended tight binding [*x*TB (*GFN2-xTB*)] approach (see the Supporting Information for more details).^19^ We next moved to the proper choice of functional and basis set for the DFT calculation. Soon we encountered problems in simulating the elimination reaction step. Different combinations of functional and basis sets were tested, but most of them proved to be unsuccessful (see the Supporting Information for a full description of the computational benchmark). Given the reasonable existence of the elimination transition state, we used the M06-2X hybrid functional of Truhlar and Zhao, which includes double the amount of nonlocal exchange (2X).^20^ This functional proved to be one of the first level choices for thermochemistry and thermochemical kinetics studies, by paying particular attention to noncovalent interactions given the presence of medium-range correlation contributions and a flexible functional form with extensive parametrization.^20^ We next selected for each atom the same basis set, 6-31+g(d,p).^21^ To reproduce the effect of dichloromethane as a solvent, we included Truhlar’s solute electron density and a continuum solvation model (SMD).^22^ For each structure, we performed frequency calculations to confirm the effective minimum or transition state (TS) nature of the optimized system. Moreover, intrinsic reaction coordinate (IRC) calculations were performed to confirm the continuity of the reaction profile from TSs toward reactants, intermediates, or products.^23^ Finally, single-point (SP) calculations were performed using the optimized structures using the long-range corrected hybrid density functional ωB97XD from Head-Gordon and co-workers, which includes a version of Grimme’s D2 dispersion model,^24^ and def2-TZVP, a valence triple-ζ polarization basis set from Ahlrichs and co-workers.^25^ During the SP calculation, we maintained the same SMD model for the dichloromethane solvent. The final reported energies were then thermally corrected to the free energy. All of the subsequent evaluations will be referred to this level of theory. Figure 3 shows the complete energetic profile of the two possible mechanistic pathways.

Our analysis begins from the elimination step, colored red in Figure 3. Starting from initial cation **B**, pyridine can either directly deprotonate at the terminal position, thus forming the disubstituted olefin, or deprotonate the more hindered internal position to form the trisubstituted alkene. Despite attempts to locate the transition state (TS) corresponding to terminal deprotonation, we were able to locate and optimize only the TS leading to a more substituted internal olefin (**TS-elim**). This step requires only 5.2 kcal/mol from the isolated reactants and appears to be thermodynamically favored because final eliminated product **O1a** is 24.0 kcal/mol more stable than the initial **B**. With regard to this step, we strongly believe the most statistically probable terminal methyl groups are deprotonated, which is followed by a fast pyridine/pyridinium-promoted “push-and-pull” equilibration that populates the most substituted internal olefin. We attempted to also simulate this equilibration but were not succeessful in locating the isomerization transition state.

With regard to the cyclization step (Figure 3, blue line), we observed that a conformational modification has to occur to produce an intermediate **INT-1** capable of promoting the cyclization. This conformational step occurs through **TS-conf** with an activation energy of 3.0 kcal/mol and shows a modification of the dihedral angle between the benzylic and homobenzylic carbons from 178.5° to 130.9° (in **TS-conf**) and finally to 60.3° in **INT-1**. This change in dihedral length significantly shortens the distance between the tertiary carbon (where the cation charge is stabilized) and the carbon of the benzene ring where the cyclization occurs (from 5.62 Å in **B** to 2.87 Å in **INT-1**). Starting from **INT-1**, the introduction of pyridine forms reactive adduct **INT-1-Py**, which is then prone to promoting the cyclization step. This occurs through **TS-cyc** with an activation energy of 5.3 kcal/mol from **INT-1-Py** and 13.3 kcal/mol from conformational productive intermediate **INT-1**. During cyclization, the distance between the reactive carbons is reduced from 2.87 Å in **INT-1** to 2.26 Å in **TS-cyc** and finally to 1.53 Å in **2**. The removal of the proton from the benzene ring to re-establish aromaticity is mediated by pyridine. The rearomatization step occurs concomitantly with cyclization but shows a strong asynchrony given the fact that first the cyclization occurs and only in a second time does the rearomatization occur. This observation is corroborated by the distance between the pyridine nitrogen and the proton to be removed. In the **INT-1-Py** adduct, this distance is 2.49 Å, which is reduced to 2.31 Å in **TS-cyc**. Only after the formation of the cycle does the distance decrease until the formation of a N–H bond in the pyridinium cation (1.02 Å). This step is thermodynamically favored because final cyclized product **2** is 38.5 kcal/mol more stable than initial product **B**.

To engage in a general discussion of the reaction profile we reported above, we can state that the reaction seems to be under Curtin–Hammett control. Indeed, the formation of eliminated product **O1a** is less energetically demanding because it needs only 5.2 kcal/mol to reach **TS-elim**, but the less stable product (i.e., olefin **O1a**) is obtained. We can consider the elimination step as the kinetic one. On the contrary, the cyclization step (**TS-cycl**) requires 13.3 kcal/mol, nearly double the activation energy needed to overcome the elimination transition state (**TS-elim**), but cyclized product **2** is much more stable than the eliminated one (**O1a**) (ΔΔ*G*° ≈ 14.0 kcal/mol). This means that the reaction equilibrates between the eliminated product, which is formed first, and the cyclized product, which is much more energetically costly but remains the most thermodynamically favored.

## Conclusions

In summary, we have developed an electrochemical methodology for the synthesis of substituted tetrahydronaphthalenes via the oxidative decarboxylative cycloalkylation of carboxylic acids. This electrochemical procedure, based on direct anodic oxidation, avoids the use of the corrosive reagents typically needed in Friedel–Crafts-type protocols and uses carboxylic acids as readily available substrates. A significant enhancement of the reaction yield was achieved when the electrochemical procedure was performed in a flow electrolysis cell, which can be operated in both recirculation and single-pass modes. Very good to quantitative yields were obtained for tetrahydronaphthalenes decorated with fluorine and methyl groups.

## Data Availability

The data underlying this study are available in the published article and its Supporting Information.

## Supporting Information Available

Experimental procedures, evaluation of the conditions, characterization data, NMR spectra, optimization details, and computational coordinates (PDF)

## References

[ref1] aFriedel C.; Friedel J. M. The alkylation or acylation of aromatic compounds catalysed by aluminium chloride or other Lewis acids. Compt. Rend. 1877, 84, 1392–1395.

[ref2] aOlah G. A.; Reddy V. P.; Prakash G. K. S. Friedel-Crafts Reactions. In Encyclopedia of Chemical Technology; Wiley, 2000.

[ref3] Constable D. J. C.; Dunn P. J.; Hayler J. D.; Humphrey G. R.; Leazer J. L. Jr.; Linderman R. J.; Lorenz K.; Manley J.; Pearlman B. A.; Wells A.; Zaks A.; Zhang T. Y. Key green chemistry research areas—a perspective from pharmaceutical manufacturers. Green Chem. 2007, 9, 411–420. 10.1039/B703488C.

[ref4] aRueping M.; Nachtsheim B. J. A review of new developments in the Friedel–Crafts alkylation – From green chemistry to asymmetric catalysis. Beilstein J. Org. Chem. 2010, 6, 6 and references cited therein 10.3762/bjoc.6.6. 20485588 PMC2870981

[ref5] aYan M.; Kawamata Y.; Baran P. S. Synthetic Organic Electrochemical Methods Since 2000: On the Verge of a Renaissance. Chem. Rev. 2017, 117, 13230–13319. 10.1021/acs.chemrev.7b00397. 28991454 PMC5786875

[ref6] aZhu C.; Ang N. W. J.; Meyer T. H.; Qiu Y.; Ackermann L. Organic Electrochemistry: Molecular Syntheses with Potential. ACS Cent. Sci. 2021, 7, 415–431. 10.1021/acscentsci.0c01532. 33791425 PMC8006177

[ref7] aService R. F. Renewable bonds. Science 2019, 365, 1236–1239. 10.1126/science.365.6459.1236. 31604222

[ref8] aSuga S.; Nagaki A.; Yoshida J. Highly selective Friedel–Crafts monoalkylation using micromixing. Chem. Commun. 2003, 354–355. 10.1039/b211433j. 12613609

[ref9] aLielpetere A.; Jirgensons A. Friedel–Crafts Alkylation with Carbenium Ions Generated by Electrochemical Oxidation of Stannylmethyl Ethers. Eur. J. Org. Chem. 2020, 2020, 4510–4516. 10.1002/ejoc.202000568.

[ref10] aHofer H.; Moest M. Mittheilung aus dem elektrochemischen Laboratorium der Königl, Technischen Hochschule zu München. Justus Liebigs Ann. Chem. 1902, 323, 284–323. 10.1002/jlac.19023230304.

[ref11] Xiang J.; Shang M.; Kawamata Y.; Lundberg H.; Reisberg S. H.; Chen M.; Mykhailiuk P.; Beutner G.; Collins M. R.; Davies A.; Del Bel M.; Gallego G. M.; Spangler J. E.; Starr J.; Yang S.; Blackmond D. G.; Baran P. S. Hindered dialkyl ether synthesis with electrogenerated carbocations. Nature 2019, 573, 398–402. 10.1038/s41586-019-1539-y. 31501569 PMC6996793

[ref12] Bu F.; Lu L.; Hu X.; Wang S.; Zhang H.; Lei A. Electrochemical oxidative decarboxylation and 1,2-aryl migration towards the synthesis of 1,2-diaryl ethers. Chem. Sci. 2020, 11, 10000–10004. 10.1039/D0SC03708G. 34094264 PMC8162141

[ref13] aAtobe M.; Tateno H.; Matsumura Y. Applications of Flow Microreactors in Electrosynthetic Processes. Chem. Rev. 2018, 118, 4541–4572. 10.1021/acs.chemrev.7b00353. 28885826

[ref14] Jud W.; Kappe C. O.; Cantillo D. Development and Assembly of a Flow Cell for Single-Pass Continuous Electroorganic Synthesis Using Laser-Cut Components. Chem. Methods 2021, 1, 36–41. 10.1002/cmtd.202000042.

[ref15] Kagechika H.; Shudo K. Synthetic Retinoids: Recent Developments Concerning Structure and Clinical Utility. J. Med. Chem. 2005, 48, 5875–5883. 10.1021/jm0581821. 16161990

[ref16] aTanbouza N.; Ollevier T.; Lam K. Bridging Lab and Industry with Flow Electrochemistry. iScience 2020, 23, 101720 10.1016/j.isci.2020.101720. 33205027 PMC7653055

[ref17] aHintz H. A.; Sevov C. S. Catalyst-controlled functionalization of carboxylic acids by electrooxidation of self-assembled carboxyl monolayers. Nat. Commun. 2022, 13, 1319 10.1038/s41467-022-28992-4. 35288543 PMC8921278

[ref18] Frisch M. J.; Trucks G.-W.; Schlegel H. B.; Scuseria G. E.; Robb M. A.; Cheeseman J. R.; Scalmani G.; Barone V.; Petersson G. A.; Nakatsuji H.; Li X.; Caricato M.; Marenich A. V.; Bloino J.; Janesko B. G.; Gomperts R.; Mennucci B.; Hratchian H. P.; Ortiz J. V.; Izmaylov A. F.; Sonnenberg J. L.; Williams-Young D.; Ding F.; Lipparini F.; Egidi F.; Goings J.; Peng B.; Petrone A.; Henderson T.; Ranasinghe D.; Zakrzewski V. G.; Gao J.; Rega N.; Zheng G.; Liang W.; Hada M.; Ehara M.; Toyota K.; Fukuda R.; Hasegawa J.; Ishida M.; Nakajima T.; Honda Y.; Kitao O.; Nakai H.; Vreven T.; Throssell K.; Montgomery J. A. Jr.; Peralta J. E.; Ogliaro F.; Bearpark M. J.; Heyd J. J.; Brothers E. N.; Kudin K. N.; Staroverov V. N.; Keith T. A.; Kobayashi R.; Normand J.; Raghavachari K.; Rendell A. P.; Burant J. C.; Iyengar S. S.; Tomasi J.; Cossi M.; Millam J. M.; Klene M.; Adamo C.; Cammi R.; Ochterski J. W.; Martin R. L.; Morokuma K.; Farkas O.; Foresman J. B.; Fox D. J. Gaussian16, rev. C.01; Gaussian, Inc.: Wallingford, CT, 2019.

[ref19] aGrimme S.; Bannwarth C.; Dohm S.; Hansen A.; Pisarek J.; Pracht P.; Seibert J.; Neese F. Fully Automated Quantum-Chemistry-Based Computation of Spin–Spin-Coupled Nuclear Magnetic Resonance Spectra. Angew. Chem., Int. Ed. 2017, 56, 14763–14769. 10.1002/anie.201708266. PMC5698732 28906074

[ref20] Zhao Y.; Truhlar D. G. The M06 suite of density functionals for main group thermochemistry, thermochemical kinetics, noncovalent interactions, excited states, and transition elements: two new functionals and systematic testing of four M06-class functionals and 12 other functionals. Theor. Chem. Acc. 2008, 120, 215–241. 10.1007/s00214-007-0310-x.

[ref21] aClark T.; Chandrasekhar J.; Spitznagel G. W.; Schleyer P. V. R. Efficient diffuse function-augmented basis sets for anion calculations. III. The 3-21+G basis set for first-row elements, Li-F. J. Comput. Chem. 1983, 4, 294–301. 10.1002/jcc.540040303.

[ref22] Marenich A. V.; Cramer C. J.; Truhlar D. G. Universal Solvation Model Based on Solute Electron Density and on a Continuum Model of the Solvent Defined by the Bulk Dielectric Constant and Atomic Surface Tensions. J. Phys. Chem. B 2009, 113, 6378–6396. 10.1021/jp810292n. 19366259

[ref23] aGonzalez C.; Schlegel H. B. Reaction path following in mass-weighted internal coordinates. J. Chem. Phys. 1990, 94, 5523–5527. 10.1021/j100377a021.

[ref24] Chai J.-D.; Head-Gordon M. Long-range corrected hybrid density functionals with damped atom–atom dispersion corrections. Phys. Chem. Chem. Phys. 2008, 10, 6615–6620. 10.1039/b810189b. 18989472

[ref25] aWeigend F.; Ahlrichs R. Balanced basis sets of split valence, triple zeta valence and quadruple zeta valence quality for H to Rn: Design and assessment of accuracy. Phys. Chem. Chem. Phys. 2005, 7, 3297–3305. 10.1039/b508541a. 16240044

