# Supplementary material for: Electrifying Friedel–Crafts Intramolecular Alkylation toward 1,1-Disubstituted Tetrahydronaphthalenes
Source: J Org Chem. 2023 Nov 30;88(24):16783–9. doi: 10.1021/acs.joc.3c01281 (PMC10729024; doi:10.1021/acs.joc.3c01281)
Supplement: Supplementary file 1 — jo3c01281_si_001.pdf [file jo3c01281_si_001.pdf]

SUPPORTING INFORMATION

Electrifying Friedel-Crafts Intramolecular Alkylation Towards 1,1-disubstituted tetrahydronaphthalenes

Enrico Lunghi,[a] Pietro Ronco,[a] Federico Della Negra,[b] Beatrice Trucchi,[b] Massimo Verzini,[b] Daniele Merli,[a] Emanuele Casali,[a] C. Oliver Kappe,[c,d] David Cantillo,[c,d]* Giuseppe Zanoni[a]*

[a] Department of Chemistry, University of Pavia, Viale Taramelli, Pavia, Italy; Email: gz@unipv.it

[b] Flamma S.p.A, Via Bedeschi, Chignolo D'isola, Italy

[c] Institute of Chemistry, University of Graz, NAWI Graz, Graz 8010, Austria, Email: david.cantillo@uni-graz.at

[d] Center for Continuous Flow Synthesis and Processing (CCFLOW), Research Center Pharmaceutical Engineering GmbH (RCPE), Graz 8010, Austria

Table of contents

General metohods S2

Abbreviations S2

Synthesis of Starting Materials **2-10**(**b** or **e** or **g**) S4

Optimization studies on **2b** S33

Batch electrolysis of **2-10**(**b** or **e** or **g**) S48

Flow experiments S54

Flow recirculation optimization on **2b** S55

Flow recirculation electrolysis of **2-10**(**b** or **e** or **g**) S58

Flow single pass optimization on **2b** S63

Flow single pass long run electrolysis of **2b** S65

Mechanistic studies S67

Computational details S69

^{1}H and ^{13}C NMR spectra S71

Cartesian coordinates S100

References S105

General Methods

NMR spectra were recorded on Bruker-400 MHz or Bruker-300 MHz spectrometer. The high resolution mass spectra were recorded on a Thermo LTQOrbitrap XL (ESI-). GC-MS analysis were performed on “Thermo scientific” Focus GC-DSQ II. Column chromatography was performed on silica gel Sigma-Aldrich High-purity grade (9385), pore size 60˚A (230-400 mesh). TLC was performed on GF-254 Merck (0.25 mm) per TLC Sigma-Aldrich. Electrolysis experiments have been performed on IKA Electrasyn 2.0. Flow electrochemical experiments have been performed using a peristaltic pump Vapourtech SF-10 and a self-assembled flow cell. All chemicals were used without purification as commercially available unless otherwise noted.

Abbreviations:

BuLi: *n*-butyl lithium

TPPA: tris(N,N-tetramethylene) triamide phosphoric acid

i-Pr2NH: diisopropylamine

TEA: triethylamine

DIPEA: diisopropylethylamine

DBU: diazabicycloundecene

4-MeO-py: 4-methoxypyridine

KOH: potassium hydroxide

LiOH: lithium hydroxide

LDA: lithium diisopropylamide

HFIP: 1,1,1,3,3,3-Hexafluoro-2-propanol

DMSO: dimethylsulfoxide

Et_2O: diethylether

EtOH: ethanol

MeCN: acetonitrile

THF: tetrahydrofuran

DCM: dichloromethane

DMF: dimethylformamide

Bu_4NBF_4: tetrabutylammonium tetrafluoroborate

Bu_4NPF_6: tetrabutylammonium hexafluorophosphate

Bu_4NOAc: tetrabutylammonium acetate

Bu_4NOH: tetrabutylammonium hydroxide

Bu_4NClO_4: tetrabutylammonium perchlorate

C: graphite

GC: glassy carbon

RVC: reticulated vitreous carbon

Pt: platinum

SS: stainless steel

Ni: nickel

Zn: zinc

TLC: thin layer chromatography

Synthesis of starting materials 2-10(b or e or g)

Synthesis of **2b**-*Step 1*

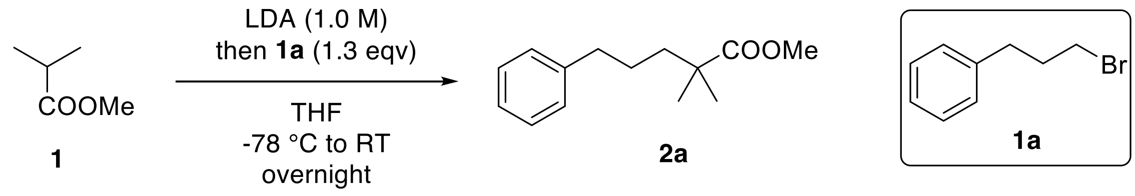

Figure S 1: Synthesis of methyl 2,2-dimethyl-5-phenylpentanoate

1.1 eq. of *n*-BuLi (55 mmol, 35 ml 1,6 M in hexanes) was added to a solution of freshly distilled diisopropylamine (1 eq, 50 mmol, 3.63 ml) in anhydrous THF (50 ml, 1.0 M) under nitrogen at 0 °C. The reaction mixture was then cooled to -78 °C and methyl isobutyrate **1** (0.9 eq, 45 mmol, 5.16 ml) was carefully added dropwise. The reaction was stirred at the same temperature for 30 min. and then bromide **1a** (1 eq, 50 mmol, 13.7 ml) was added dropwise. The mixture is stirred overnight slowly reaching RT.

The reaction was quenched adding one volume of a saturated solution of ammonium chloride in water. The organic phase was separated and the aqueous phase was extracted with ethyl acetate three times. Organic phases were reunited and washed 2 times with HCl 2 M and one time with brine. The organic phase was collected and dried over sodium sulphate then the solvent was evaporated under vacuum.

The crude is a dark yellow oil and was used without purification for the next step.

MW: 220.14 g/mol

Synthesis of **2b**-*Step 2*

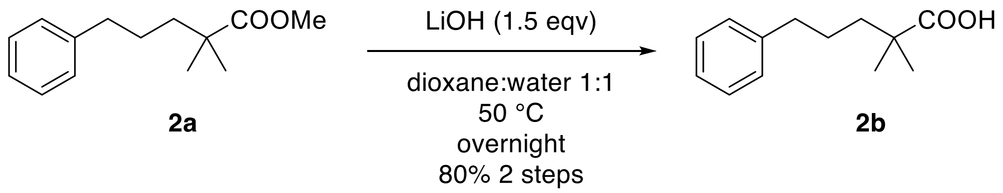

Figure S 2: Synthesis of 2,2-dimethyl-5-phenylpentanoic acid

The crude obtained from step 1 was dissolved in a 1:1 mixture water:1,4 dioxane (500 ml, 0.1 M). LiOH (1.5 eq, 2.0 g) was added to the reaction mixture and the temperature was brought to 50 °C using an oil bath under magnetic stirring for one night.

After the mixture was cooled to RT, 1,4 dioxane was removed under vacuum. The residue was washed three times with diethyl ether. Aqueous phase was then acidified with HCl 1 M (aq) until the pH was 3, then the same phase was extracted three times with ethyl acetate. The organic phase collected after the extraction of the acid phase was dried over sodium sulphate and the solvent was removed under vacuum.

The product **2b** results as a white solid and no further purification was required. Y% = 80% (8.2 g) over two steps.

MW: 206.28 g/mol

^{1}H NMR (300 MHz, Chloroform-*d*) δ 7.36 – 7.25 (m, 2H), 7.25 – 7.15 (m, 3H), 2.71 – 2.55 (m, 2H), 1.69 – 1.58 (m, 3H), 1.21 (s, 6H).
^{13}C{^{1}H} (101 MHz, Chloroform-*d*) δ 185.3, 142.2, 128.5, 128.4, 125.9, 42.2, 40.2, 36.4, 26.8, 25.0.

HRMS (ESI-): m/z [M-H]$^-$, calculated for $C_{13}H_{17}O_2^-$: 205.1234 ; found: 205.1225.

Synthesis of **3b**-*Step 1*

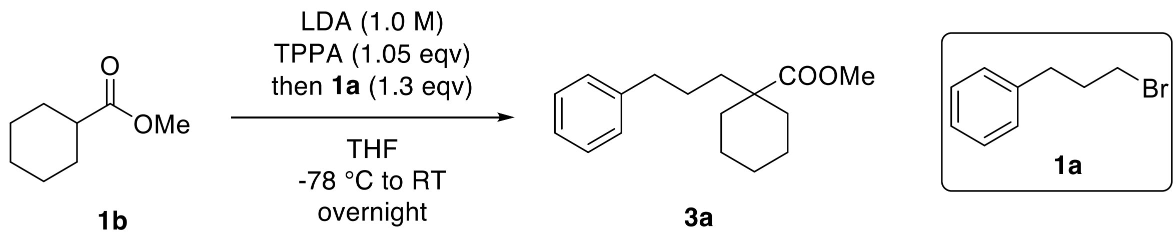

Figure S 3: methyl 1-(3-phenylpropyl)cyclohexane-1-carboxylate

1.1 eq. of *n*-BuLi (1 mmol, 625 µl 1,6 M in hexanes) was added to a solution of freshly distilled diisopropylamine (1 eq, 1 mmol, 141 µl) in anhydrous THF (1 ml, 1.0 M) under nitrogen at 0 °C. The reaction mixture was then cooled to -78 °C and a solution of ester **1b** (0.9 eq, 0.9 mmol, 143.8 µl) and TPPA (1.05 eq, 1.05 mmol, 261 µl) in dry THF (1 ml, 1.1 M) was carefully added dropwise. The reaction was stirred at the same temperature for 30 min. and then bromide **1a** (1 eq, 50 mmol, 91.7 µl) was added dropwise. The mixture is stirred overnight slowly reaching RT.

The reaction was quenched adding one volume of a saturated solution of ammonium chloride in water. The organic phase was separated and the aqueous phase was extracted with ethyl acetate three times. Organic phases were reunited and washed 2 times with HCl 2 M and one time with brine. The organic phase was collected and dried over sodium sulphate then the solvent was evaporated under vacuum.

The crude was purified on silica gel using 98:2 Hexane/EtOAc as mobile phase. The resulting yellow oil was used for the step 2. Y% : 77% (200 mg). The ^{1}H NMR analysis was correspondent to the one reported by Cala, Lara et al. (1)

MW: 260.37 g/mol

Synthesis of **3b**-*Step 2*

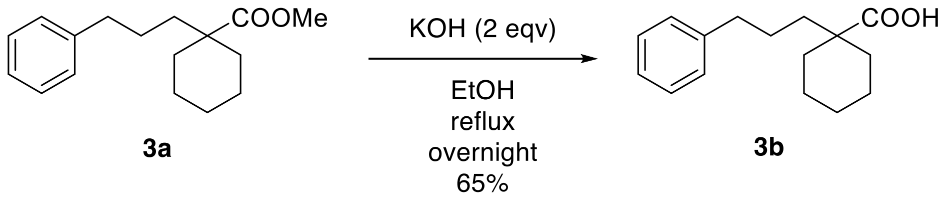

Figure S 4: Synthesis of 1-(3-phenylpropyl)cyclohexane-1-carboxylic acid

The ester **3a** (1 eq, 200 mg) was dissolved in ethanol (10 ml, 0.1 M). KOH (2 eq, 27.5 mg) was slowly added and the reaction was brought to reflux overnight using an oil bath under magnetic stirring.

The mixture was cooled to RT and the solvent was evaporated under vacuum. The residue was dissolved in water and the aqueous phase was extracted three times with diethyl ether. Aqueous phase was then acidified with HCl 1 M until the pH was 3, then the same phase was extracted three times with ethyl acetate. The organic phase collected after the extraction of the acid phase was dried over sodium sulphate and the solvent was removed under vacuum.

The product **3b** was obtained as an orange solid. No further purification was needed. Y% = 98% (185.7 mg)

MW: 246.35 g/mol

^{1}H NMR (400 MHz, Chloroform-*d*) δ 7.34 – 7.27 (m, 2H), 7.25 – 7.18 (m, 3H), 2.62 (t, *J* = 6.9 Hz, 2H), 2.17 – 2.03 (m, 2H), 1.73 – 1.53 (m, 7H), 1.51 – 1.38 (m, 2H), 1.34 – 1.19 (m, 3H).
^{13}C{^{1}H} NMR (101 MHz, Chloroform-*d*) δ 183.8, 142.3, 128.5, 128.4, 125.9, 46.9, 36.3, 33.9, 26.0, 25.8, 23.3.

HRMS (ESI-): m/z $[M-H]^-$, calculated for $C_{16}H_{21}O_2^-$: 245.1547; found: 245.1544.

Synthesis of **4b**-*Step 1*

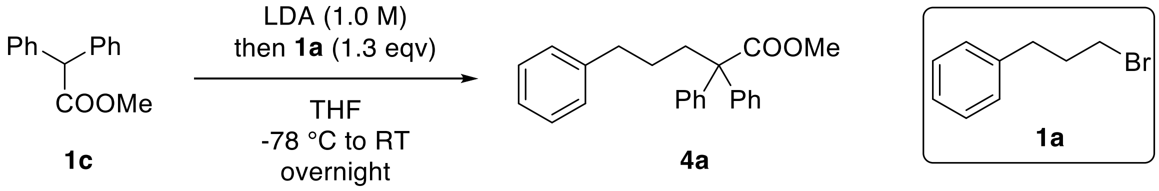

Figure S 5: Synthesis of methyl 2,2,5-triphenylpentanoate

1.1 eq. of *n*-BuLi (1 mmol, 625 µl 1,6 M in hexanes) was added to a solution of freshly distilled diisopropylamine (1 eq, 1 mmol, 141 µl) in anhydrous THF (1 ml, 1.0 M) under nitrogen at 0 °C. The reaction mixture was then cooled to -78 °C and ester **1c** (0.9 eq, 0.9 mmol, 228 µl) was carefully added dropwise. The reaction was stirred at the same temperature for 30 min. and then bromide **1a** (1 eq, 50 mmol, 91.7 µl) was added dropwise. The mixture is stirred overnight slowly reaching RT.

The reaction was quenched adding one volume of a saturated solution of ammonium chloride in water. The organic phase was separated and the aqueous phase was extracted with ethyl acetate three times. Organic phases were reunited and washed 2 times with HCl 2 M and one time with brine. The organic phase was collected and dried over sodium sulphate then the solvent was evaporated under vacuum.

The crude residue was used directly for the step 2 without further purification.

MW: 344.45 g/mol

Synthesis of **4b**-*Step 2*

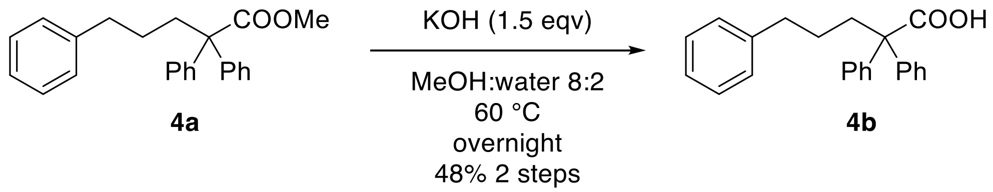

Figure S 6: Synthesis of 2,2,5-triphenylpentanoic acid

The crude ester **4a** (400 mg) was dissolved in methanol:water 8:2 (10 ml, 0.1 M). KOH (2 eq, 27.5 mg) was slowly added and the reaction was brought to reflux overnight using an oil bath under magnetic stirring.

The mixture was cooled to RT and the methanol was evaporated under vacuum. The residue was dissolved in water and the aqueous phase was extracted three times with diethyl ether. Aqueous phase was then acidified with HCl 1 M until the pH was 3, then the same phase was extracted three times with ethyl acetate. The organic phase collected after the extraction of the acid phase was dried over sodium sulphate and the solvent was removed under vacuum.

The crude was purified on silica gel (Toluene/Hexane=8/2 1% acetic acid) obtaining the product **4b** as white crystals. Y% = 60% (200 mg) over 2 steps.

^{1}H NMR (400 MHz, Chloroform-*d*) δ 7.35 – 7.22 (m, 12H), 7.21 – 7.04 (m, 2H), 2.59 (t, *J* = 7.7 Hz, 2H), 2.49 – 2.37 (m, 2H), 1.52 – 1.39 (m, 2H).

^{13}C{^{1}H} NMR (101 MHz, Chloroform-*d*) δ 179.3, 142.2, 141.9, 129.0, 128.4, 128.2, 127.9, 126.9, 125.7, 60.1, 37.4, 36.1, 26.7.

HRMS (ESI-): m/z [M-H]$^-$, calculated for $C_{23}H_{21}O_2^-$: 329.1547; found: 329.1541.

Synthesis of **5e**-*Step 1*

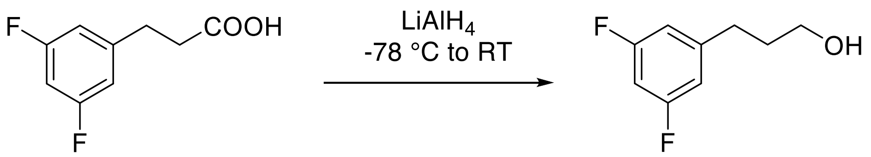

Figure S 7: Synthesis of 3-(3,5-difluorophenyl)propan-1-ol

The carboxylic acid **5a** was reduced to its corrispective alcohol **5b** following the procedure of Natori, Yoshiro et al.(2) obtaining the product in quantitative yield.

The crude resulting mixture was used without further purification for the following step.

MW: 172.17 g/mol

Synthesis of **5e** -*Step 2*

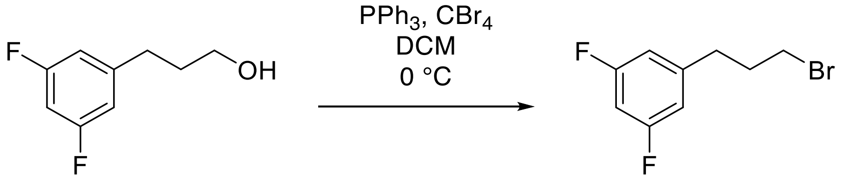

Figure S 8: Synthesis of 1-(3-bromopropyl)-3,5-difluorobenzene

The alcohol **5b** was brominated following the procedure of Natori, Yoshiro et al. (2)

The crude resulting mixture was filtered over a silica pad with hexane as mobile phase, then the solvent was evaporated under vacuum. The product was obtained as a clear oil in quantitative yield.

MW: 235.07 g/mol

Synthesis of **5e** *-Step 3*

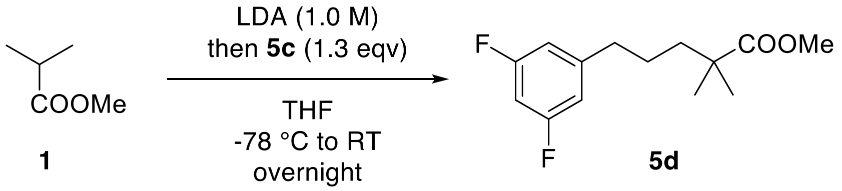

Figure S 9: Synthesis of methyl 5-(3,5-difluorophenyl)-2,2-dimethylpentanoate

1.1 eq. of *n*-BuLi (1 mmol, 625 µl 1,6 M in hexanes) was added to a solution of freshly distilled diisopropylamine (1 eq, 1 mmol, 141 µl) in anhydrous THF (1 ml, 1.0 M) under nitrogen at 0 °C. The reaction mixture was then cooled to -78 °C and ester **1** (0.9 eq, 0.9 mmol, 228 µl) was carefully added dropwise. The reaction was stirred at the same temperature for 30 min. and then bromide **5c** (1 eq, 235 mg) was added dropwise. The mixture is stirred overnight slowly reaching RT.

The reaction was quenched adding one volume of a saturated solution of ammonium chloride in water. The organic phase was separated and the aqueous phase was extracted with ethyl acetate three times. Organic phases were reunited and washed 2 times with HCl 2 M and one time with brine. The organic phase was collected and dried over sodium sulphate then the solvent was evaporated under vacuum.

The crude was a dark yellow oil and was used without purification of the next step.

MW: 256.29 g/mol

Synthesis of **5e** *-Step 4*

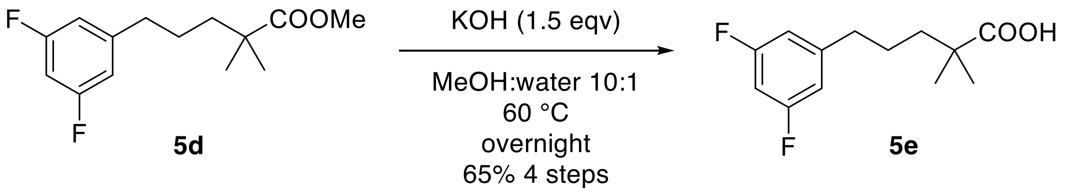

Figure S 10: Synthesis of 5-(3,5-difluorophenyl)-2,2-dimethylpentanoic acid

The crude ester **5d** (530 mg) was dissolved in methanol:water 8:2 (10 ml, 0.1 M). KOH (2 eq, 27.5 mg) was slowly added and the reaction was brought to reflux overnight using an oil bath under magnetic stirring.

The mixture was cooled to RT and the methanol was evaporated under vacuum. The residue was dissolved in water and the aqueous phase was extracted three times with diethyl ether. Aqueous phase was then acidified with HCl 1 M until the pH was 3, then the same phase was extracted three times with ethyl acetate. The organic phase collected after the extraction of the acid phase was dried over sodium sulphate and the solvent was removed under vacuum.

The resulting yellow oil does not need further purification. Y% = 65% (145 mg) over 4 steps.

MW: 242.27 g/mol

^{1}H NMR (400 MHz, Chloroform-*d*) δ 6.81 – 6.56 (m, 3H), 2.61 (t, *J* = 6.7 Hz, 2H), 1.60 (m 2H), 1.22 (s, 6H).

^{13}C{^{1}H} NMR (101 MHz, Chloroform-*d*) δ 184.2, 164.1, 161.8, 161.6, 146.0, 145.9, 111.2, 111.1, 111.0, 110.9, 101.5, 101.2, 101.0, 42.0, 39.7, 35.9, 26.1, 24.9.

HRMS (ESI-): m/z $[M-H]^-$, calculated for $C_{13}H_{15}F_2O_2^-$: 241.1046; found: 241.1043.

Synthesis of **6e**-*Step 1*

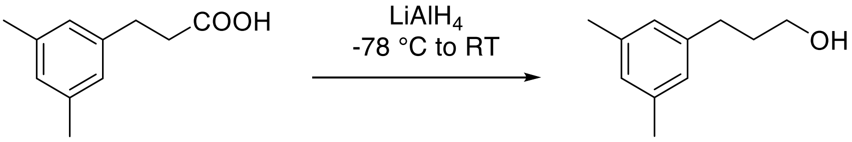

6a **6b**

Figure S 11: Synthesis of 3-(3,5-dimethylphenyl)propan-1-ol

The carboxylic acid **6a** was reduced to its corrispective alcohol **6b** following the procedure of Cheung, Fung K.; et al. (3) obtaining the product in quantitative yield.

The crude resulting mixture was used without further purification for the following step.

^{1}H-NMR analysis matches with the one reported in literature by Cheung, Fung K.; et al. (3)

Synthesis of **6e**-*Step 2*

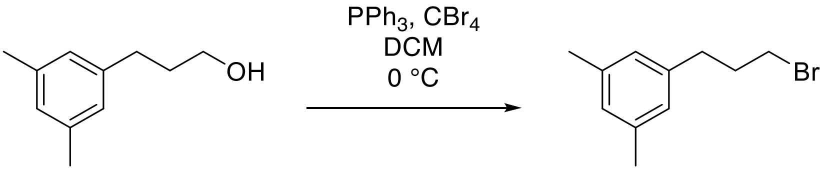

6b **6c**

Figure S 12: Synthesis of 1-(3-bromopropyl)-3,5-dimethylbenzene

The alcohol **6b** was brominated following the procedure of Herold, Sebastian; et al. (4) obtaining the bromide **6c**.

The crude resulting mixture was filtered over a silica pad with hexane as mobile phase, then the solvent was evaporated under vacuum. The product was obtained as a clear oil in quantitative yield.

^{1}H-NMR analysis matches with the one reported in literature by Herold, Sebastian; et al. (4)

Synthesis of **6e** *-Step 3*

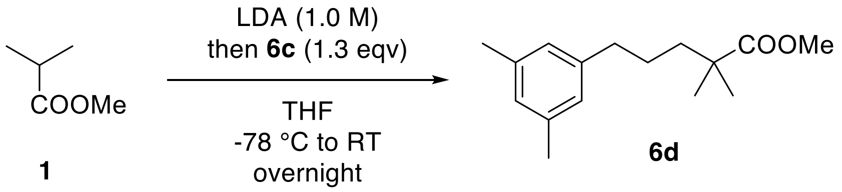

Figure S 13: Synthesis of methyl 5-(3,5-dimethylphenyl)-2,2-dimethylpentanoate

1.1 eq. of *n*-BuLi (1 mmol, 625 µl 1,6 M in hexanes) was added to a solution of freshly distilled diisopropylamine (1 eq, 1 mmol, 141 µl) in anhydrous THF (1 ml, 1.0 M) under nitrogen at 0 °C. The reaction mixture was then cooled to -78 °C and ester **1** (0.9 eq, 0.9 mmol, 228 µl) was carefully added dropwise. The reaction was stirred at the same temperature for 30 min. and then bromide **6c** (1 eq, 227 mg) was added dropwise. The mixture is stirred overnight slowly reaching RT.

The reaction was quenched adding one volume of a saturated solution of ammonium chloride in water. The organic phase was separated and the aqueous phase was extracted with ethyl acetate three times. Organic phases were reunited and washed 2 times with HCl 2 M and one time with brine. The organic phase was collected and dried over sodium sulphate then the solvent was evaporated under vacuum.

The crude mixture was used for the step 4 without further purification.

Synthesis of **6e** *-Step 4*

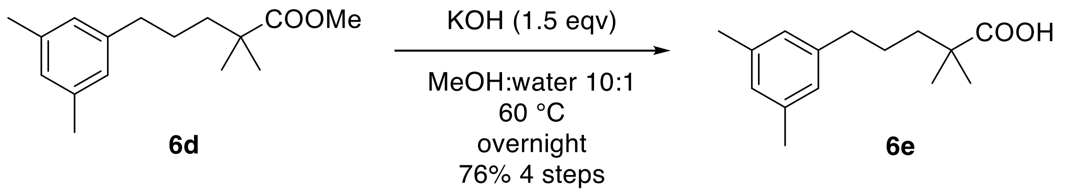

Figure S 14: Synthesis of 5-(3,5-dimethylphenyl)-2,2-dimethylpentanoic acid

The crude ester **6d** (485 mg) was dissolved in methanol:water 8:2 (10 ml, 0.1 M). KOH (2 eq, 27.5 mg) was slowly added and the reaction was brought to reflux overnight using an oil bath under magnetic stirring.

The mixture was cooled to RT and the methanol was evaporated under vacuum. The residue was dissolved in water and the aqueous phase was extracted three times with diethyl ether. Aqueous phase was then acidified with HCl 1 M until the pH was 3, then the same phase was extracted three times with ethyl acetate. The organic phase collected after the extraction of the acid phase was dried over sodium sulphate and the solvent was removed under vacuum.

6e was a yellow oil that does not need further purification. Y% = 79% (185 mg) over 4 steps.

^{1}H NMR (400 MHz, Chloroform-*d*) δ 6.85 (d, J = 9.4 Hz, 3H), 2.56 (dq, J = 4.2, 2.4 Hz, 2H), 2.33 (s, 6H), 1.65 (d, J = 3.9 Hz, 4H), 1.24 (s, 6H).

^{13}C{^{1}H} NMR (101 MHz, Chloroform-*d*) δ 184.9, 142.2, 137.8, 127.4, 126.2, 42.1, 40.2, 36.2, 26.8, 24.9, 21.2.

HRMS (ESI-): m/z $[M-H]^-$, calculated for $C_{15}H_{21}O_2^-$: 233.1547; found: 233.1542.

Synthesis of **7e** *-Step 1*

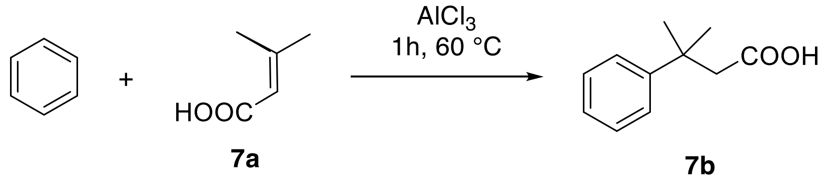

Figure S 15: Synthesis of 3-methyl-3-phenylbutanoic acid

The carboxylic acid **7a** was synthesized following the procedure by Nieman, James A.; et al. (5)

The crude was used without further purification for step 2.

^{1}H-NMR analysis matches with the one reported by Nieman, James A.; et al. (5)

Synthesis of **7e**-*Step 2*

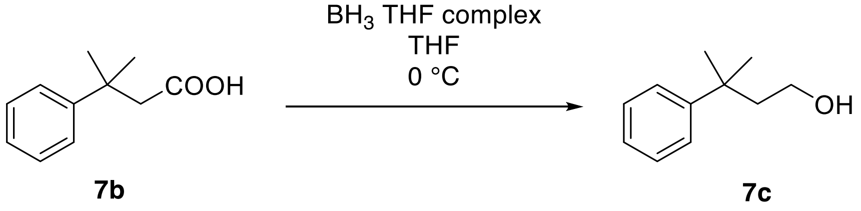

Figure S 16: Synthesis of 3-methyl-3-phenylbutan-1-ol

The alcohol **7b** was synthesized following the procedure by Bartkovitz, David Joseph; et al (6).

The crude was used without further purification for step 2.

^{1}H-NMR analysis matches with the one reported by Bartkovitz, David Joseph; et al (6)

Synthesis of **7e**-*Step 3*

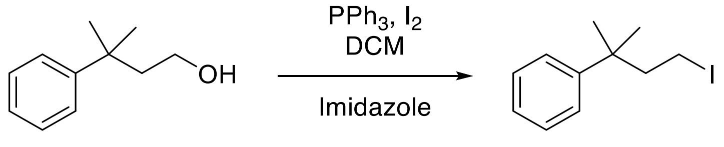

7c **7d**

Figure S 17: Synthesis of (4-iodo-2-methylbutan-2-yl)benzene

The alcohol **7c** was iodinated following the procedure of Chen, Weiqiang; et al. (7) obtaining the iodide **7d**.

Molecular iodine (1.3 eq, 6.5 mmol, 1,6 g) was added portionwise to a solution of PPh_3 (1.3 eq, 6.5 mmol, 1.7 g) in CH_2Cl_2 (32 ml, 0.2 M) at 0 °C. The resulting suspension was stirred for 2 hours at 0 °C. A solution of alcohol **7c** (1.0 eq, 5 mmol, 822 mg) and imidazole (1.3 eq, 6.5 mmol, 446 mg) in CH_2Cl_2 (3 ml, 1.7 M) was added dropwise to the reaction mixture. The reaction was stirred overnight until RT. The reaction was the quenched adding $Na_2S_2O_3$ (1.0 M, aq.) and the aqueous phase was extracted three times with MTBE (3 x 2 volumes). The collected organic phases were washed with 2 volumes of brine and the residue was dried over sodium sulphate, then the solvent was evaporated under reduced pressure.

The crude resulting mixture was filtered over a silica pad with hexane as mobile phase, then the solvent was evaporated under vacuum. The product was obtained as a clear oil. Y% = 78% (1.07 g)

^{1}H NMR (400 MHz, Chloroform-*d*) δ 7.34 (d, *J* = 5.3 Hz, 4H), 7.26 – 7.20 (m, 1H), 2.98 – 2.83 (m, 2H), 2.40 – 2.27 (m, 2H), 1.36 (s, 6H).

^{13}C{^{1}H} NMR (101 MHz, Chloroform-*d*) δ 146.2, 127.4, 125.0, 124.7, 48.6, 39.3, 27.5·

Synthesis of **7e**-*Step 4*

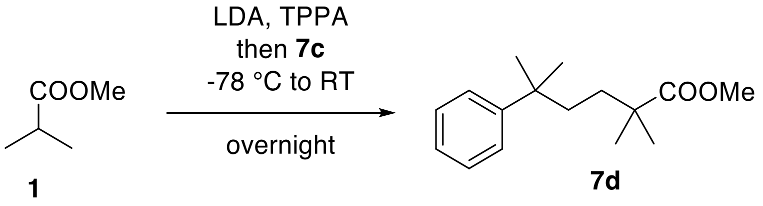

Figure S 18: Synthesis of methyl 2,2,5-trimethyl-5-phenylhexanoate

1.1 eq. of *n*-BuLi (3.3 mmol, 1.88 ml 1,6 M in hexanes) was added to a solution of freshly distilled diisopropylamine (1 eq, 3 mmol, 423 μl) in anhydrous THF (3 ml, 1.0 M) under nitrogen at 0 °C. The reaction mixture was then cooled to -78 °C and a solution of ester **1** (0.9 eq, 2.7 mmol, 431.4 μl) and TPPA (1.05 eq, 3.15 mmol, 783 μl) in dry THF (3 ml, 1.1 M) was carefully added dropwise. The reaction was stirred at the same temperature for 30 min. and then iodide **7c** (1 eq, 3 mmol, 822 mg) was added dropwise. The mixture is stirred overnight slowly reaching RT.

The reaction was quenched adding one volume of a saturated solution of ammonium chloride in water. The organic phase was separated and the aqueous phase was extracted with ethyl acetate three times. Organic phases were reunited and washed 2 times with HCl 2 M and one time with brine. The organic phase was collected and dried over sodium sulphate then the solvent was evaporated under vacuum.

The crude was purified on silica gel using 98:2 Hexane/EtOAc as mobile phase obtaining ester **7d**. The resulting yellow oil was used for the step 5. Y% : 77% (573 mg).

^{1}H NMR (400 MHz, Chloroform-*d*) δ 7.32 (d, *J* = 4.3 Hz, 4H), 7.20 (q, *J* = 4.3 Hz, 1H), 3.64 (d, *J* = 0.9 Hz, 3H), 1.62 – 1.52 (m, 4H), 1.31 (m, 8H), 1.11 (s, 6H).

^{13}C{^{1}H} NMR (101 MHz, Chloroform-*d*) δ 178.4, 149.0, 128.0, 125.8, 125.4, 51.5, 42.1, 39.1, 37.3, 35.5, 28.9, 25.0.

Synthesis of **7e**-*Step 5*

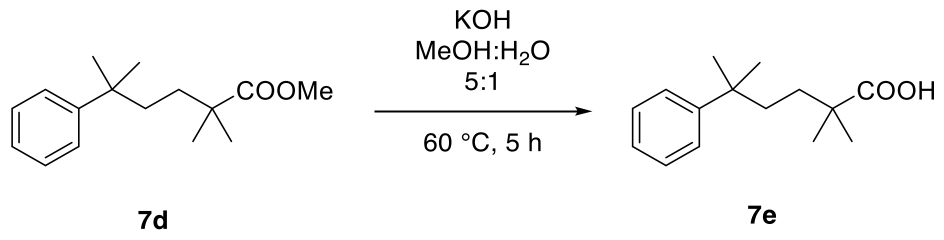

Figure S 19: Synthesis of 2,2,5-trimethyl-5-phenylhexanoic acid

The ester **7d** (500 mg) was dissolved in methanol:water 8:2 (30 ml, 0.1 M). KOH (2 eq, 259.1 mg) was slowly added and the reaction was brought to reflux overnight using an oil bath under magnetic stirring.

The mixture was cooled to RT and the methanol was evaporated under vacuum. The residue was dissolved in water and the aqueous phase was extracted three times with diethyl ether. Aqueous phase was then acidified with HCl 1 M until the pH was 3, then the same phase was extracted three times with ethyl acetate. The organic phase collected after the extraction of the acid phase was dried over sodium sulphate and the solvent was removed under vacuum.

The resulting white powder was obtained in a quantitative yield as **7e** and it does not need further purification.

^{1}H NMR (400 MHz, Chloroform-*d*) δ 7.39 – 7.30 (m, 4H), 7.21 (td, *J* = 5.9, 2.8 Hz, 1H), 1.73 – 1.60 (m, 2H), 1.34 (m, 8H), 1.16 (s, 6H).

^{13}C{^{1}H} NMR (101 MHz, Chloroform-*d*) δ 184.6, 149.0, 128.1, 125.8, 125.5, 41.9, 38.9, 37.4, 35.3, 28.9, 24.8.

HRMS (ESI-): m/z [M-H]$^-$, calculated for $C_{15}H_{21}O_2^-$: 233.1547; found: 233.1542.

Synthesis of **8g** – *step 1*

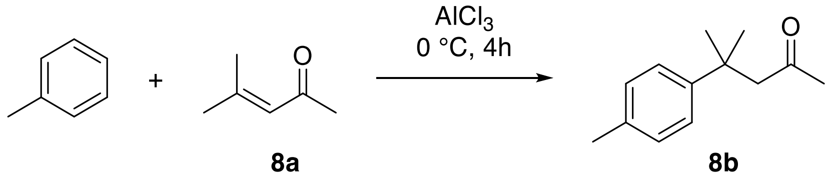

Figure S 20: Synthesis of 4-methyl-4-(p-tolyl)pentan-2-one

Ketone **8b** has been synthesized and purified following the procedure by Hao, Hong-Yan et al. (8)

^{1}H NMR analysis was consistent to the one reported by Hao, Hong-Yan et al. (8)

MW = 190.29 g/mol

Synthesis of **8g** – *step 2*

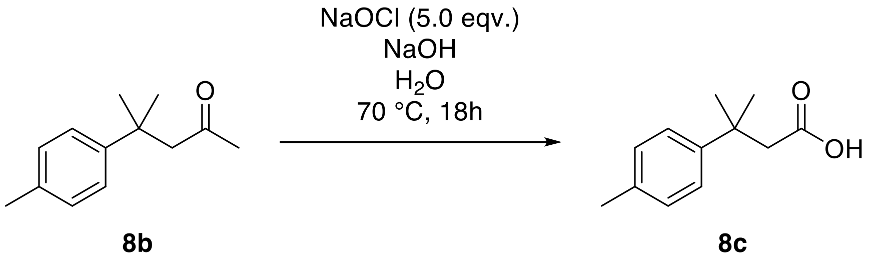

Figure S 21: Synthesis of 3-methyl-3-(p-tolyl)butanoic acid

Carboxylic acid **8c** has been synthesized and purified following the procedure by Dabrowski, J. A. et al. (9).

A flask equipped with stirring bar was charged with ketone **8b** (200 mg, 1.05 mmol, 1 eq). NaOCl 4-6% (aq.) (5.0 eq, 8 mL), NaOH (aq.) (1.1 eq, 2M, 500 µl) and water (50 ml) were

added at RT. The resulting solution was warmed up to 70 °C (oil bath) and stirred for 18 hours. The reaction was allowed to cool to RT and acetone was added until the reaction tested negative for peroxides by starch paper. The reaction was then allowed to cool to 0 °C and concentrated HCl was added dropwise until the solution reached pH 3. The aqueous layer was extracted three times with ethyl acetate. Organic layers was washed with water and brine The organic phase was collected and dried over sodium sulphate, then the solvent was evaporated under vacuum to yield a yellow oil which was purified by crystallization in n-hexane to afford carboxylic acid **8c** as a white solid. Y% = 60 %

^{1}H NMR analysis was consistent to the one reported by Yu, Xiao-Ye; et al. (10)

MW = 192.26 g/mol

Synthesis of **8g** – *step 3*

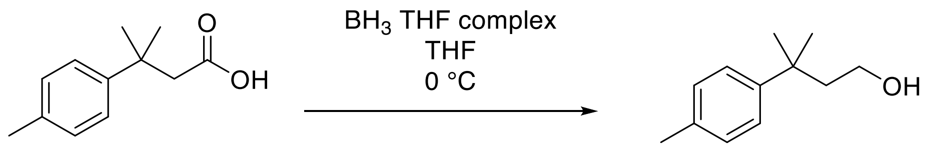

Figure S 22: Synthesis of 3-methyl-3-(p-tolyl)butan-1-ol

The alcohol **8d** was synthesized following the procedure by Bartkovitz, David Joseph; et al. (6)

To a solution of carboxylic acid **8c** (100 mg, 0.52 mmol, 1 eq) in anhydrous tetrahydrofuran (5 ml, 0.02 M) at 0°C was added dropwise a tetrahydrofuran solution of BH_3·THF (2 eq, 1.04 mmol, 1.04 ml, 1 M) under nitrogen. The reaction mixture was stirred at room temperature for 3 h. The mixture was cooled to 0°C and satured bicarbonate was added. The residue was partitioned between ethyl acetate and water. The organic layer were separated,

washed with water, aqueous HCl solution (1 M), brine. The organic phase was dried over sodium sulphate, then the solvent was evaporated under vacuum.

Alcohol **8d** was obtained as colorless oil in a quantitative yield. No further purification was required.

MW = 178.28 g/mol

^{1}H NMR (400 MHz, Chloroform-*d*) δ 7.27 (d, *J* = 8.3 Hz, 2H), 7.15 (dt, *J* = 8.0, 0.7 Hz, 2H), 3.58 – 3.46 (m, 2H), 2.34 (s, 3H), 1.96 (dd, *J* = 7.7, 6.9 Hz, 2H), 1.36 (s, 6H).

^{13}C {^{1}H} NMR (101 MHz, Chloroform-*d*) δ 145.7, 135.2, 128.9, 125.5, 60.2, 46.9, 36.3, 29.3, 20.8.

Synthesis of **8g** – *step 4*

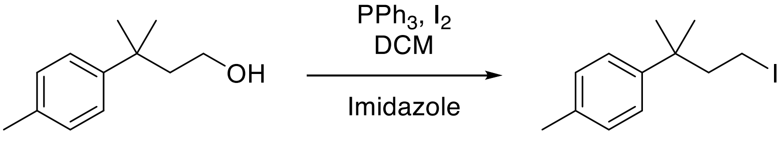

Figure S 23: Synthesis of 1-(4-iodo-2-methylbutan-2-yl)-4-methylbenzene

The alcohol **8d** was iodinated following the procedure of Chen, Weiqiang; et al. (7) obtaining the iodide **8e**.

Molecular iodine (1.3 eq, 4.87 mmol, 1.23 g) was added portionwise to a solution of PPh_3 (1.3 eq, 4.87 mmol, 1.27 g) in CH_2Cl_2 (30 ml, 0.2 M) at 0 °C. The resulting suspension was stirred for 2 hours at 0 °C. A solution of alcohol **8d** (1.0 eq, 3.7 mmol, 668 mg) and imidazole (1.3 eq, 4.87 mmol, 331 mg) in CH_2Cl_2 (2 ml, 1.7 M) was added dropwise to the reaction mixture. The reaction was stirred overnight until RT. The reaction was the quenched adding $Na_2S_2O_3$ (1.0 M, aq.) and the aqueous phase was extracted three times with ethyl acetate (3 x 2 volumes). The collected organic phases were washed with 2

volumes of brine and the residue was dried over sodium sulphate, then the solvent was evaporated under reduced pressure.

The crude resulting mixture was filtered over a silica pad with hexane as mobile phase, then the solvent was evaporated under vacuum. The product was obtained as a clear oil in quantitative yield.

MW = 288.17 g/mol

^{1}H NMR (400 MHz, Chloroform-*d*) δ 7.25 – 7.11 (m, 4H), 2.97 – 2.85 (m, 2H), 2.37 – 2.25 (m, 5H), 1.33 (s, 6H).

^{13}C {^{1}H} NMR (101 MHz, Chloroform-*d*) δ 142.9, 134.1, 127.8, 124.2, 48.3, 38.7, 27.3, 19.5.

Synthesis of **8g** – *step 5*

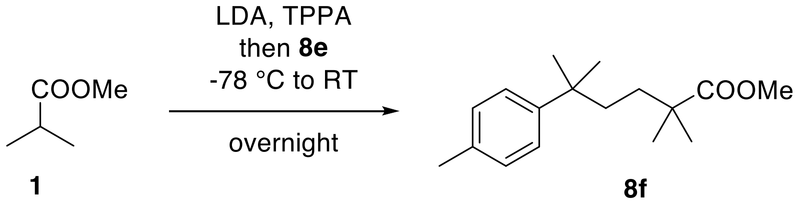

Figure S 24: Synthesis of methyl 2,2,5-trimethyl-5-(p-tolyl)hexanoate

1.1 eq. of *n*-BuLi (2.58 mmol, 1.61 ml 1,6 M in hexanes) was added to a solution of freshly distilled diisopropylamine (1 eq, 1.91 mmol, 270 µl) in anhydrous THF (2 ml, 1.0 M) under nitrogen at 0 °C. The reaction mixture was then cooled to -78 °C and a solution of ester **1** (0.9 eq, 1.7 mmol, 270 µl) and TPPA (1.05 eq, 2.0 mmol, 460 µl) in dry THF (2 ml, 1.1 M) was carefully added dropwise. The reaction was stirred at the same temperature for 30 min. and then iodide **8e** (1 eq, 1.9 mmol, 550 mg) was added dropwise. The mixture is stirred overnight slowly reaching RT.

The reaction was quenched adding one volume of a saturated solution of ammonium chloride in water. The organic phase was separated and the aqueous phase was extracted with ethyl acetate three times. Organic phases were reunited and washed 2 times with HCl 2 M and one time with brine. The organic phase was collected and dried over sodium sulphate then the solvent was evaporated under vacuum.

The crude was purified on silica gel using 98:2 Hexane/EtOAc as mobile phase obtaining ester **8f**. The resulting yellow oil was used for the step 6. Y% = 80%.

MW = 262.39 g/mol

^{1}H NMR (400 MHz, Chloroform-*d*) δ 7.21 (d, *J* = 8.3 Hz, 2H), 7.14 (d, *J* = 0.8 Hz, 2H), 3.65 (s, 3H), 2.35 (s, 3H), 1.57 – 1.51 (m, 2H), 1.34 – 1.25 (m, 8H), 1.11 (s, 6H).

^{13}C{^{1}H} NMR (101 MHz, Chloroform-*d*) δ 178.4, 146.0, 134.8, 128.7, 125.6, 51.5, 42.1, 39.1, 37.0, 35.5, 29.0, 25.1, 20.8.

Synthesis of **8g** – *step 6*

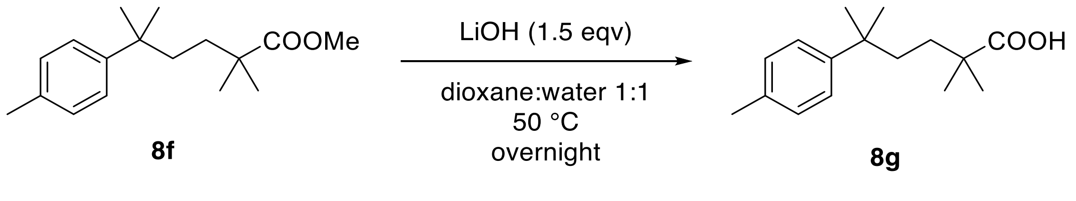

Figure S 25: Synthesis of 2,2,5-trimethyl-5-(p-tolyl)hexanoic acid

Ester **8f** (1 eq, 1 mmol, 255 mg) was dissolved in a 1:1 mixture water:1,4 dioxane (10 ml, 0.1 M). LiOH (1.5 eq, 200 mg) was added to the reaction mixture and the temperature was brought to 50 °C using an oil bath under magnetic stirring for one night.

After the mixture was cooled to RT, 1,4 dioxane was removed under vacuum. The residue was washed three times with diethyl ether. Aqueous phase was then acidified with HCl 1 M

(aq) until the pH was 3, then the same phase was extracted three times with ethyl acetate. The organic phase collected after the extraction of the acid phase was dried over sodium sulphate and the solvent was removed under vacuum.

The product **8g** results as white crystals in quantitative yield and no further purification was required.

MW = 248.37 g/mol

^{1}H NMR (400 MHz, Chloroform-*d*) δ 7.20 (d, *J* = 8.2 Hz, 2H), 7.11 (d, *J* = 8.0 Hz, 2H), 2.32 (s, 3H), 1.66 – 1.53 (m, 2H), 1.36 – 1.29 (m, 2H), 1.27 (s, 7H), 1.12 (s, 6H).

^{13}C{^{1}H} NMR (101 MHz, Chloroform-*d*) δ 183.0, 146.0, 134.8, 128.8, 125.7, 41.8, 38.9, 37.0, 35.3, 29.0, 24.9, 20.9.

HRMS (ESI-): m/z [M-H]$^-$, calculated for $C_{16}H_{23}O_2^-$: 247.1704; found: 247.1708.

Synthesis of **9e** *– step 1*

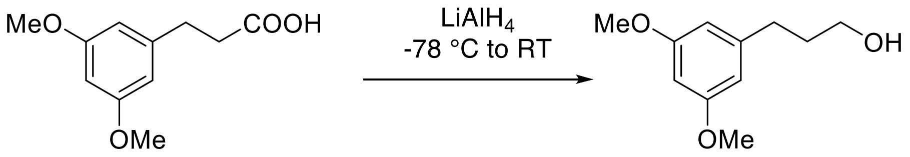

9a **9b**

Figure S 26: Synthesis of 3-(3,5-dimethoxyphenyl)propan-1-ol

The carboxylic acid **9a** was reduced to its corrispective alcohol **9b** following the procedure of Plamondon et al. (12) obtaining the product in quantitative yield.

The crude resulting mixture was used without further purification for the following step.

^{1}H-NMR analysis matches with the one reported in literature by Plamondon et al. (12).

Synthesis of **9e** *– step 2*

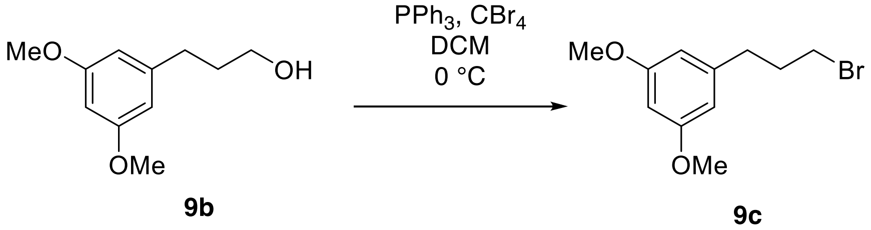

Figure S 27: Synthesis of 1-(3-bromopropyl)-3,5-dimethoxybenzene

The alcohol **9b** was brominated following the procedure of Göksu et al. (13) obtaining the bromide **9c**.

The crude resulting mixture was filtered over a silica pad with hexane as mobile phase, then the solvent was evaporated under vacuum. The product was obtained as a clear oil in quantitative yield.

^{1}H-NMR analysis matches with the one reported in literature by Göksu et al. (13).

Synthesis of **9e** *– step 3*

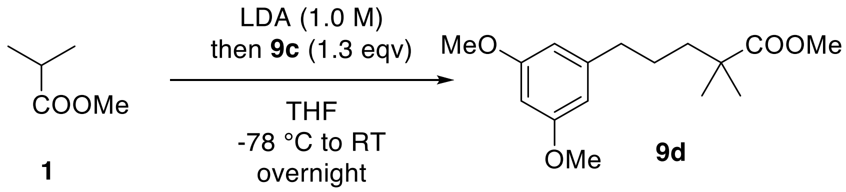

Figure S 28: Synthesis of methyl 5-(3,5-dimethoxyphenyl)-2,2-dimethylpentanoate

1.1 eq. of *n*-BuLi (1 mmol, 625 µl 1,6 M in hexanes) was added to a solution of freshly distilled diisopropylamine (1 eq, 1 mmol, 141 µl) in anhydrous THF (1 ml, 1.0 M) under nitrogen at 0 °C. The reaction mixture was then cooled to -78 °C and ester **1** (0.9 eq, 0.9 mmol, 228 µl) was carefully added dropwise. The reaction was stirred at the same

temperature for 30 min. and then bromide **9c** (1 eq, 257 mg) was added dropwise. The mixture is stirred overnight slowly reaching RT.

The reaction was quenched adding one volume of a saturated solution of ammonium chloride in water. The organic phase was separated and the aqueous phase was extracted with ethyl acetate three times. Organic phases were reunited and washed 2 times with HCl 2 M and one time with brine. The organic phase was collected and dried over sodium sulphate then the solvent was evaporated under vacuum.

The crude mixture was used for the step 4 without further purification.

Synthesis of **9e** *– step 4*

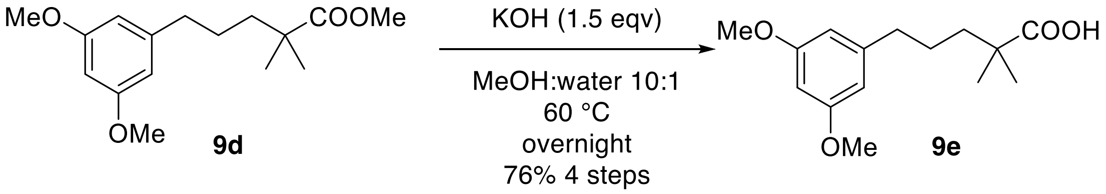

Figure S 29: Synthesis of 5-(3,5-dimethoxyphenyl)-2,2-dimethylpentanoic acid

The crude ester **9d** (471 mg) was dissolved in methanol:water 10:1 (10 ml, 0.1 M). KOH (2 eq, 27.5 mg) was slowly added and the reaction was brought to reflux overnight using an oil bath under magnetic stirring.

The mixture was cooled to RT and the methanol was evaporated under vacuum. The residue was dissolved in water and the aqueous phase was extracted three times with diethyl ether. Aqueous phase was then acidified with HCl 1 M until the pH was 3, then the same phase was extracted three times with ethyl acetate. The organic phase collected after the extraction of the acid phase was dried over sodium sulphate and the solvent was removed under vacuum.

9e was a brown oil that does not need further purification. Y% = 71% (215 mg) over 4 steps.

^{1}H NMR (400 MHz, Chloroform-*d*) δ 6.38 (d, *J* = 2.3 Hz, 2H), 6.34 (d, *J* = 2.3 Hz, 1H), 3.81 (s, 6H), 2.59 (d, *J* = 2.3 Hz, 2H), 1.72 – 1.55 (m, 4H), 1.23 (s, 6H).

^{13}C{^{1}H} NMR (101 MHz, Chloroform-*d*) δ 184.9, 160.7, 144.6, 106.5, 97.8, 55.3, 42.0, 40.0, 36.6, 26.4, 24.9.

HRMS (ESI-): m/z $[M-H]^-$, calculated for $C_{15}H_{21}O_4^-$: 265.1445; found: 265.1445.

Synthesis of **10e** *– step 1*

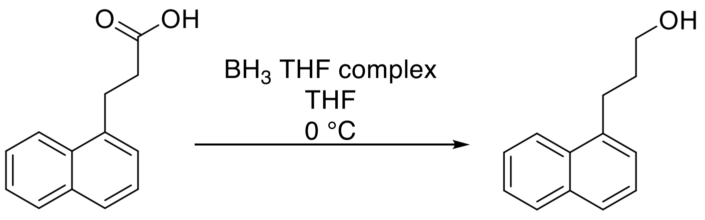

10a **10b**

Figure S 30: Synthesis of 3-(naphthalen-1-yl)propan-1-ol

The alcohol **10b** was synthesized following the procedure by Bartkovitz, David Joseph; et al. (6)

To a solution of carboxylic acid **10a** (1 g, 5 mmol, 1 eq) in anhydrous tetrahydrofuran (50 ml, 0.1 M) at 0°C was added dropwise a tetrahydrofuran solution of BH_3·THF (2 eq, 10 mmol, 10 ml, 1 M) under nitrogen. The reaction mixture was stirred at room temperature for 3 h. The mixture was cooled to 0°C and satured bicarbonate was added. The residue was partitioned between ethyl acetate and water. The organic layer were separated, washed with water, aqueous HCl solution (1 M), brine. The organic phase was dried over sodium sulphate, then the solvent was evaporated under vacuum.

Alcohol **10b** was obtained as colorless oil in a quantitative yield. No further purification was required.

^{1}H-NMR analysis matches with the one reported in literature by Yao, Yi-Xuan et al. (24).

MW = 186.25 g/mol

^{1}H NMR (400 MHz, Chloroform-*d*) δ = 7.99 (d, J = 8.2 Hz, 1 H), 7.84-7.58 (m, 2 H), 7.50-7.12 (m, 4 H), 3.67 (t, J = 6.3 Hz, 2 H), 3.10 (dd, J = 8.6, 6.8 Hz, 2 H), 2.04-1.87 (m, 2 H), 1.24 (d, J = 54.2 Hz, 1 H).

Synthesis of **10e** *– step 2*

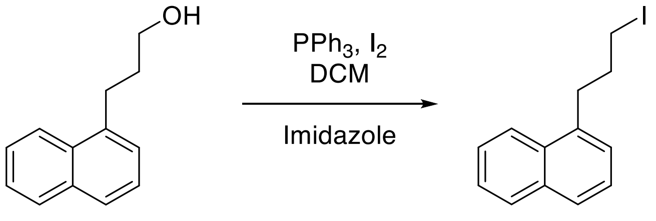

10b **10c**

Figure S 31: Synthesis of 1-(3-iodopropyl)naphthalene

The alcohol **10b** was iodinated following the procedure of Chen, Weiqiang; et al. (7) obtaining the iodide **10c**.

Molecular iodine (1.3 eq, 6.5 mmol, 1.65 g) was added portionwise to a solution of PPh_3 (1.3 eq, 6.5 mmol, 1.7 g) in CH_2Cl_2 (32 ml, 0.2 M) at 0 °C. The resulting suspension was stirred for 2 hours at 0 °C. A solution of alcohol **10b** (1.0 eq, 5 mmol, 930 mg) and imidazole (1.3 eq, 6.5 mmol, 440 mg) in CH_2Cl_2 (3 ml, 1.7 M) was added dropwise to the reaction mixture. The reaction was stirred overnight until RT. The reaction was the

quenched adding $Na_2S_2O_3$ (1.0 M, aq.) and the aqueous phase was extracted three times with ethyl acetate (3 x 2 volumes). The collected organic phases were washed with 2 volumes of brine and the residue was dried over sodium sulphate, then the solvent was evaporated under reduced pressure.

The crude resulting mixture was filtered over a silica pad with hexane as mobile phase, then the solvent was evaporated under vacuum. The product was obtained as a clear oil in quantitative yield.

MW = 296.15 g/mol

^{1}H NMR (400 MHz, Chloroform-*d*) δ 8.07 (dd, *J* = 8.3, 1.4 Hz, 1H), 7.93 – 7.85 (m, 1H), 7.77 (dt, *J* = 7.8, 1.1 Hz, 1H), 7.54 (dddd, *J* = 17.8, 8.1, 6.8, 1.4 Hz, 2H), 7.48 – 7.36 (m, 2H), 3.41 – 3.05 (m, 4H), 2.54 – 2.16 (m, 2H).

^{13}C{^{1}H} NMR (101 MHz, Chloroform-*d*) δ 136.5, 133.9, 131.7, 128.9, 127.0, 126.4, 125.9, 125.6, 123.6, 34.1, 33.5, 6.8.

Synthesis of **10e** *– step 3*

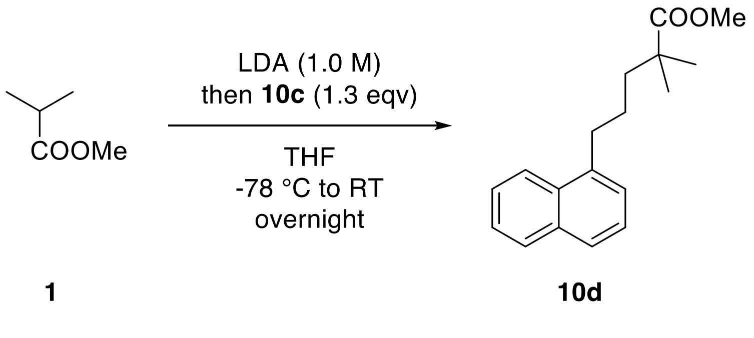

Figure S 32: Synthesis of methyl 2,2-dimethyl-5-(naphthalen-1-yl)pentanoate

1.1 eq. of *n*-BuLi (5.5 mmol, 3.4 ml 1,6 M in hexanes) was added to a solution of freshly distilled diisopropylamine (1 eq, 5 mmol, 722 µl) in anhydrous THF (5 ml, 1.0 M) under nitrogen at 0 °C. The reaction mixture was then cooled to -78 °C and ester **1** (0.9 eq, 4.5 mmol, 510 µl) was carefully added dropwise. The reaction was stirred at the same

temperature for 30 min. and then iodide **10c** (1 eq, 1.48 g) was added dropwise. The mixture is stirred overnight slowly reaching RT.

The reaction was quenched adding one volume of a saturated solution of ammonium chloride in water. The organic phase was separated and the aqueous phase was extracted with ethyl acetate three times. Organic phases were reunited and washed 2 times with HCl 2 M and one time with brine. The organic phase was collected and dried over sodium sulphate then the solvent was evaporated under vacuum.

The crude mixture was purified on silica gel using hexane/EtOAc 9:1 obtaining pure ester **10d** with 63% yield as a clear viscous oil (806 mg).

^{1}H NMR (400 MHz, Chloroform-*d*) δ 8.07 – 8.00 (m, 1H), 7.92 – 7.84 (m, 1H), 7.73 (dt, *J* = 8.2, 1.1 Hz, 1H), 7.61 – 7.45 (m, 2H), 7.42 (dd, *J* = 8.2, 7.0 Hz, 1H), 7.34 (dd, *J* = 6.6, 1.1 Hz, 1H), 3.63 (s, 3H), 3.07 (td, *J* = 5.0, 2.5 Hz, 2H), 1.89 – 1.65 (m, 4H), 1.19 (s, 6H).

^{13}C {^{1}H} NMR (101 MHz, Chloroform-*d*) δ 178.4, 138.3, 133.9, 131.8, 128.7, 126.6, 125.8, 125.7, 125.5, 125.4, 123.7, 51.6, 42.3, 40.8, 33.4, 26.2, 25.2.

HRMS (ESI-): m/z [M+H]$^{+}$, calculated for $C_{18}H_{23}O_2^{+}$: 271.1693; found: 271.1695.

Synthesis of **10e** *– step 4*

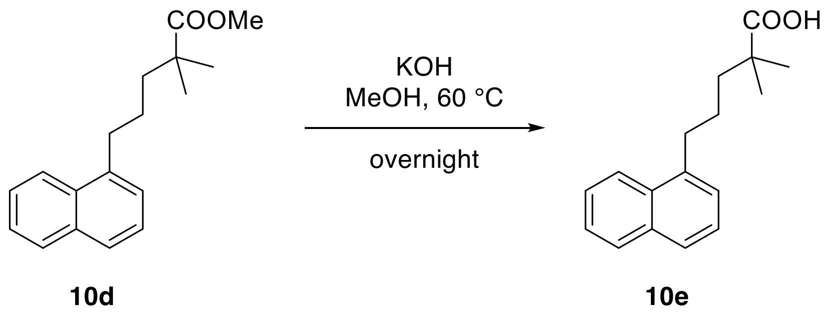

Figure S 33: Synthesis of methyl 2,2-dimethyl-5-(naphthalen-1-yl)pentanoic acid

The ester **10d** (800 mg) was dissolved in methanol:water 10:1 (30 ml, 0.1 M). KOH (2 eq, 170 mg) was slowly added and the reaction was brought to reflux overnight using an oil bath under magnetic stirring.

The mixture was cooled to RT and the methanol was evaporated under vacuum. The residue was dissolved in water and the aqueous phase was extracted three times with diethyl ether. Aqueous phase was then acidified with HCl 1 M until the pH was 3, then the same phase was extracted three times with ethyl acetate. The organic phase collected after the extraction of the acid phase was dried over sodium sulphate and the solvent was removed under vacuum.

10e was obtained as a white solid that does not need further purification in quantitative yield (750 mg).

^{1}H NMR (400 MHz, Chloroform-*d*) δ 7.91 (d, *J* = 8.0 Hz, 1H), 7.80 – 7.69 (m, 1H), 7.61 (d, *J* = 8.1 Hz, 1H), 7.46 – 7.33 (m, 3H), 7.32 – 7.26 (m, 1H), 7.22 (d, *J* = 6.9 Hz, 1H), 2.96 (t, *J* = 6.9 Hz, 2H), 1.72 – 1.59 (m, 4H), 1.10 (d, *J* = 2.0 Hz, 6H).

^{13}C{^{1}H} NMR (101 MHz, Chloroform-*d*) δ 184.9, 138.3, 133.9, 131.9, 128.8, 126.7, 125.9, 125.8, 125.6, 125.5, 123.8, 42.2, 40.5, 33.5, 26.2, 24.9.

HRMS (ESI-): m/z $[M-H]^-$, calculated for $C_{17}H_{19}O_2^-$: 255.1391; found: 255.1390.

Optimization studies on 2b

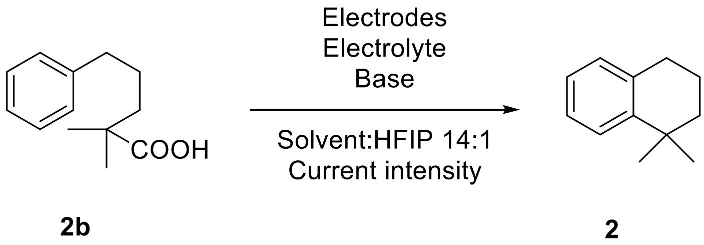

Electrolyte (0.1 M)	Current intensity (mA)	Current quantity (F/mol)	Base (type)	Solvent (X : HFIP 14:1)	Base (eqv)	Substrate concentration (M)	Working electrode	Counter electrode
TBA OAc	1	1.5	Collidine	DCM	1	0.01	GC	C
TBA BF4	2.5	2.1	Lutidine	DMSO	2	0.02	C	Pt
TBA ClO4	5	2.6	Triethylamine	THF	3	0.04	RVC	Ni
TBA PF6	7.5		DIPEA	DMF	4	0.1	Pt	Zn
TBA OH	10		DBU	Acetone	5			SS
			4-MeO-Py	MeCN				
			Quinuclidine					

Table S 1: Chosen parameters for the optimization.

Yields have been calculated via GC-MS using 1,4-diisopropylbenzene as an internal standard.

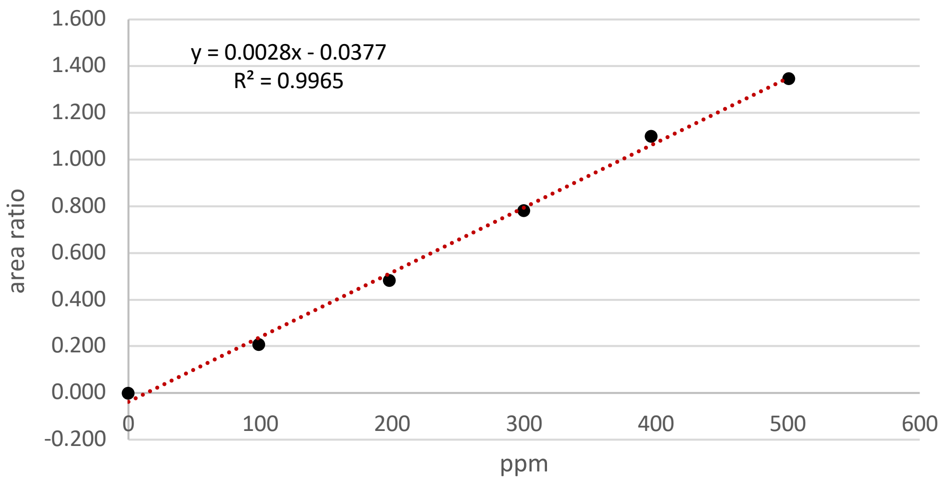

Figure S 34: Calibration line of GC-MS.

General procedure 1 *for batch optimization electrolysis:*

All reactions were performed in an IKA ElectraSyn 2.0 using electrodes purchased from IKA. In a 10 mL IKA ElectraSyn vial, equipped with a stir bar, 1 mmol of electrolyte, 0.2 mmol (1 eqv) of **2b**, 0.7 mL of HFIP, 0.6 mmol of base (3 eqv) were dissolved in 10 mL of dichloromethane and bubbled with Ar while mixed for 5 minutes, than the electrochemical cell was assembled. The instrument was operated under constant current mode at 400 rpm of stirring. After 2.1 F/mol were passed through, the reaction mixture was washed with 1 M HCl (aq.) and the internal standard was added to the organic fraction to measure the yield in GC-MS. All the variations from **General procedure 1** were specified.

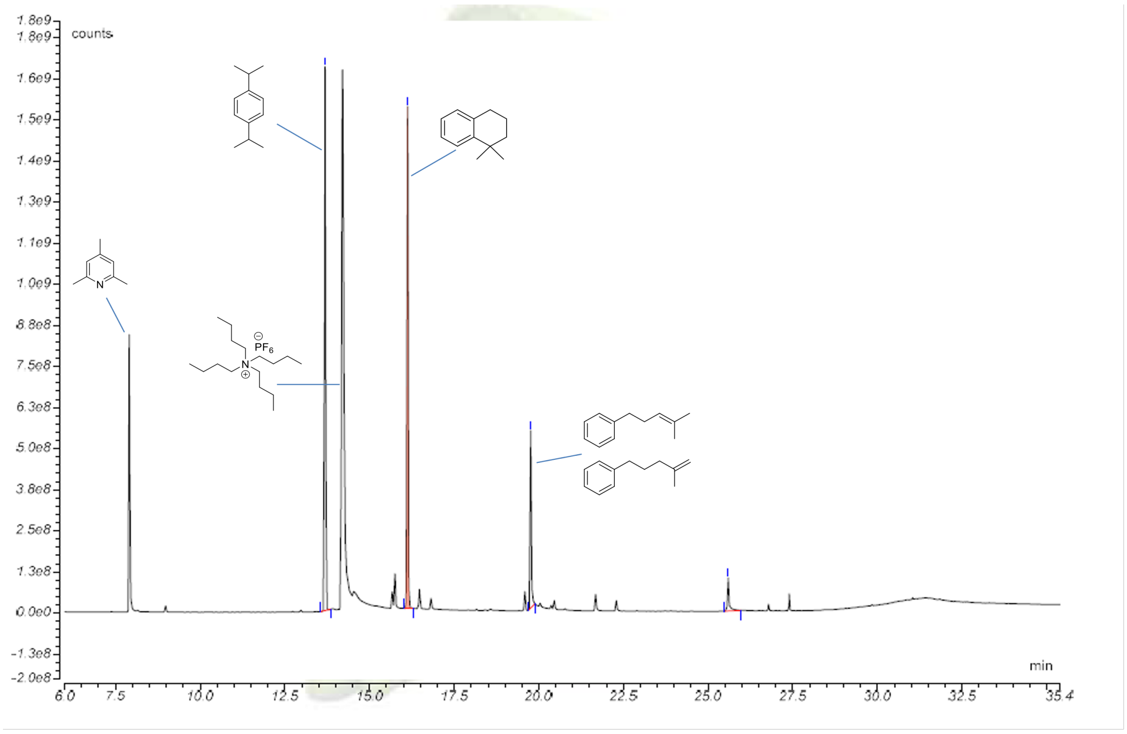

Figure S 35: Typical GC-MS chromatogram of an optimization reaction with the internal standard.

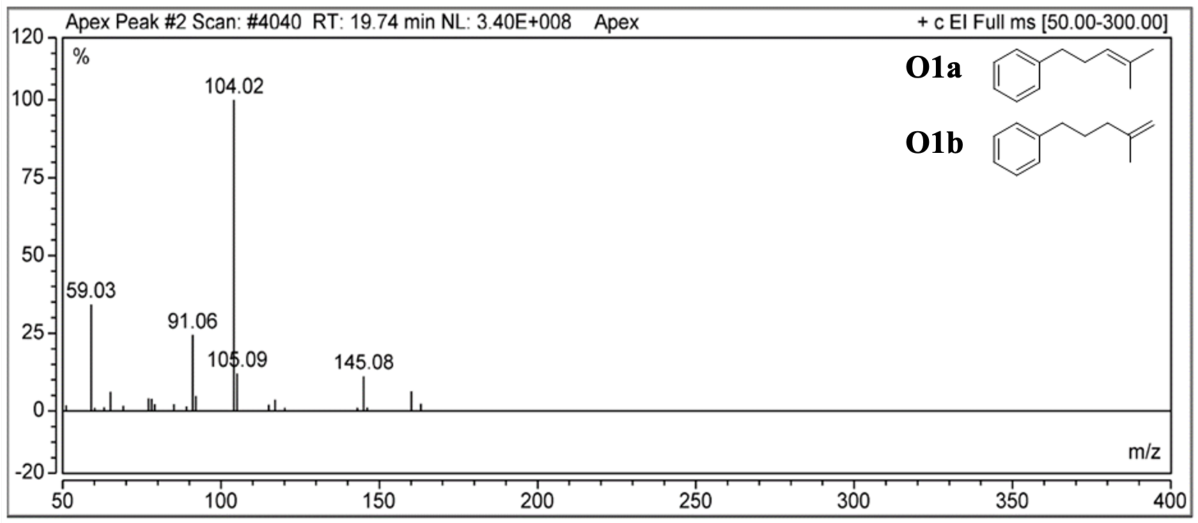

Figure S 36: Mass spectrum of the olefinic byproducts.

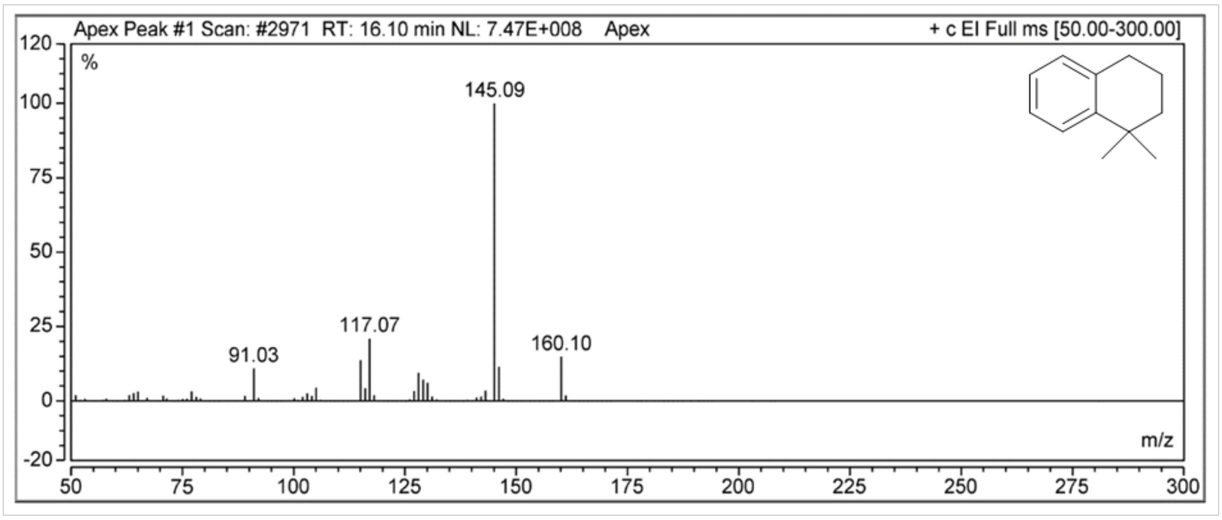

Figure S 37: Mass spectrum of the desired product.

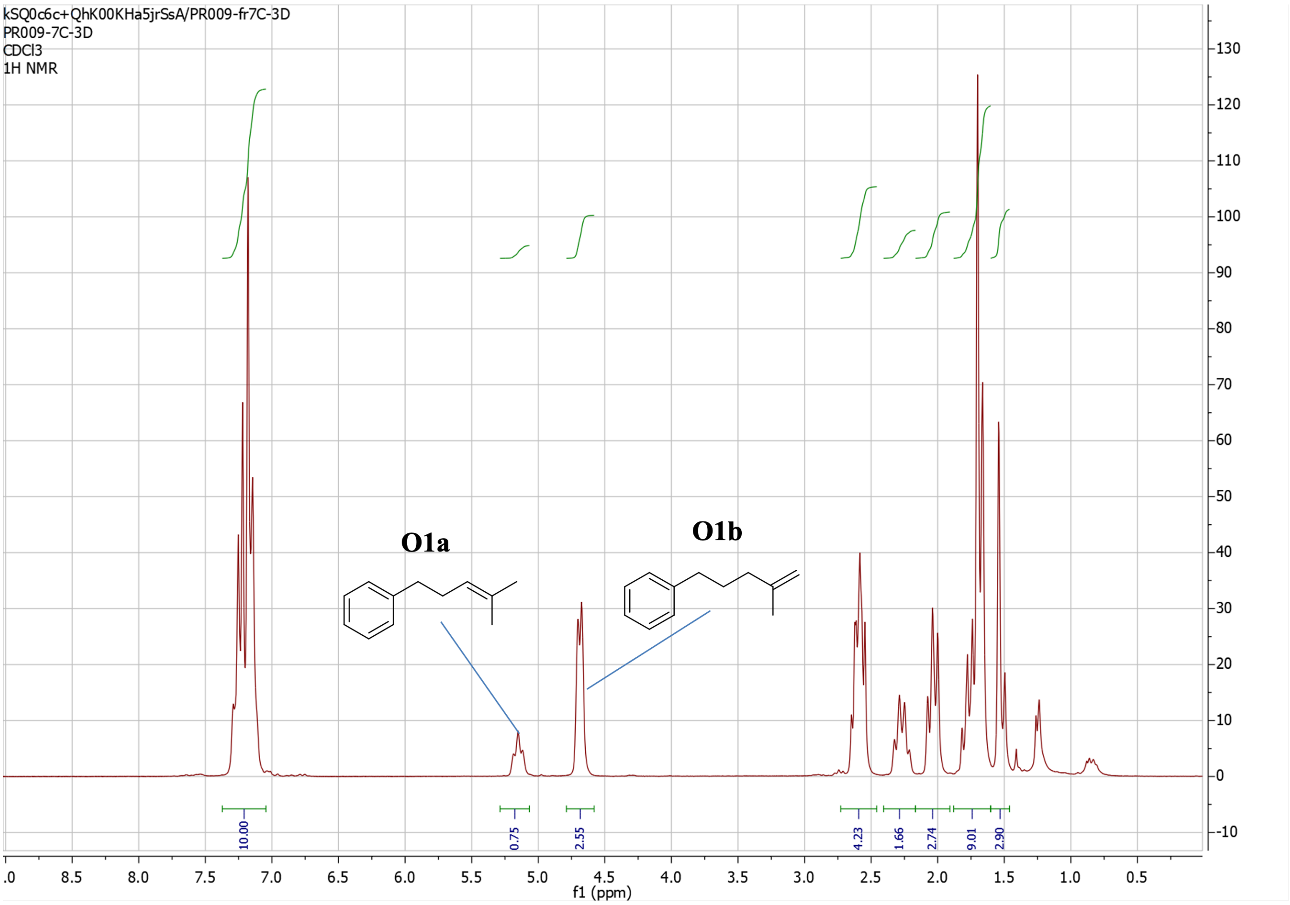

Figure S 38: 1H-NMR spectrum of the olefinic subproducts.

Cyclic voltammetry analysis on **2b**:

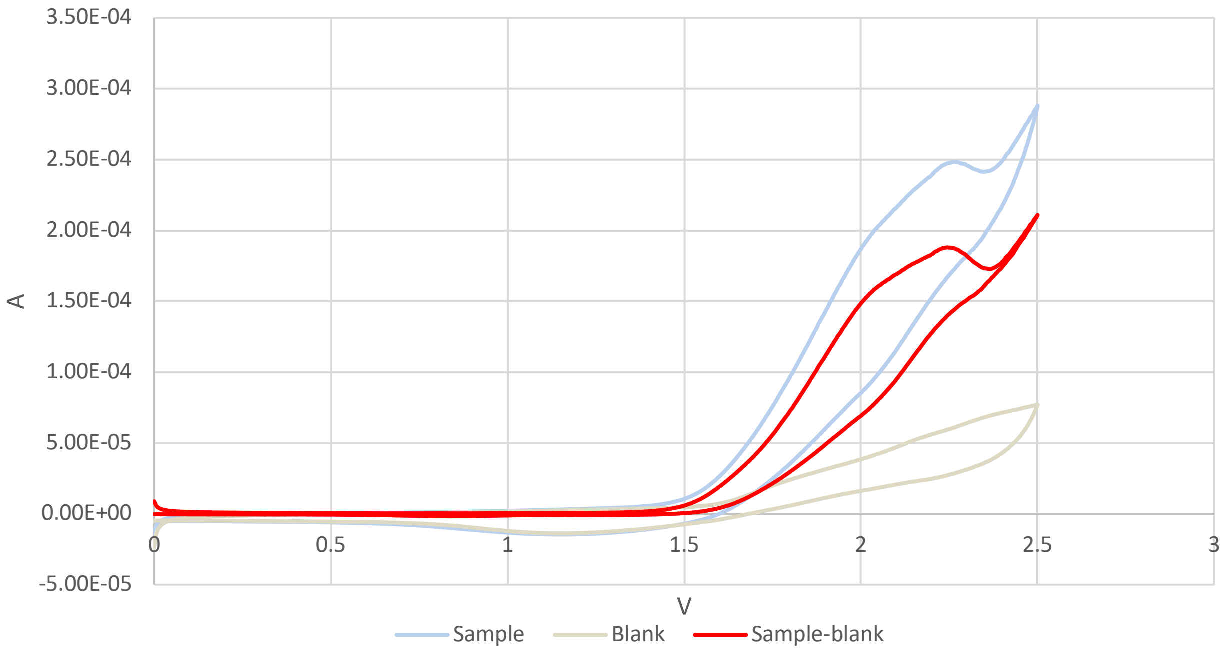

Figure S 39: Cyclic voltammetry analysis of SM. Conditions: 0.1 M *n*-Bu_4NBF_4, 5 mM **2b**, 10 mL DCM:HFIP 14:1, 3 eq 2,4,6-collidine, 50 mV/s.

Optimization of the electrolyte:

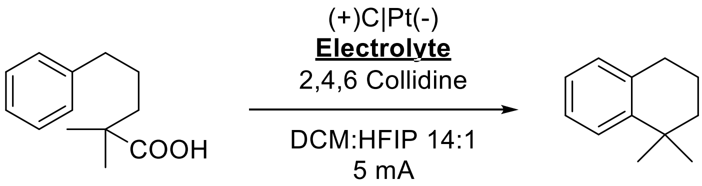

Electrolyte (0.1 M)	Current intensity (mA)	Current quantity (F/mol)	Base (type)	Solvent (X : HFIP 14:1)	Base (eqv)	Substrate concentration (M)	Working electrode	Counter electrode	Yield % (GC/MS)
TBA OAc	5	2	-	DCM	-	0.02	C	Pt	21
TBA BF4	5	2	Collidine	DCM	3	0.02	C	Pt	30
TBA ClO4	5	2	Collidine	DCM	3	0.02	C	Pt	21
TBA PF6	5	2	Collidine	DCM	3	0.02	C	Pt	51
TBA OH	5	2	-	DCM	-	0.02	C	Pt	10

Table S 2: Optimization of the electrolyte.

Optimization of the current intensity:

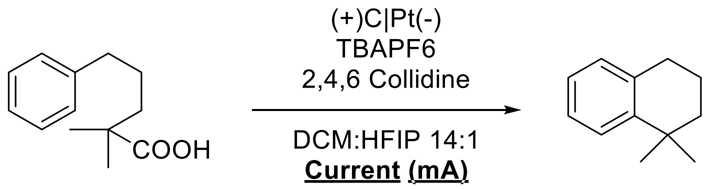

Electrolyte (0.1 M)	Current intensity (mA)	Current quantity (F/mol)	Base (type)	Solvent (X : HFIP 14:1)	Base (eqv)	Substrate concentration (M)	Working electrode	Counter electrode	Yield % (GC/MS)
TBA PF6	1	2	Collidine	DCM	3	0.02	C	Pt	31
TBA PF6	2.5	2	Collidine	DCM	3	0.02	C	Pt	49
TBA PF6	5	2	Collidine	DCM	3	0.02	C	Pt	51
TBA PF6	7.5	2	Collidine	DCM	3	0.02	C	Pt	52
TBA PF6	10	2	Collidine	DCM	3	0.02	C	Pt	38

Table S 3: Optimization of the current intensity.

Optimization of current quantity (F/mol)

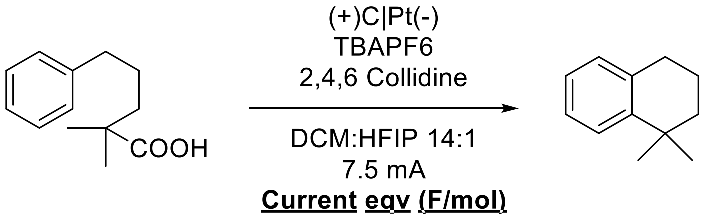

Electrolyte (0.1 M)	Current intensity (mA)	Current quantity (F/mol)	Base (type)	Solvent (X : HFIP 14:1)	Base (eqv)	Substrate concentration (M)	Working electrode	Counter electrode	Yield % (GC/MS)
TBA PF6	7.5	1.5	Collidine	DCM	3	0.02	C	Pt	49
TBA PF6	7.5	2.1	Collidine	DCM	3	0.02	C	Pt	64
TBA PF6	7.5	2.5	Collidine	DCM	3	0.02	C	Pt	48

Table S 4: Optimization of the current quantity.

Optimization of the solvent:

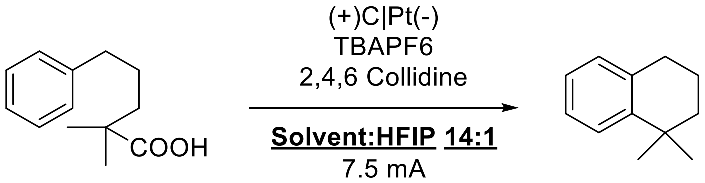

Electrolyte (0.1 M)	Current intensity (mA)	Current quantity (F/mol)	Base (type)	Solvent (X : HFIP 14:1)	Base (eqv)	Substrate concentration (M)	Working electrode	Counter electrode	Yield % (GC/MS)
TBA PF6	7.5	2.1	Collidine	DCM	3	0.02	C	Pt	64
TBA PF6	7.5	2.1	Collidine	DMSO	3	0.02	C	Pt	<5
TBA PF6	7.5	2.1	Collidine	DMF	3	0.02	C	Pt	<5
TBA PF6	7.5	2.1	Collidine	THF	3	0.02	C	Pt	<5
TBA PF6	7.5	2.1	Collidine	ACETONE	3	0.02	C	Pt	<5
TBA PF6	7.5	2.1	Collidine	MeCN	3	0.02	C	Pt	<5

Table S 5: Optimization of the solvent.

Optimization of the base type:

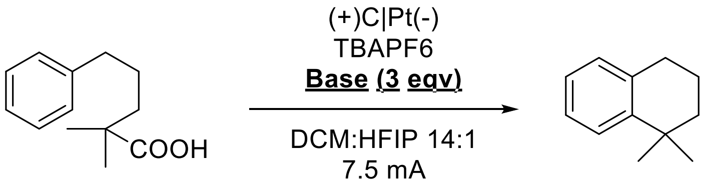

Electrolyte (0.1 M)	Current intensity (mA)	Current quantity (F/mol)	Base (type)	Solvent (X : HFIP 14:1)	Base (eqv)	Substrate concentration (M)	Working electrode	Counter electrode	Yield % (GC/MS)
TBA PF6	7.5	2.1	Collidine	DCM	3	0.02	C	Pt	64
TBA PF6	7.5	2.1	Lutidine	DCM	3	0.02	C	Pt	43
TBA PF6	7.5	2.1	TEA	DCM	3	0.02	C	Pt	<5
TBA PF6	7.5	2.1	DIPEA	DCM	3	0.02	C	Pt	<5
TBA PF6	7.5	2.1	DBU	DCM	3	0.02	C	Pt	46
TBA PF6	7.5	2.1	4-MeO-Py	DCM	3	0.02	C	Pt	53
TBA PF6	7.5	2.1	Quinuclidine	DCM	3	0.02	C	Pt	-

Table S 6: Optimization of the type of base.

Optimization of base equivalents:

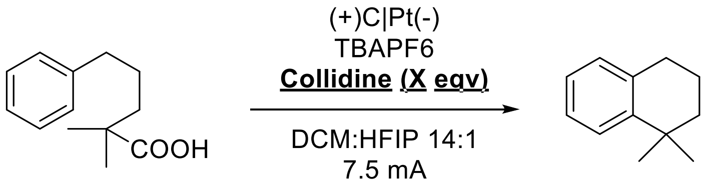

Electrolyte (0.1 M)	Current intensity (mA)	Current quantity (F/mol)	Base (type)	Solvent (X : HFIP 14:1)	Base (eqv)	Substrate concentration (M)	Working electrode	Counter electrode	Yield % (GC/MS)
TBA PF6	7.5	2.1	Collidine	DCM	0.5	0.02	C	Pt	15
TBA PF6	7.5	2.1	Collidine	DCM	1	0.02	C	Pt	40
TBA PF6	7.5	2.1	Collidine	DCM	1.5	0.02	C	Pt	54
TBA PF6	7.5	2.1	Collidine	DCM	2	0.02	C	Pt	64
TBA PF6	7.5	2.1	Collidine	DCM	3	0.02	C	Pt	64
TBA PF6	7.5	2.1	Collidine	DCM	4	0.02	C	Pt	45
TBA PF6	7.5	2.1	Collidine	DCM	5	0.02	C	Pt	45

Table S 7: Optimization of the equivalents of base.

Optimization of the working electrode:

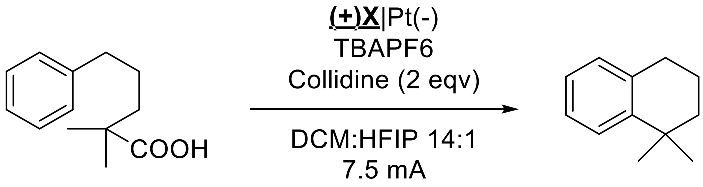

Electrolyte (0.1 M)	Current intensity (mA)	Current quantity (F/mol)	Base (type)	Solvent (X : HFIP 14:1)	Base (eqv)	Substrate concentration (M)	Working electrode	Counter electrode	Yield % (GC/MS)
TBA PF6	7.5	2.1	Collidine	DCM	2	0.02	C	Pt	64
TBA PF6	7.5	2.1	Collidine	DCM	2	0.02	GC	Pt	-
TBA PF6	7.5	2.1	Collidine	DCM	2	0.02	Pt	Pt	-
TBA PF6	7.5	2.1	Collidine	DCM	2	0.02	RVC	Pt	-

Table S 8: Optimization of the working electrode material.

Optimization of counter electrode:

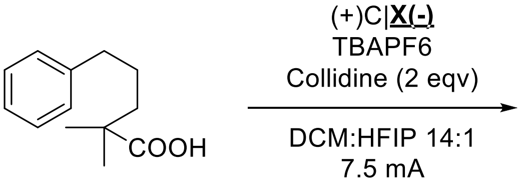

Electrolyte (0.1 M)	Current intensity (mA)	Current quantity (F/mol)	Base (type)	Solvent (X : HFIP 14:1)	Base (eqv)	Substrate concentration (M)	Working electrode	Counter electrode	Yield % (GC/MS)
TBA PF6	7.5	2.1	Collidine	DCM	2	0.02	C	Pt	64
TBA PF6	7.5	2.1	Collidine	DCM	2	0.02	C	Ni	58
TBA PF6	7.5	2.1	Collidine	DCM	2	0.02	C	Zn	60
TBA PF6	7.5	2.1	Collidine	DCM	2	0.02	C	SS	65
TBA PF6	7.5	2.1	Collidine	DCM	2	0.02	C	C	59

Table S 9: Optimization of the counter electrode material.

Optimization of the substrate concentration:

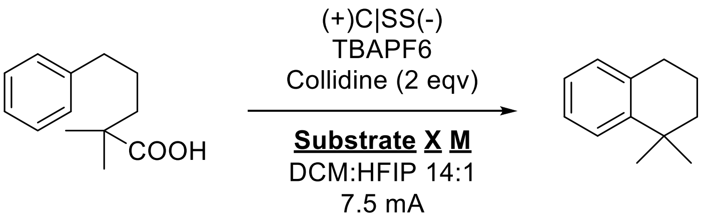

Electrolyte (0.1 M)	Current intensity (mA)	Current quantity (F/mol)	Base (type)	Solvent (X : HFIP 14:1)	Base (eqv)	Substrate concentration (M)	Working electrode	Counter electrode	Yield % (GC/MS)
TBA PF6	7.5	2.1	Collidine	DCM	2	0.01	C	SS	55
TBA PF6	7.5	2.1	Collidine	DCM	2	0.02	C	SS	65
TBA PF6	7.5	2.1	Collidine	DCM	2	0.04	C	SS	65
TBA PF6	7.5	2.1	Collidine	DCM	2	0.1	C	SS	48

Table S 10: Optimization of the substrate concentration.

Final optimized best conditions for batch electrolysis:

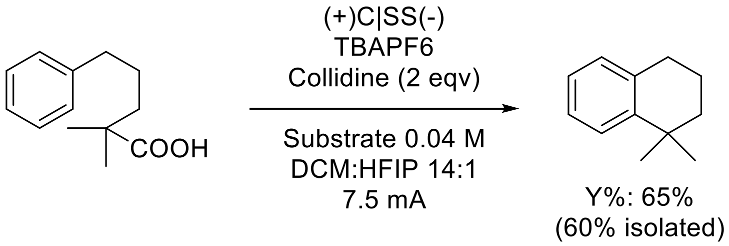

Best electrolyte (0.1 M)	Best current intensity (mA)	Best current quantity (F/mol)	Best base (type)	Best solvent (X : HFIP 14:1)	Bestbase (eqv)	Best substrate concentration (M)	Best working electrode	Best counter electrode	Best yield % (GC/MS)
TBA PF6	7.5	2.1	Collidine	DCM	2	0.02-0.04	C	SS	65 (56% isolated)

Table S 11: Best conditions after the optimization process.

For the isolated yield, the solvent was evaporated under reduced pressure after the reaction was complete and the residue was passed over a silica plug eluted with n-Pentane.

MW: 160.26 g/mol

^{1}H NMR (400 MHz, Chloroform-*d*) δ 7.59 – 7.25 (m, 2H), 7.33 – 7.16 (m, 2H), 2.96 (t, *J* = 6.3 Hz, 2H), 2.11 – 1.89 (m, 2H), 1.86 (dt, *J* = 9.5, 2.6 Hz, 2H), 1.48 (s, 6H).

^{13}C{^{1}H} NMR (101 MHz, Chloroform-*d*) δ 145.8, 136.1, 129.1, 126.7, 125.8, 125.3, 39.4, 33.9, 31.9, 30.8, 29.8, 19.8.

Batch electrolysis of 2-10(b or e or g)

General procedure 2 *optimized conditions for batch electrolysis of* **2-10(b or e or g)**

All reactions were performed in an IKA ElectraSyn 2.0 using Graphite SK-50 as working electrode and stainless steel as counter electrode. Electrodes were purchased from IKA. In a 10 mL IKA ElectraSyn vial, equipped with a stir bar, 1 mmol of $TBAPF_6$, 0.4 mmol (1 eqv) of carboxylic acid **2-10(b or e or g)**, 0.7 mL of HFIP, 0.4 mmol of base (2 eqv) were dissolved in 10 mL of dichloromethane and bubbled with Ar while mixed for 5 minutes, than the electrochemical cell was assembled. The instrument was operated under constant current mode at 7.5 mA, at 400 rpm of stirring. After 2.1 F/mol were passed through, the solvent was removed under reduced pressure and the residue was purified over a silica pad, using n-Pentane as mobile phase.

3b *batch electrolysis*

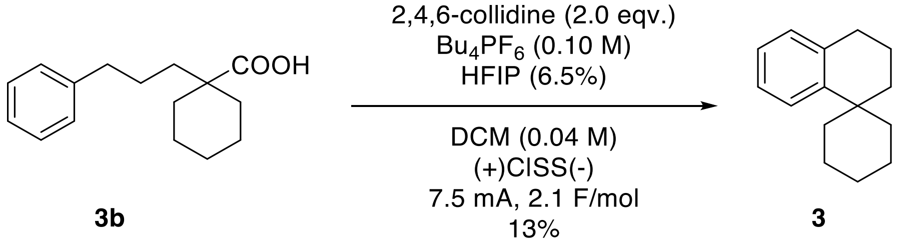

Figure S 40: Electrolysis of 1-(3-phenylpropyl)cyclohexane-1-carboxylic acid in optimal conditions.

3b was electrolyzed following **General procedure 2**. **3** was obtained as a clear oil in 13% isolated yield.

MW: 200.32 g/mol

^{1}H NMR (400 MHz, Chloroform-*d*) δ 7.14 – 7.06 (m, 2H), 7.02 – 6.95 (m, 2H), 2.68 (t, *J* = 6.2 Hz, 2H), 1.75 (dt, *J* = 5.8, 3.5 Hz, 2H), 1.72 – 1.60 (m, 6H), 1.48 (dddd, *J* = 25.2, 12.9, 5.5, 2.7 Hz, 6H)

^{13}C NMR (101 MHz, Chloroform-*d*) δ 146.74, 137.22, 129.13, 126.91, 125.87, 125.24, 38.90, 37.16, 31.15, 31.13, 26.34, 22.15, 19.31.

4b *batch electrolysis*

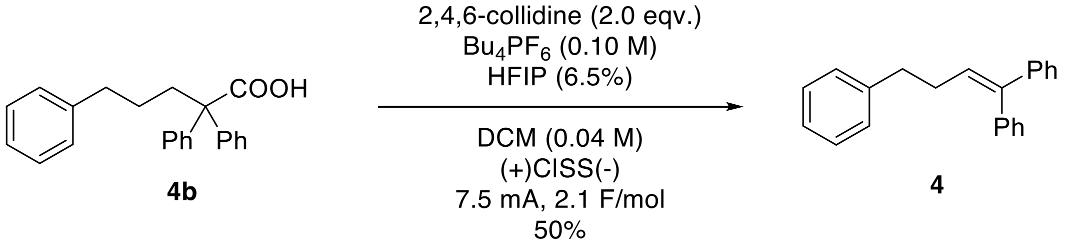

Figure S 41: Electrolysis of 2,2,5-triphenylpentanoic acid in optimal conditions.

4b has been electrolyzed following **General procedure 2**. **4** was obtained as a clear oil in 50% isolated yield. ^{1}H NMR analysis was consistent with results reported by Huang, Hanchu; et al.

^{1}H NMR (400 MHz, Chloroform-*d*) δ 7.28 – 7.02 (m, 15H), 6.03 (t, *J* = 7.4 Hz, 1H), 2.66 (dd, *J* = 8.6, 6.7 Hz, 2H), 2.39 – 2.33 (m, 2H).

5e *batch electrolysis*

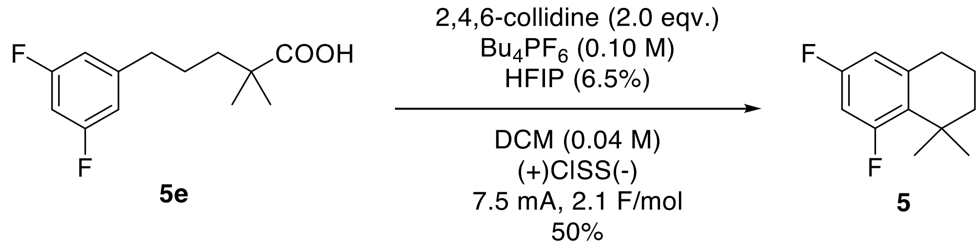

Figure S 42: Electrolysis of 5-(3,5-difluorophenyl)-2,2-dimethylpentanoic acid in optimal conditions.

5e has been electrolyzed following **General procedure 2**. **5** was obtained as a clear oil in 50% isolated yield

^{1}H NMR (400 MHz, Chloroform-*d*) δ 6.56 – 6.44 (m, 2H), 2.64 (t, J = 6.2 Hz, 2H), 1.66 (dtd, J = 8.9, 5.2, 4.4, 2.6 Hz, 2H), 1.57 (dt, J = 8.9, 2.6 Hz, 2H), 1.27 (d, J = 1.7 Hz, 6H).

^{13}C{^{1}H} NMR (101 MHz, Chloroform-*d*) δ 163.7, 161.6, 161.5, 161.2, 159.0, 142.7 – 139.85 (m), 128.3 (d, J = 8.5 Hz), 111.0 (dd, J = 19.7, 3.1 Hz), 102.0 (dd, J = 28.7, 24.7 Hz), 41.2, 33.1 (d, J = 2.3 Hz), 32.3 – 30.6 (m), 29.0 (d, J = 5.1 Hz), 19.4.

6e *batch electrolysis*

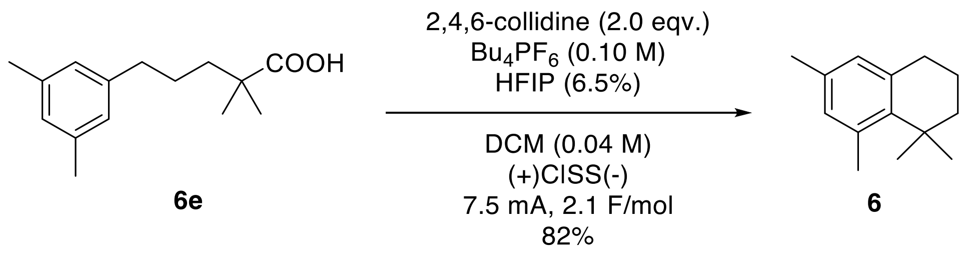

Figure S 43: Electrolysis of 5-(3,5-dimethylphenyl)-2,2-dimethylpentanoic acid in optimal conditions.

6e has been electrolyzed following **General procedure 2**. **6** was obtained as a clear oil in 82% isolated yield

^{1}H NMR (400 MHz, Chloroform-*d*) δ 6.84 – 6.75 (m, 2H), 2.79 (q, *J* = 6.3 Hz, 2H), 2.51 (s, 3H), 2.26 (s, 3H), 1.42 (s, 6H).

^{13}C{^{1}H} NMR (101 MHz, Chloroform-*d*) δ 140.5, 137.3, 137.0, 134.6, 131.5, 128.4, 43.9, 34.5, 32.5, 29.3, 29.2, 23.5, 20.5, 19.6.

7e *batch electrolysis*

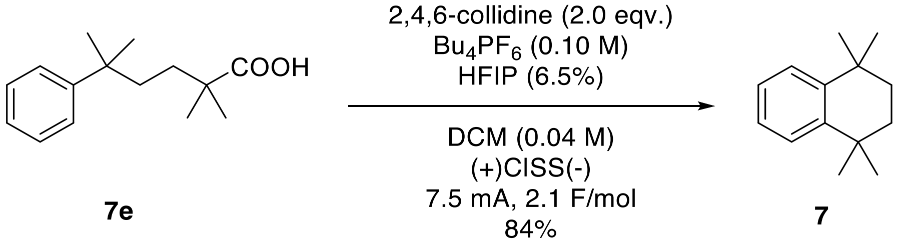

7e **7**

Figure S 44: Electrolysis of 2,2,5-trimethyl-5-phenylhexanoic acid in optimal conditions.

7e has been electrolyzed following **General procedure 2**. **7** was obtained as a clear oil in 84% isolated yield

^{1}H NMR (400 MHz, Chloroform-*d*) δ 7.23 (dq, *J* = 7.0, 3.5 Hz, 2H), 7.04 (dd, *J* = 5.9, 3.4 Hz, 2H), 1.21 (s, 12H).

^{13}C{^{1}H} NMR (101 MHz, Chloroform-*d*) δ 144.8, 126.5, 125.5, 35.2, 34.2, 31.9.

8g *batch electrolysis*

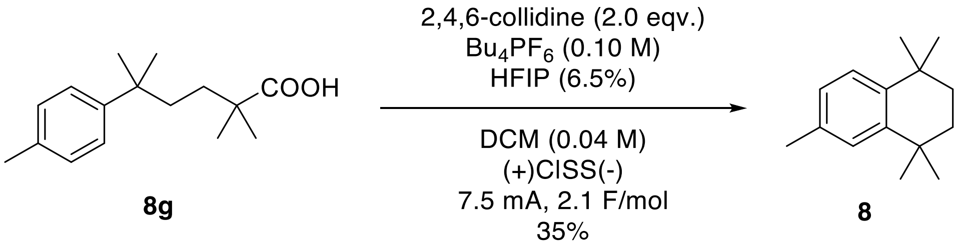

Figure S 45: Electrolysis of 2,2,5-trimethyl-5-phenylhexanoic acid in optimal conditions.

8g has been electrolyzed following **General procedure 2**. **8** was obtained as a clear oil in 35% isolated yield

^{1}H NMR analysis was consistent to the one reported by Ledneczki, Istvan; et al.

9e *batch electrolysis*

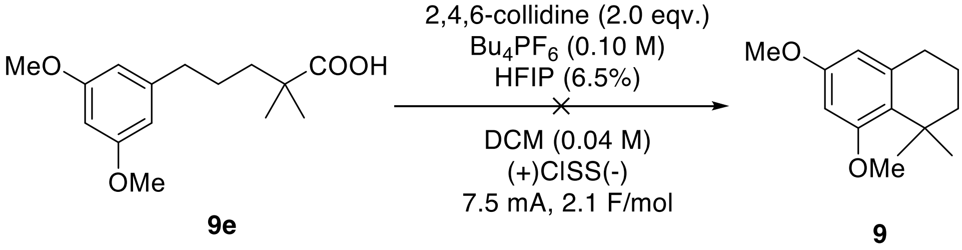

Figure S 46: Batch electrolysis of 5-(3,5-dimethoxyphenyl)-2,2-dimethylpentanoic acid

9e has been electrolyzed following **General procedure 2**. **9** was not found in the reaction mixture.

10e *batch electrolysis*

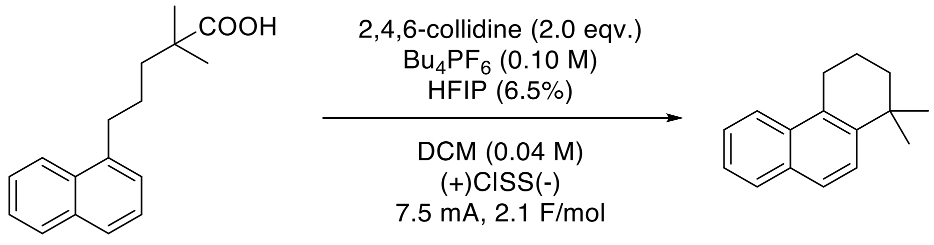

10e **10**

Figure S 47: Batch electrolysis of methyl 2,2-dimethyl-5-(naphthalen-1-yl)pentanoic acid

10e has been electrolyzed following **General procedure 2**. **10** was obtained as a clear oil in 40% isolated yield.

^{1}H NMR (400 MHz, Chloroform-*d*) δ 7.92 (dq, *J* = 8.4, 0.9 Hz, 1H), 7.70 (dd, *J* = 8.4, 1.3 Hz, 1H), 7.59 (d, *J* = 8.7 Hz, 1H), 7.48 – 7.32 (m, 3H), 3.06 (t, *J* = 6.4 Hz, 2H), 1.96 – 1.85 (m, 2H), 1.72 – 1.65 (m, 2H), 1.29 (s, 6H).

Flow experiments

Every flow experiment has been performed using a hand-made electrochemical flow cell.

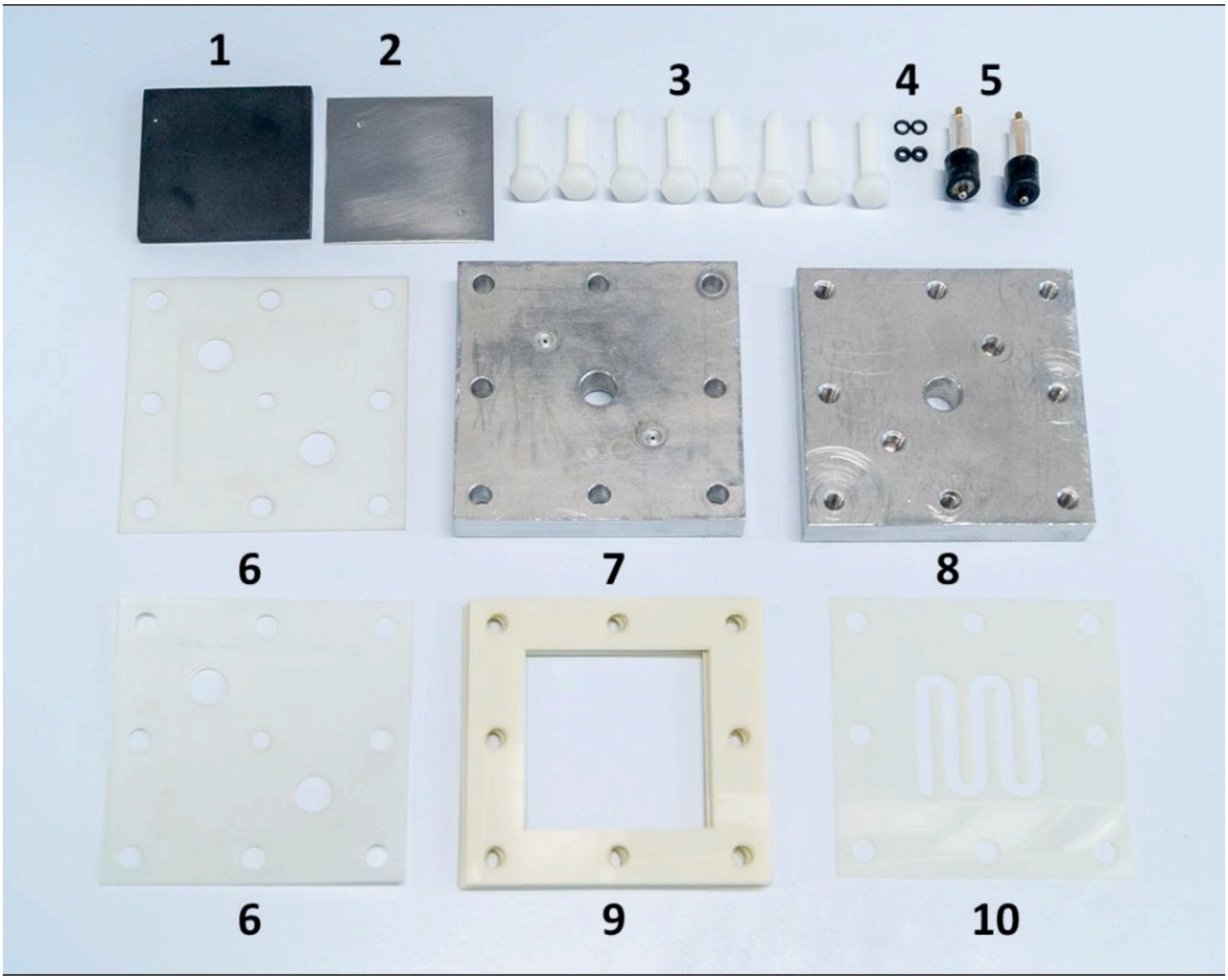

Figure S 48: Components of the flow cell utilized in this work. (11)

Components of the flow electrolysis cell utilized in this work. **1**: Graphite anode (IG-63, GTD Graphit Technologie GmbH, 50 × 50 × 3 mm). **2**: Stainless steel cathode (Stainless Steel - AISI 316L, Fe/Cr 18%/Ni 10%/Mo 3%, foil, 50 × 50 × 0.1 mm) incorporating two 1 mm holes. **3**: M6 bolts (polyamide, 30 mm, DIN 933). **4**: O-rings: 5 mm o.d./2 mm i.d., EPDM (internal); 5 mm o.d., 3 mm i.d., EPDM (external for fitting connections). **5**: current collectors (pogo-pins 2.6 mm diameter, 35 mm length, PTR 1040-D-1.5N-NI-2.4) in a 10 mm o.d. tubing adaptor. **6**: isolation layer (laser-cut Mylar foil, 0.3 mm thickness). **7**: end

plate 1 (no M6 threads, inside view). **8**: end plate 2 (with M6 threads, outer view). **9**: alignment gaskets (laser-cut Mylar foil, 0.3 mm thickness). **10**: electrode separator/reaction channel (laser-cut Mylar foil) (11).

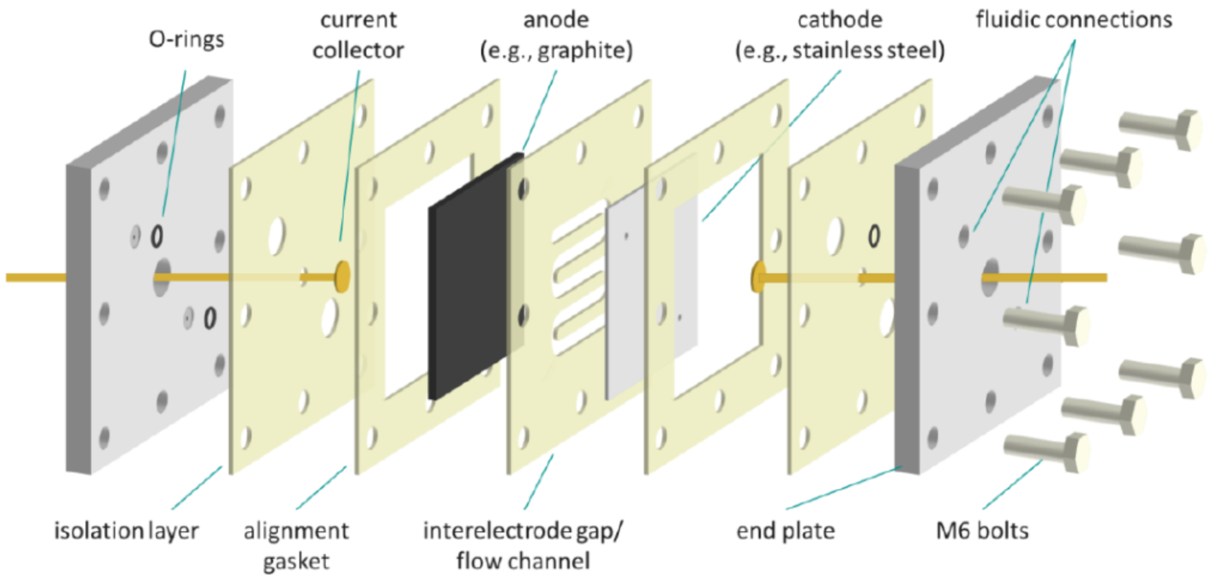

Figure S 49: Scheme of the exploded flow reactor utilized in this work. (11)

Flow recirculation optimization on 2b

General procedure 3 for recirculation flow optimization electrolysis of **2b**:

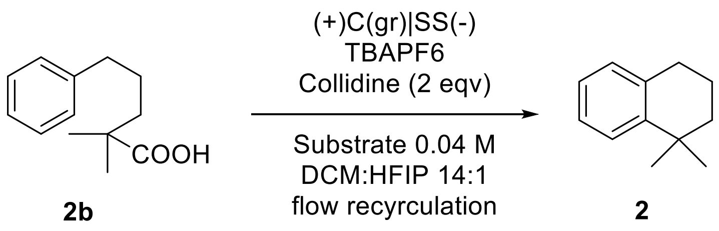

A 20 ml vial equipped with a magnetic stir bar was charged with 0.4 mmol of **2b**, 1 mmol of $TBAPF_6$, 10 ml of DCM, 0.8 mmol of 2,4,6-collidine and 0.7 mL of HFIP. For this purpose, a peristaltic pump was utilized (5 mL/min flow rate). The reactor was operated under

constant current mode using a PeakTech 6225 A power supply until the desired amount of charge was passed to the reagent solution. Then, the reactor inlet was removed from the reaction mixture reservoir allowing air to enter the channel and flush all the remaining reaction mixture to the vessel. Parameters like current (mA) and charge (F/mol) have been changed for the optimization of the process. Aliquots (100 μL) of the reaction mixture were collected from the reservoir during cell operation to monitor the reaction progress by HPLC.

HPLC has been calibrated using anisole as an internal standard.

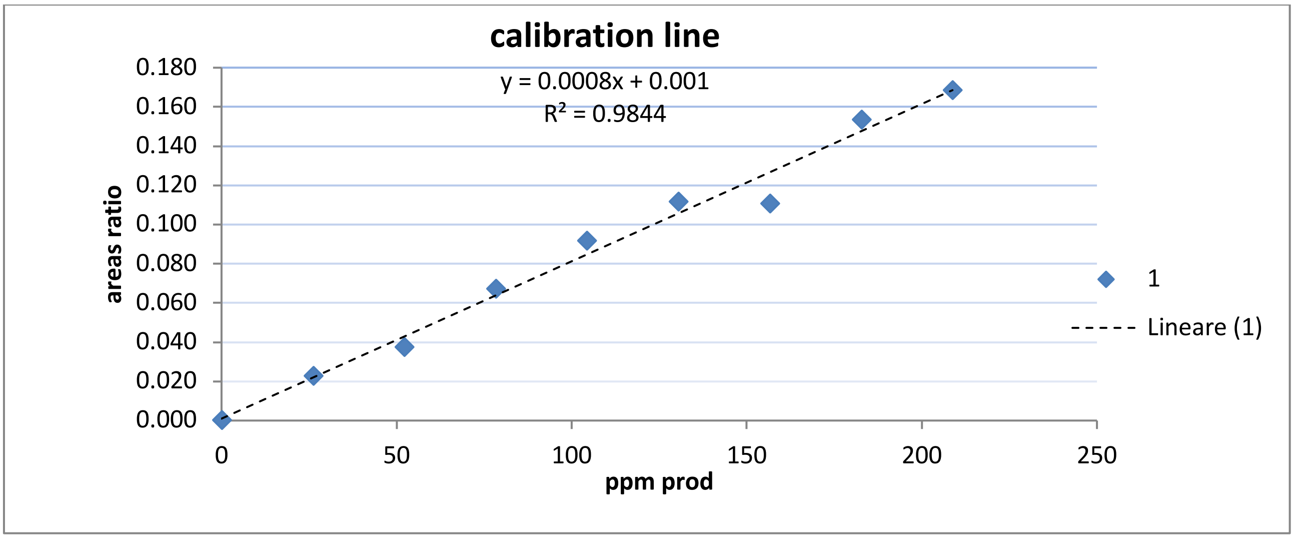

Figure S 50: Calibration line of HPLC

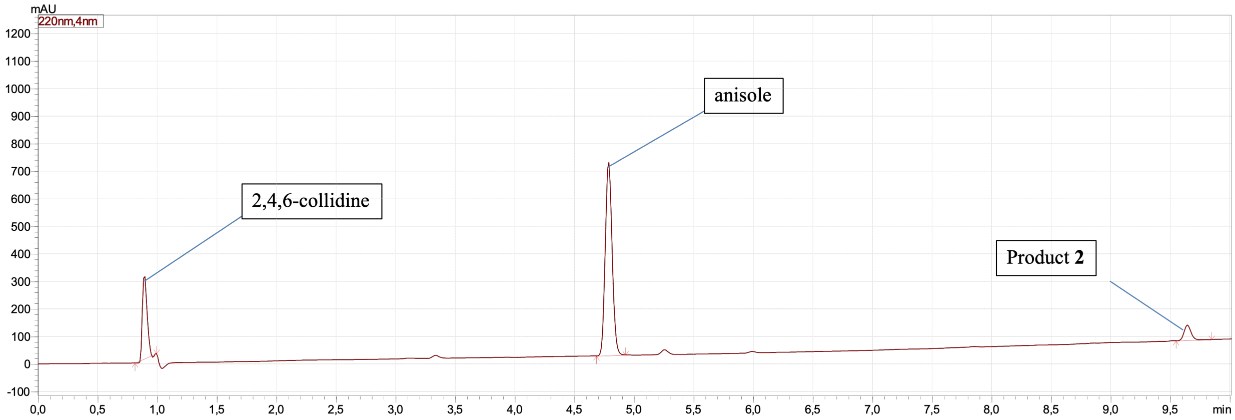

Figure S 51: Typical HPLC chromatogram of an optimization reaction with the internal standard.

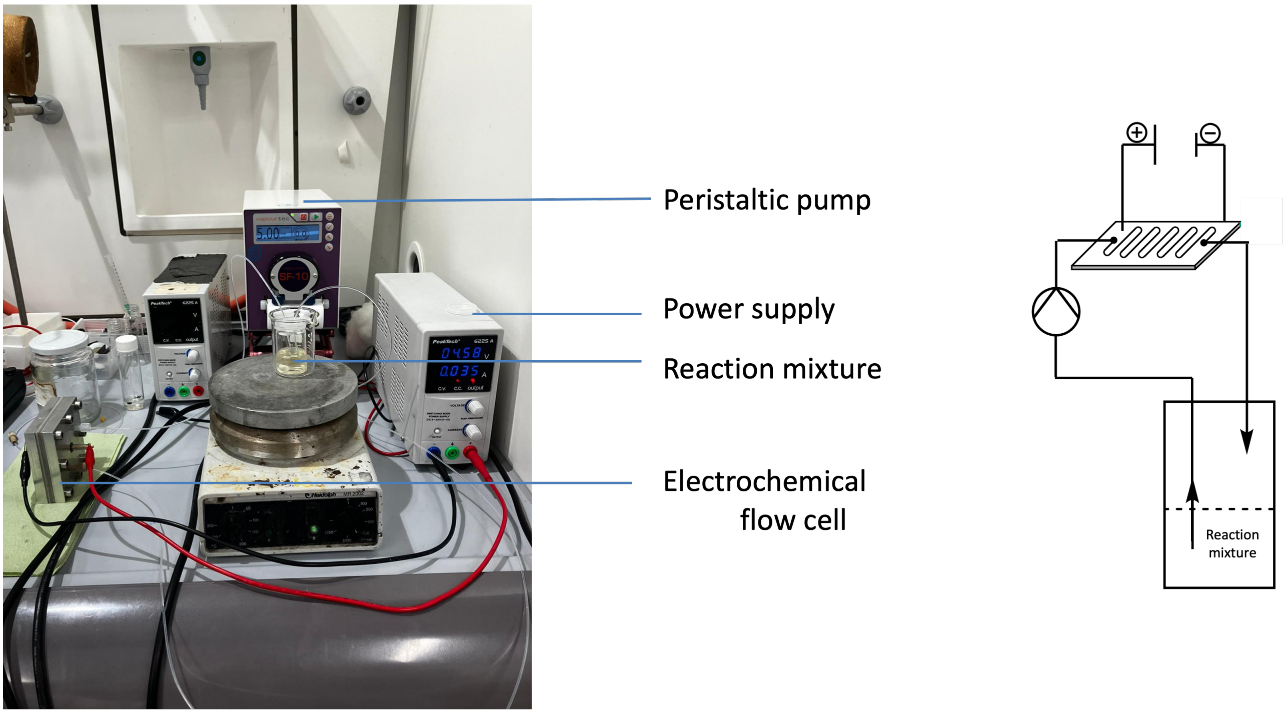

Figure S 52: Real image of the recirculation setup (left); schematic image of the recirculation setup (right).

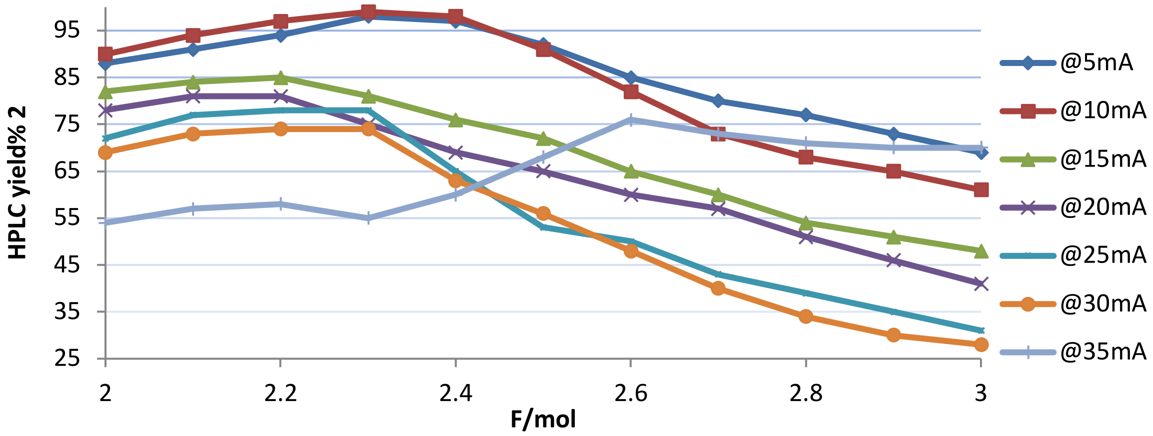

Figure S 53: Trend of the HPLC yields against F/mol applied for each current.

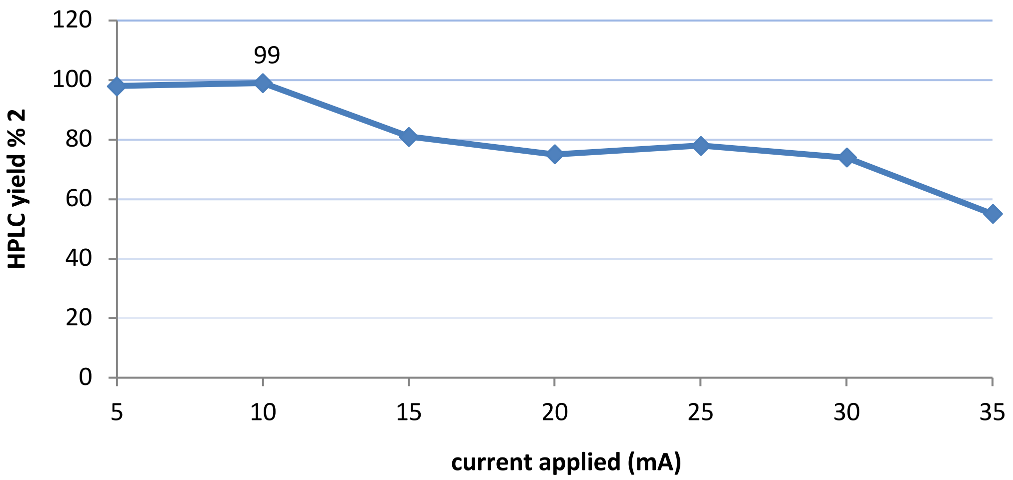

Figure S 54: Trend of the yields against currents applied at 2.3 F/mol

Flow Recirculation Electrolysis of 2-10(b or e or g)

General procedure 4 for flow recirculation electrolysis of **2-10(b or e or g):**

A 20 ml vial equipped with a magnetic stir bar was charged with 0.4 mmol of carboxylic acid, 1 mmol of $TBAPF_6$, 10 ml of DCM, 0.8 mmol of 2,4,6-collidine and 0.7 mL of HFIP. For this purpose, a peristaltic pump was utilized (5 mL/min flow rate). The reactor was operated under constant current mode using a PeakTech 6225 A power supply at 10 mA until 2.3 F/mol were passed to the reagent solution. Then, the reactor inlet was removed from the reaction mixture reservoir allowing air to enter the channel and flush all the remaining reaction mixture to the vessel. After 2.3 F/mol were passed, the solvent was removed under vacuum and the residue was filtered over a silica pad eluted with n-pentane to afford the product.

Flow recirculation electrolysis of **3b**:

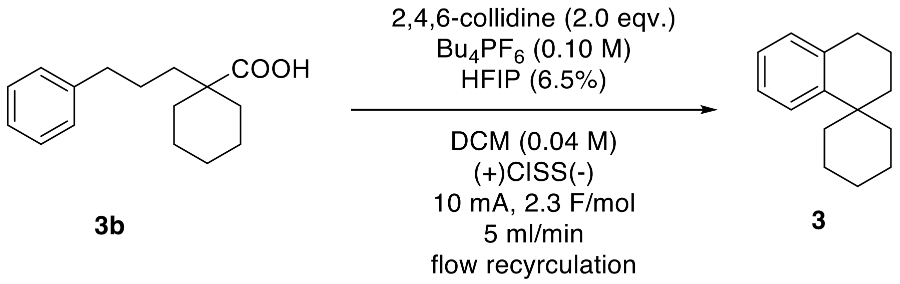

Figure S 55: Flow recirculation electrolysis of 1-(3-phenylpropyl)cyclohexane-1-carboxylic acid in optimized flow conditions

The product **3** has been synthesized and purified according to **General procedure 4**. Y% = 61%

Flow recirculation electrolysis of **4b**:

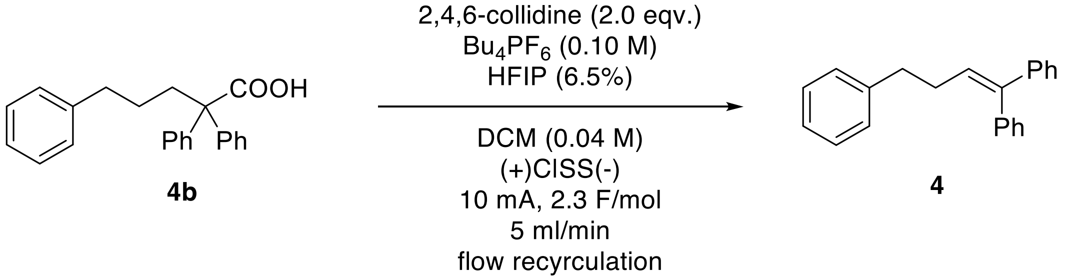

Figure S 56: Flow recirculation electrolysis of 2,2,5-triphenylpentanoic acid in optimized flow conditions.

The product **4** has been synthesized and purified according to **General procedure 4**. Y% = 87%.

Flow recirculation electrolysis of **5e**:

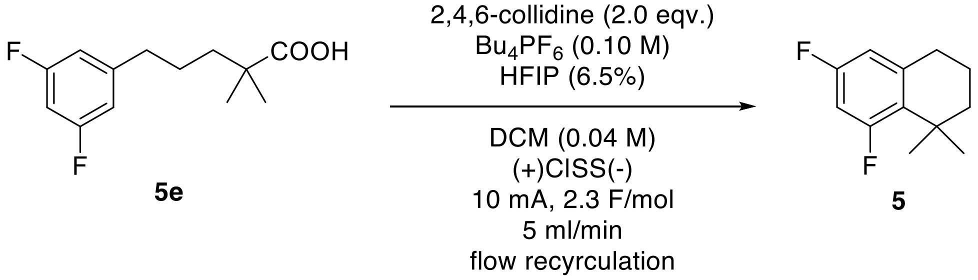

Figure S 57: Flow recirculation electrolysis of 5-(3,5-difluorophenyl)-2,2-dimethylpentanoic acid in optimized flow conditions.

The product **5** has been synthesized and purified according to **General procedure 4**. Y% = 80%

Flow recirculation electrolysis of **6e**:

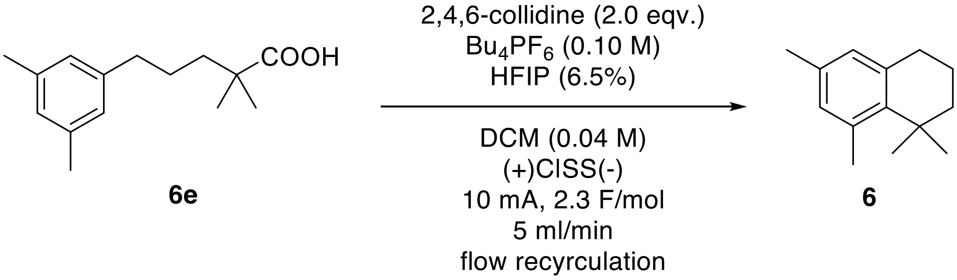

Figure S 58: Flow recirculation electrolysis of 5-(3,5-dimethylphenyl)-2,2-dimethylpentanoic acid in optimized flow conditions.

The product **6** has been synthesized and purified according to **General procedure 4**. Y% = 94%.

Flow recirculation electrolysis of **7e**:

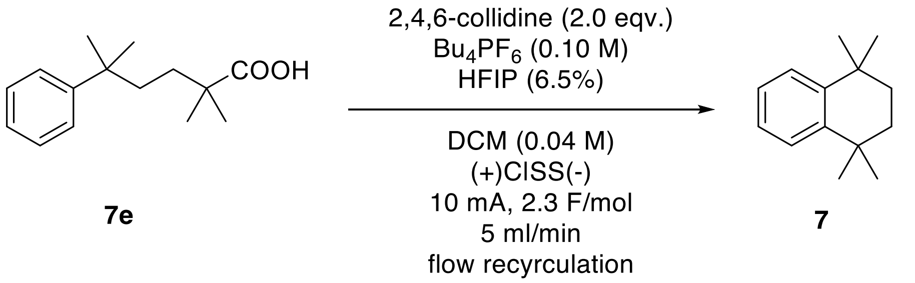

Figure S 59: Flow recirculation electrolysis of 2,2,5-trimethyl-5-phenylhexanoic acid in optimized flow conditions.

The product **7** has been synthesized and purified according to **General procedure 4**. Y% = 98%.

Flow recirculation electrolysis of **8g**:

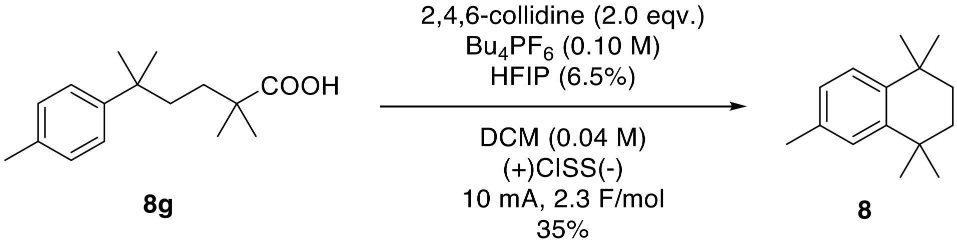

Figure S 60: Flow electrolysis of 2,2,5-trimethyl-5-(p-tolyl)hexanoic acid in optimized flow conditions.

The product **8** has been synthesized and purified according to **General procedure 4**. Y% = 75%.

Flow recirculation electrolysis of **9e**:

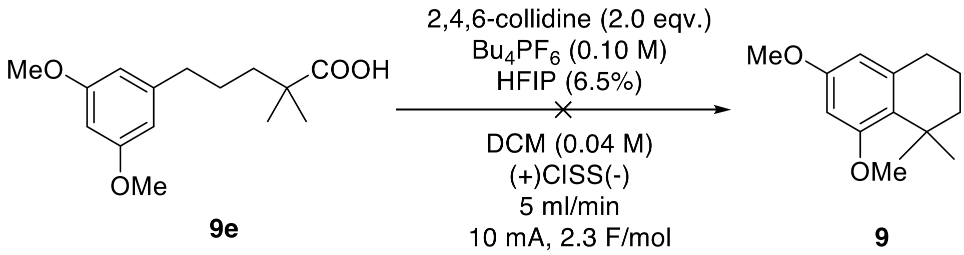

Figure S 61: Flow recirculation electrolysis of 5-(3,5-dimethoxyphenyl)-2,2-dimethylpentanoic acid

The product **P7** has been synthesized and purified according to **General procedure 4**. Product **9** has not been detected.

Flow recirculation electrolysis of **10e**:

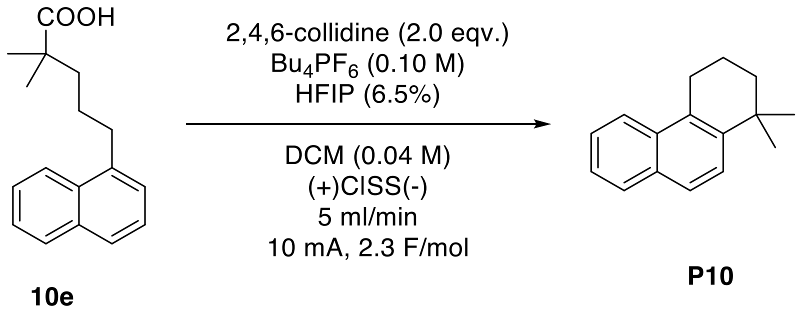

Figure S 62: Flow recirculation electrolysis of 1,1-dimethyl-1,2,3,4-tetrahydrophenanthrene

The product **10** has been synthesized and purified according to **General procedure 4**. Y% = 98%

Flow single pass optimization on 2b

General procedure 6 *for flow single-pass optimization electrolysis of* **2b**

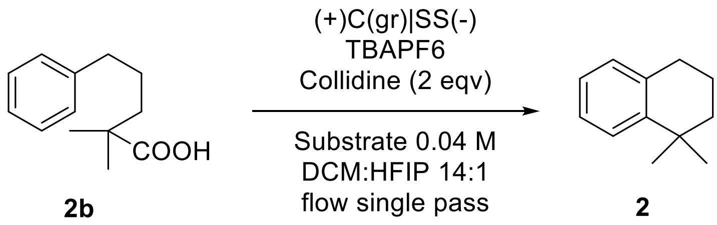

A 250 ml sealed bottle equipped with a magnetic stir bar was charged with 4 mmol of **2b**, 10 mmol of $TBAPF_6$, 100 ml of DCM, 8 mmol of 2,4,6-collidine and 7 mL of HFIP. For this purpose, a peristaltic pump was utilized. The reactor was operated under constant current mode using a PeakTech 6225 A power supply. The formed solution was pumped through a flow electrolysis cell equipped with a graphite anode and a stainless steel cathode and an interelectrode gap separator of 0.9 mm thickness containing a channel (6.4 cm^2 surface, 570 µL volume). The output tube from the electrochemical cell has been connected into HPLC vials. Parameters like current and flow rate has been changed to optimize the process. 100 uL for each run has been collected to monitor the yield via HPLC.

HPLC has been calibrated using anisole as an internal standard.

F/mol	mL/min	Current (mA)	Yield (%)	F/mol	mL/min	Current (mA)	Yield (%)
2.3	0.0541	5	97.29	2.3	0.1622	15	82.42
2.4	0.0518	5	90.15	2.4	0.1555	15	80.86
2.5	0.0497	5	85.78	2.5	0.1492	15	78.68
2.6	0.0478	5	86.60	2.6	0.1435	15	69.64
2.7	0.0461	5	80.12	2.7	0.1382	15	66.07
2.8	0.0444	5	73.29	2.8	0.1333	15	62.65
2.9	0.0429	5	72.80	2.9	0.1287	15	60.45
2.3	0.1081	10	99.57	2.3	0.2163	20	56.45
2.4	0.1036	10	88.00	2.4	0.2073	20	54.15
2.5	0.0995	10	86.02	2.5	0.1990	20	51.31
2.6	0.0957	10	86.06	2.6	0.1913	20	46.82
2.7	0.0921	10	79.65	2.7	0.1843	20	45.91
2.8	0.0888	10	74.60	2.8	0.1777	20	42.35
2.9	0.0858	10	71.56	2.9	0.1715	20	40.32

F/mol	mL/min	Current (mA)	Yield (%)	F/mol	mL/min	Current (mA)	Yield (%)
2.3	0.2704	25	51.49	2.3	0.3785	35	21.72
2.4	0.2591	25	39.61	2.4	0.3627	35	19.07
2.5	0.2487	25	38.76	2.5	0.3482	35	18.03
2.6	0.2392	25	38.43	2.6	0.3348	35	14.24
2.7	0.2303	25	37.29	2.7	0.3224	35	12.69
2.8	0.2221	25	36.52	2.8	0.3109	35	11.38
2.9	0.2144	25	35.80	2.9	0.3002	35	10.03
2.3	0.3244	30	40.86	2.3	0.4326	40	15.47
2.4	0.3109	30	39.38	2.4	0.4146	40	14.29
2.5	0.2985	30	39.67	2.5	0.3980	40	12.73
2.6	0.2870	30	37.11	2.6	0.3827	40	9.97
2.7	0.2764	30	36.03	2.7	0.3685	40	8.25
2.8	0.2665	30	30.54	2.8	0.3553	40	7.51
2.9	0.2573	30	29.73	2.9	0.3431	40	6.69

Table S 12: Detailed optimization data for the single-pass continuous flow electrolysis of **2b.**

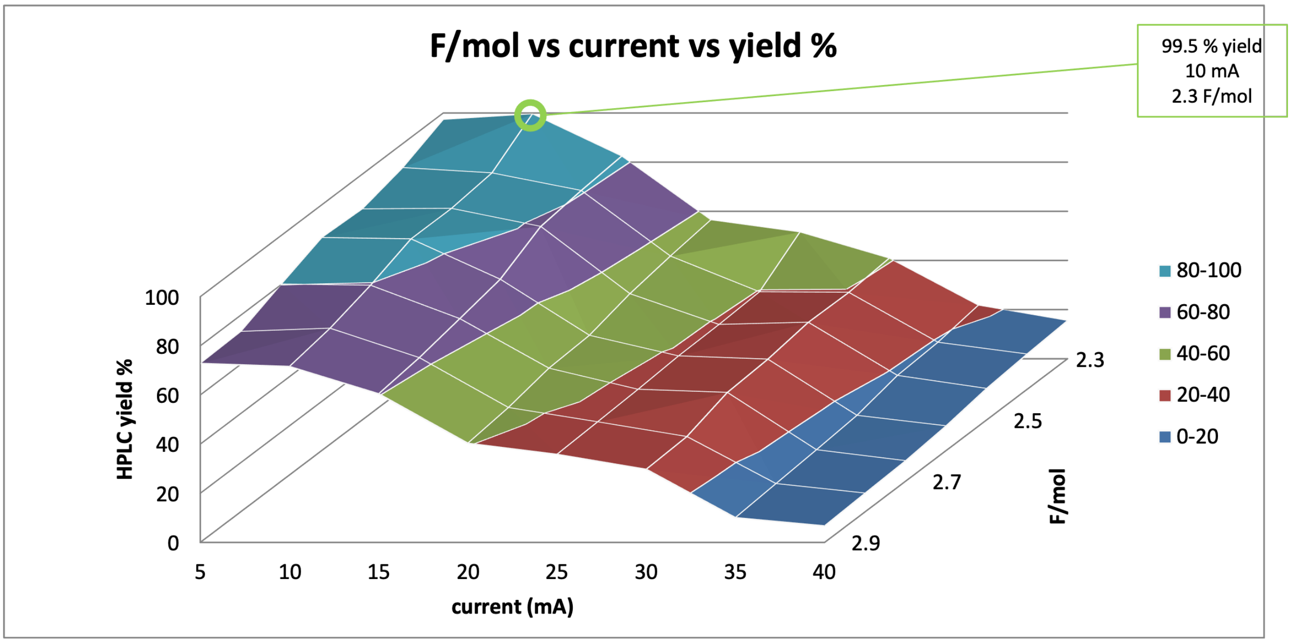

Figure S 63: Trend of HPLC yields against current applied and F/mol.

Flow single pass long run electrolysis of 2b

A 24 hours long run has been performed for yield stability monitoring.

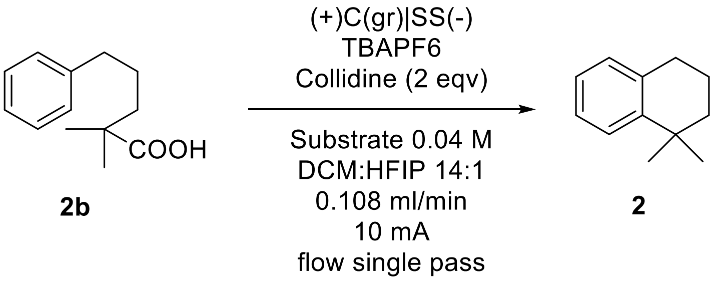

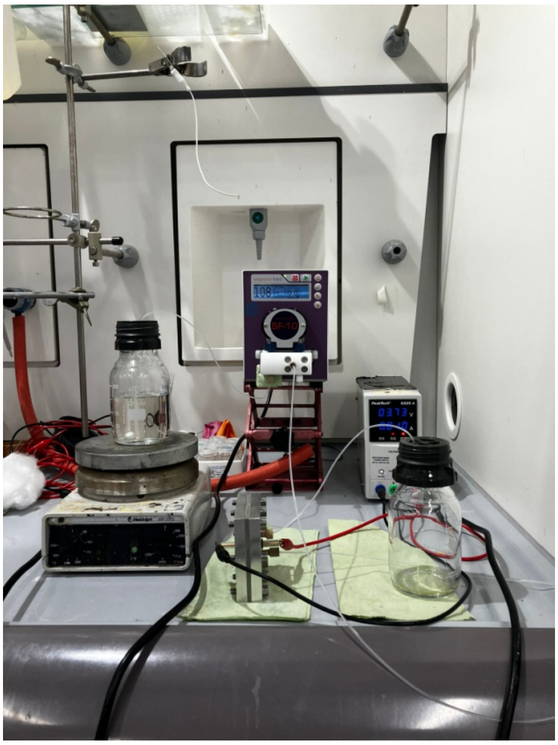

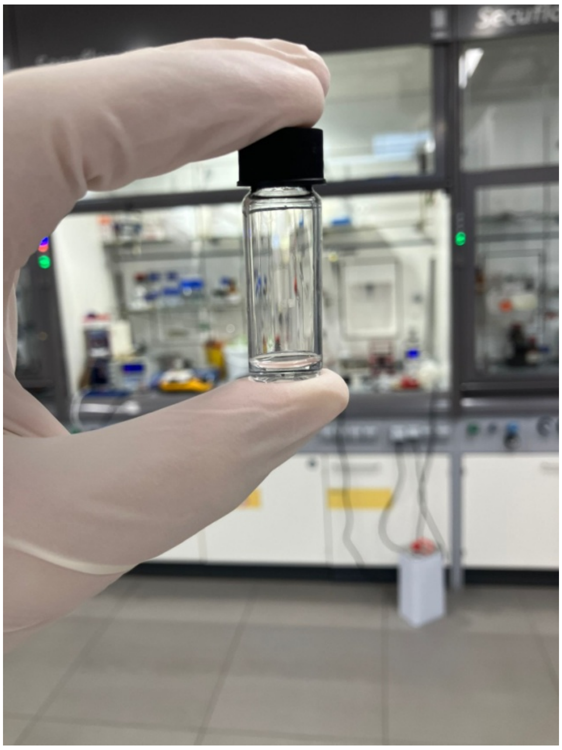

Figure S 64: Picture of the experimental setup of single pass long run (left).
Figure S 65: product obtained from the long run experiment (right).

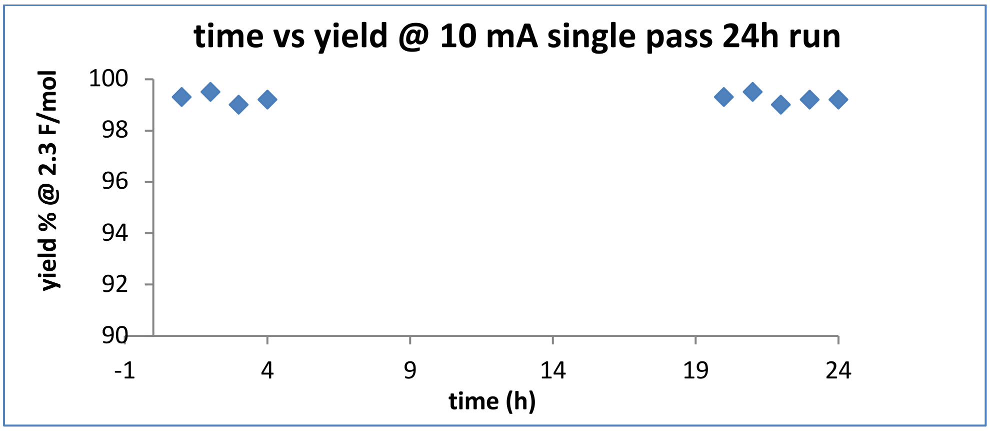

Figure S 66: Trend of the HPLC yields during the long run.

Mechanistic studies

Procedure for radical trapping with TEMPO

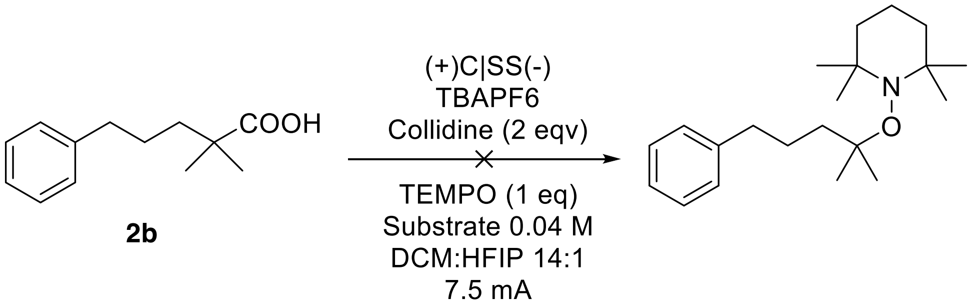

2b

The reaction was performed according to General procedure 2, adding 1 eq of TEMPO at the reaction. The reaction mixture was then checked via GC-MS and LC-MS and the adduct with TEMPO was not found. The cyclized product **2** has been detected instead.

Procedure for ion trapping with MeOH

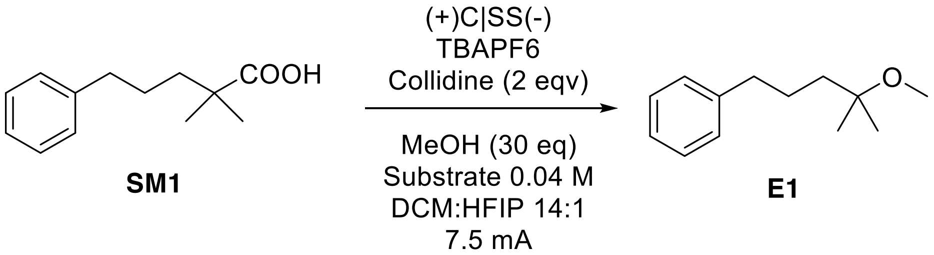

SM1 **E1**

The reaction was performed according to **General procedure 2**, adding 30 eq. of MeOH at the reaction. The reaction mixture was then checked via GC-MS and product **E1** was found as reported in the chromatogram and mass spectra (**Figure S 61-62**).

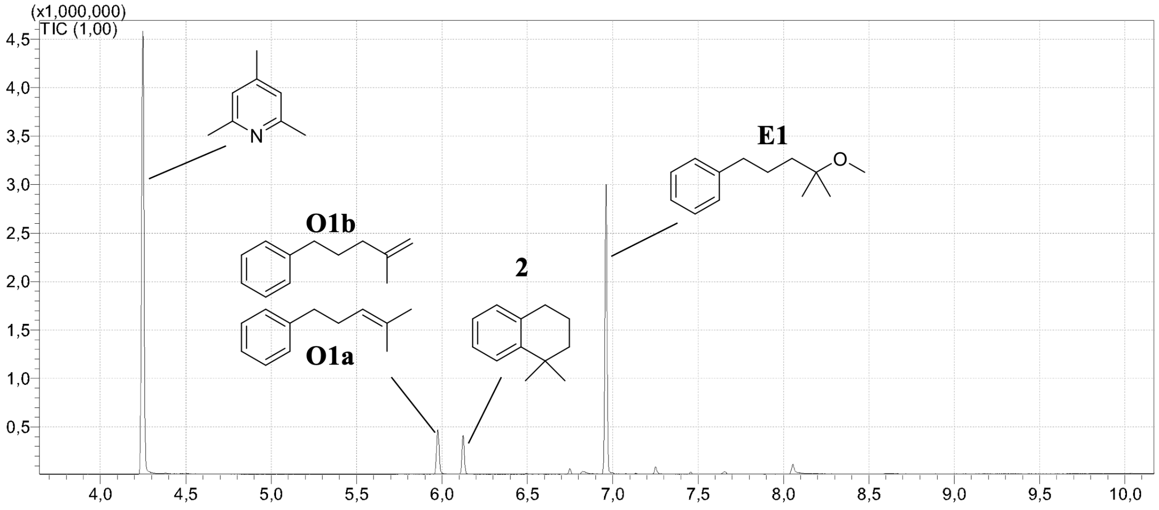

Figure S 67: Gas chromatogram of the ion trapping experiment.

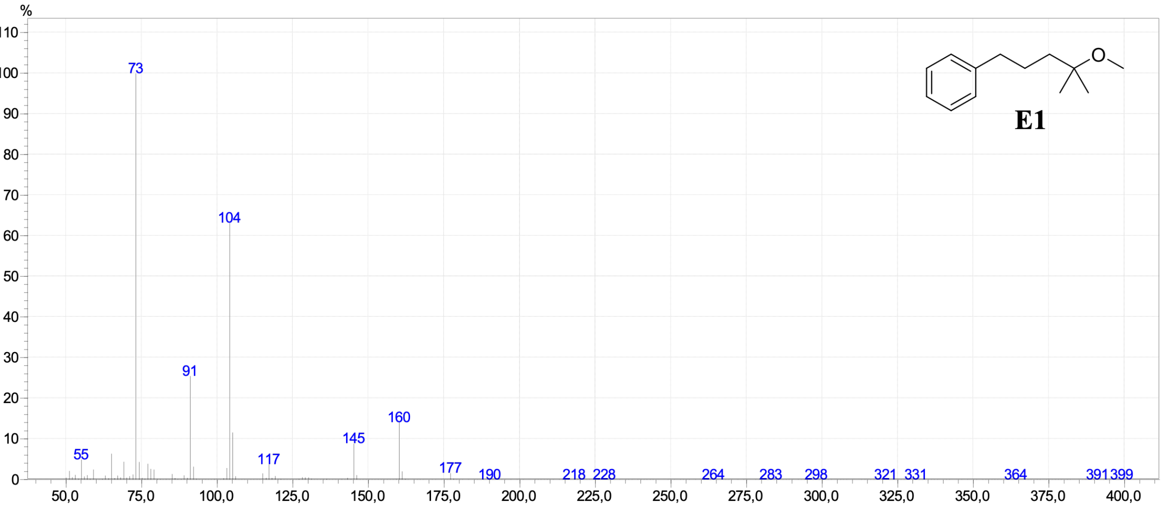

Figure S 68: EI mass spectra of the ether E1 product.

Computational Details

The structure for **B** was prepared and subjected to conformational sampling by using the opensource *CREST* tool associated with the *x*TB software, by specifying the solvents involved in the reaction as well as possible charges already present in the starting materials.(14) This software employs the semiempirical extended tight binding approach known as *x*TB (GFN2-*x*TB) for its theoretical framework, which strikes a balance in sampling a wide range of chemical space. However, its accuracy in calculating thermochemical properties is limited, which necessitates conducting supplementary higher-level calculations.(14) Indeed, with the so obtained conformer structures, we operated a selection by excluding duplicates looking directly at RMSD of the coordinates of the atoms in a spatial region of diameter 1.0Å.

The selected geometries where then subjected to low level of theory calculations using Gaussian 16 software tool and a very simplified level of theory, consisting of B3LYP functional and 6-31g(d,p) as basis set for all the atoms.(15),(16),(17) During these initial calculations the role of dichloromethane solvent was included using the SMD solvation model.(18) The resulting list of optimized conformations of **B** in solvent was then sorted in terms of energies and the most stable one was selected as most preferred conformation.

All structures involved in the next step for the mechanism investigation were optimized using the Gaussian 16 program package.(15) Soon we encountered problems in simulating the elimination step and we decided to use it as a benchmark to identify the best level of theory capable to describe the transition state that links the **B** to the elimination product. In the Table S13 below are reported the level of theory tested and the corresponding descriptive results.

Entry	Functional	Basis set	Result description
**1**	B3LYP	6-31G(d,p)	Optimizing **B** it spontaneously produces the eliminated product without defining a TS
**2**	B3LYP	6-31+G(d,p)	Optimizing **B** produces a structure with elimination TS fashion, but the TS optimization failed producing directly the final eliminated product
**3**	B3LYP-GD3BJ	6-31+G(d,p)	Addition of explicit dispersions during **B** optimization spontaneously produces the eliminated product without defining a TS
**4**	M06-2X	6-31+G(d,p)	Optimizing **B** produced a stable structure, which was then subjected to TS-search succeding in locating and optimizing it

Table S 13: Benchmark for the elimination reaction step and for the correct combination choice for functional and basis set to describe the reaction profile.

Given the reasonable existence of the elimination transition state, we used the combination of functional and basis set capable in simulating it and which constitutes the best compromise between computational cost and accuracy. We thus selected the M06-2X hybrid functional, which demonstrated to be ideal for thermochemistry and thermochemical kinetics studies, by taking in count also noncovalent interactions.(19) The basis set was set on 6-31+g(d,p) for all the atoms,(17),(20) while the effect of the dichloromethane as a solvent was again simulated by SMD solvation model.(18) Frequency calculations to confirm the effective minimum or transition-state (TS) nature of the optimized system, was performed on each structure, as well as intrinsic reaction coordinate (IRC) calculations were performed to confirm the continuity of the reaction profile from TSs toward reactants, intermediates, or products.(21)

The final reported energies were then thermally corrected to the free energy using a higher level of theory through single point calculations (SP). With the optimized structures we used the ωB97XD functional, which already includes a version of Grimme's D2 dispersion model,(22) and the valence triple-zeta polarization basis set def2-TZVP for all the atoms,(23) during the SP calculation. Again, we maintained the same SMD model for the dichloromethane solvent.(18)

^{1}H and ^{13}C NMR spectra

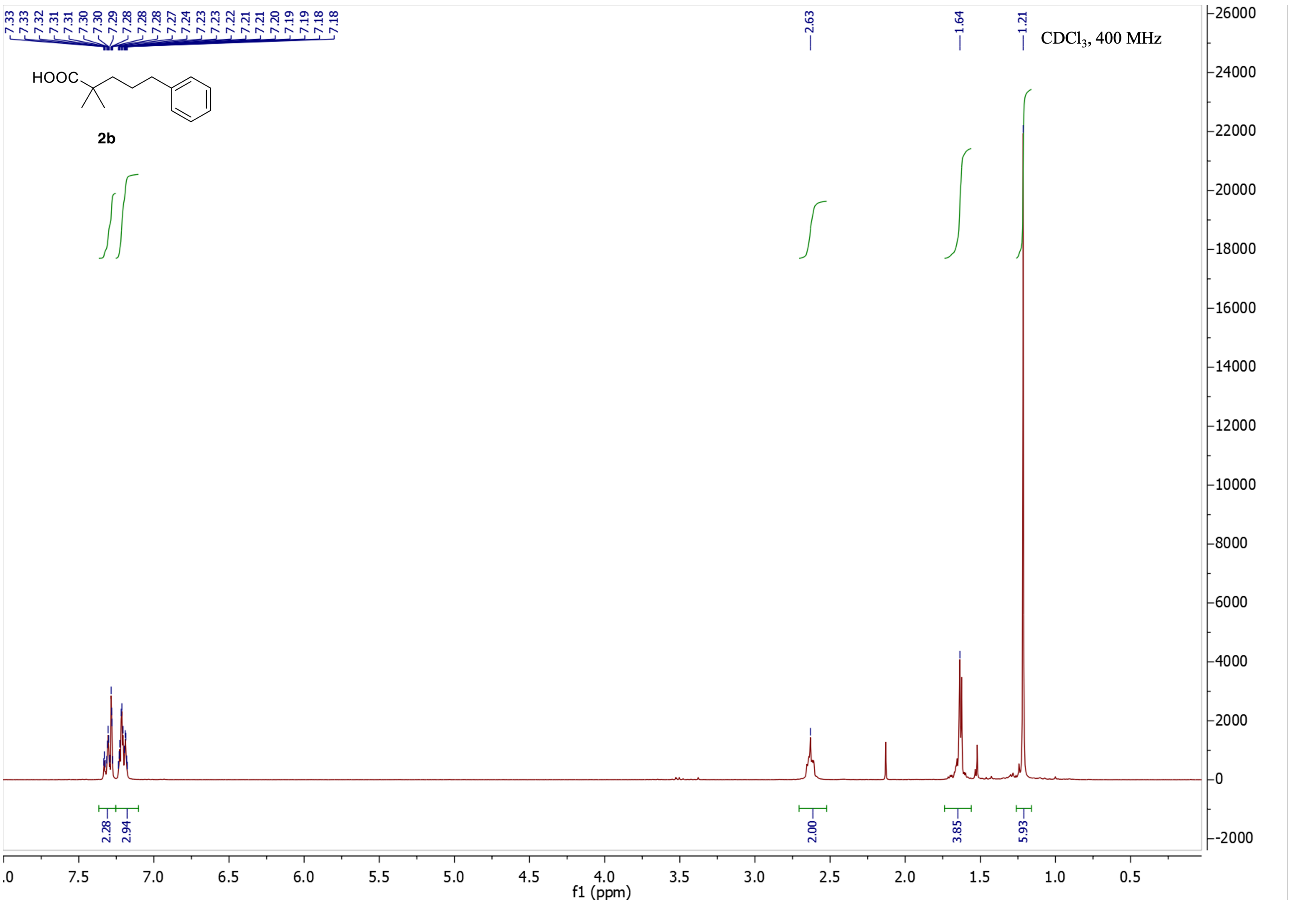

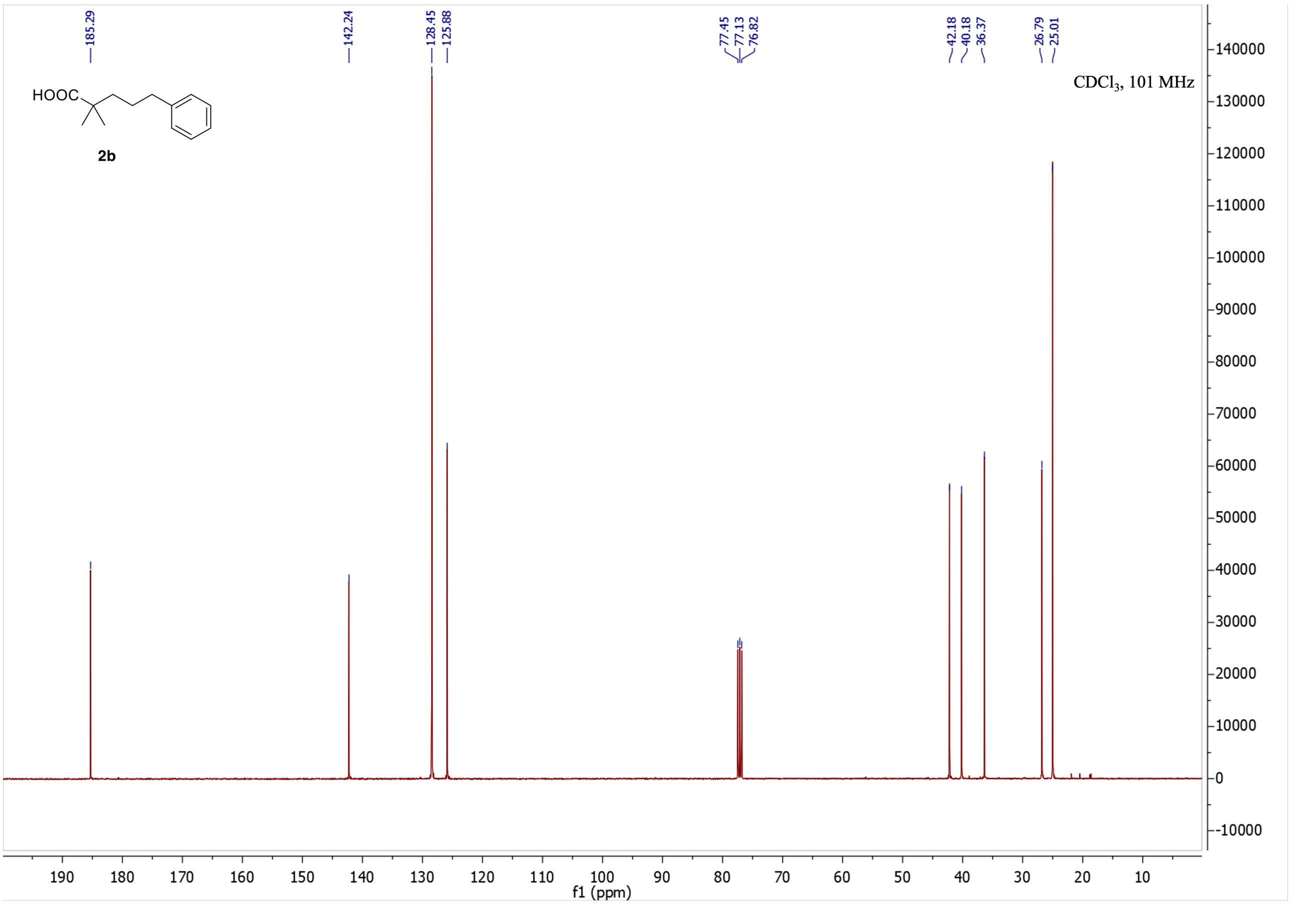

185.29
142.24
128.45
125.88
77.45
77.13
76.82
42.18
40.18
36.37
26.79
25.01
HOOC
2b
CDCl3, 101 MHz
140000
130000
120000
110000
100000
90000
80000
70000
60000
50000
40000
30000
20000
10000
0
-10000
190
180
170
160
150
140
130
120
110
100
90
80
70
60
50
40
30
20
10
f1 (ppm)

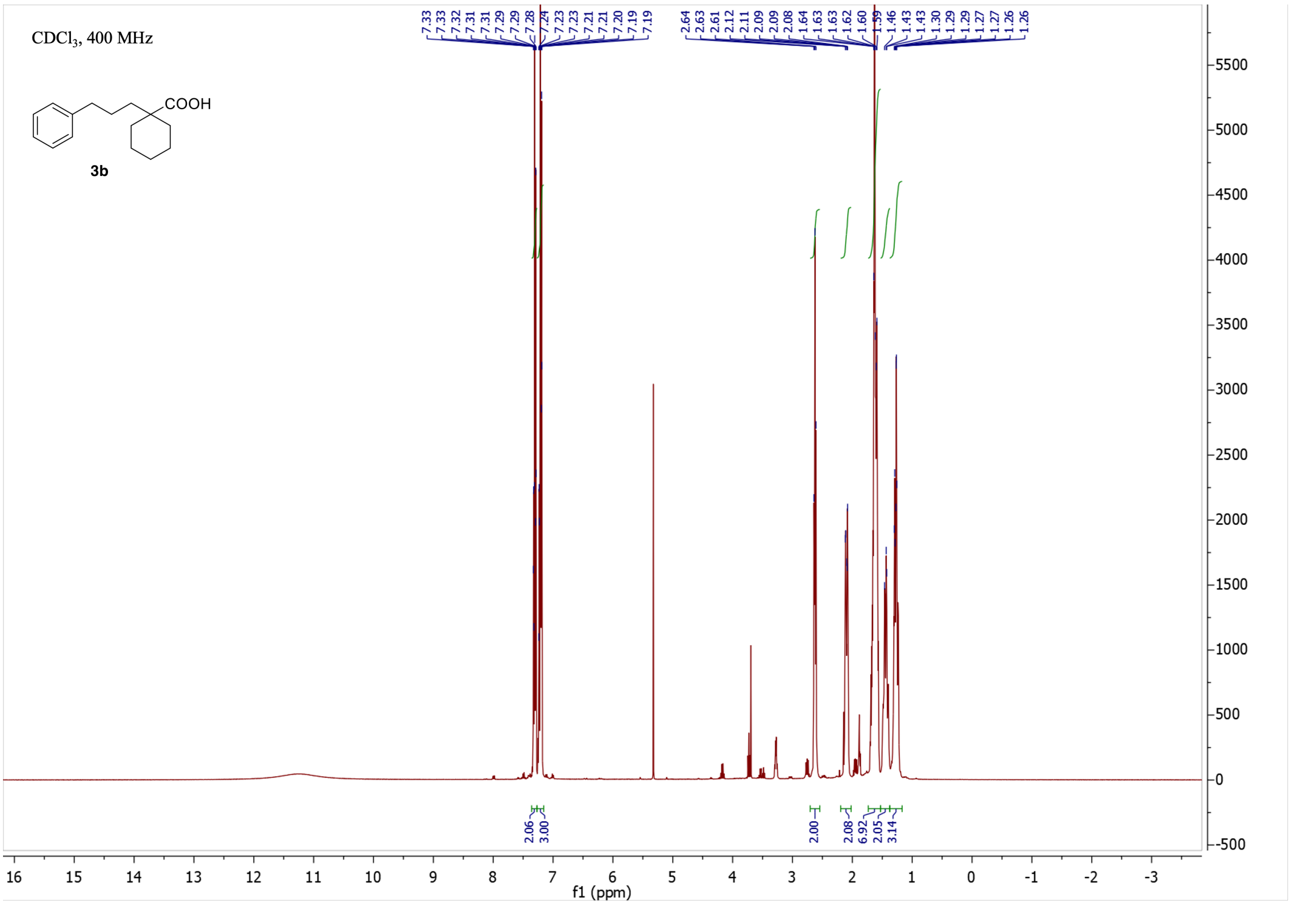
CDCl$_3$, 400 MHz
COOH
3b
f1 (ppm)

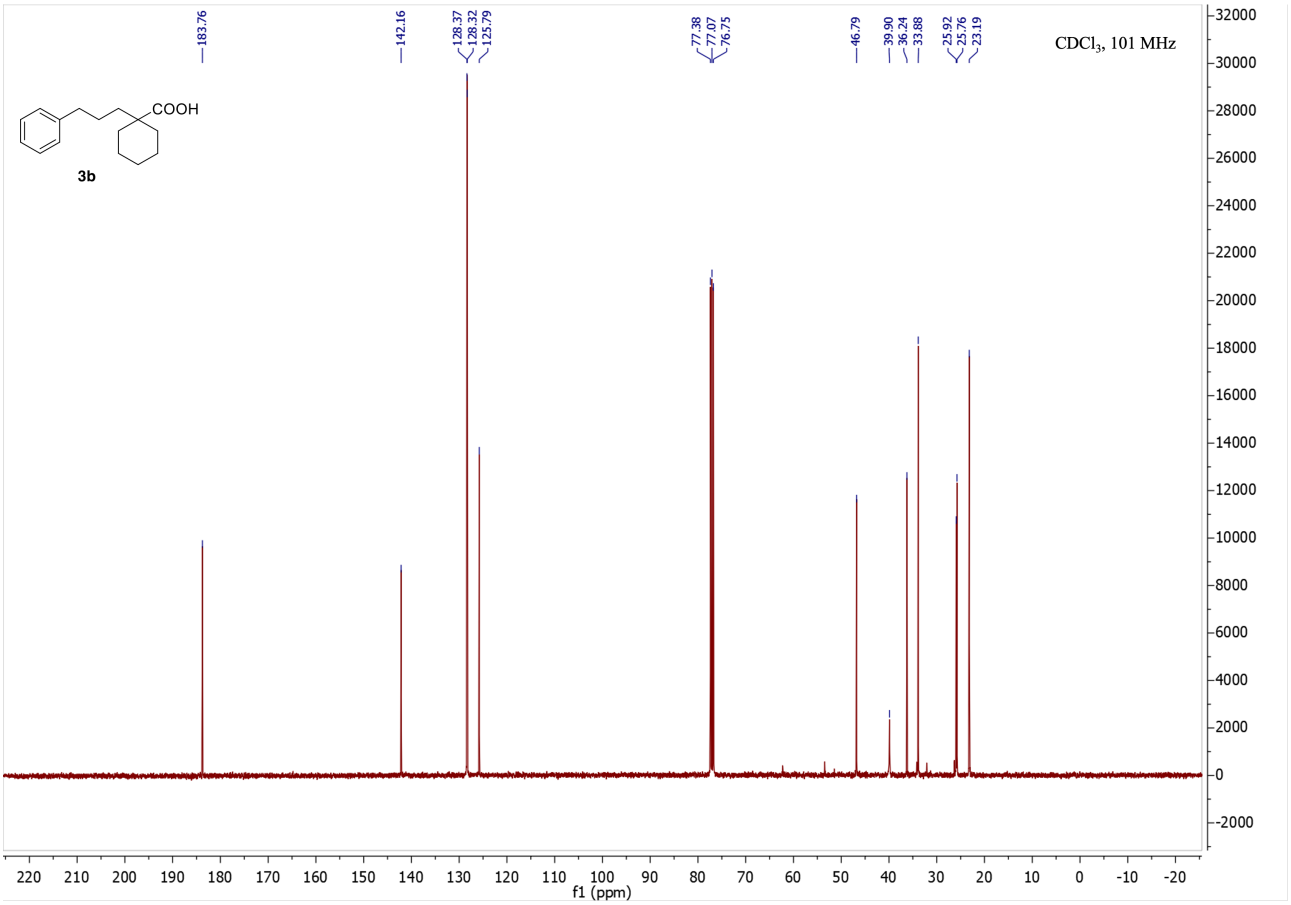
CDCl$_3$, 101 MHz
COOH
3b
183.76
142.16
128.37
128.32
125.79
77.38
77.07
76.75
46.79
39.90
36.24
33.88
25.92
25.76
23.19
f1 (ppm)

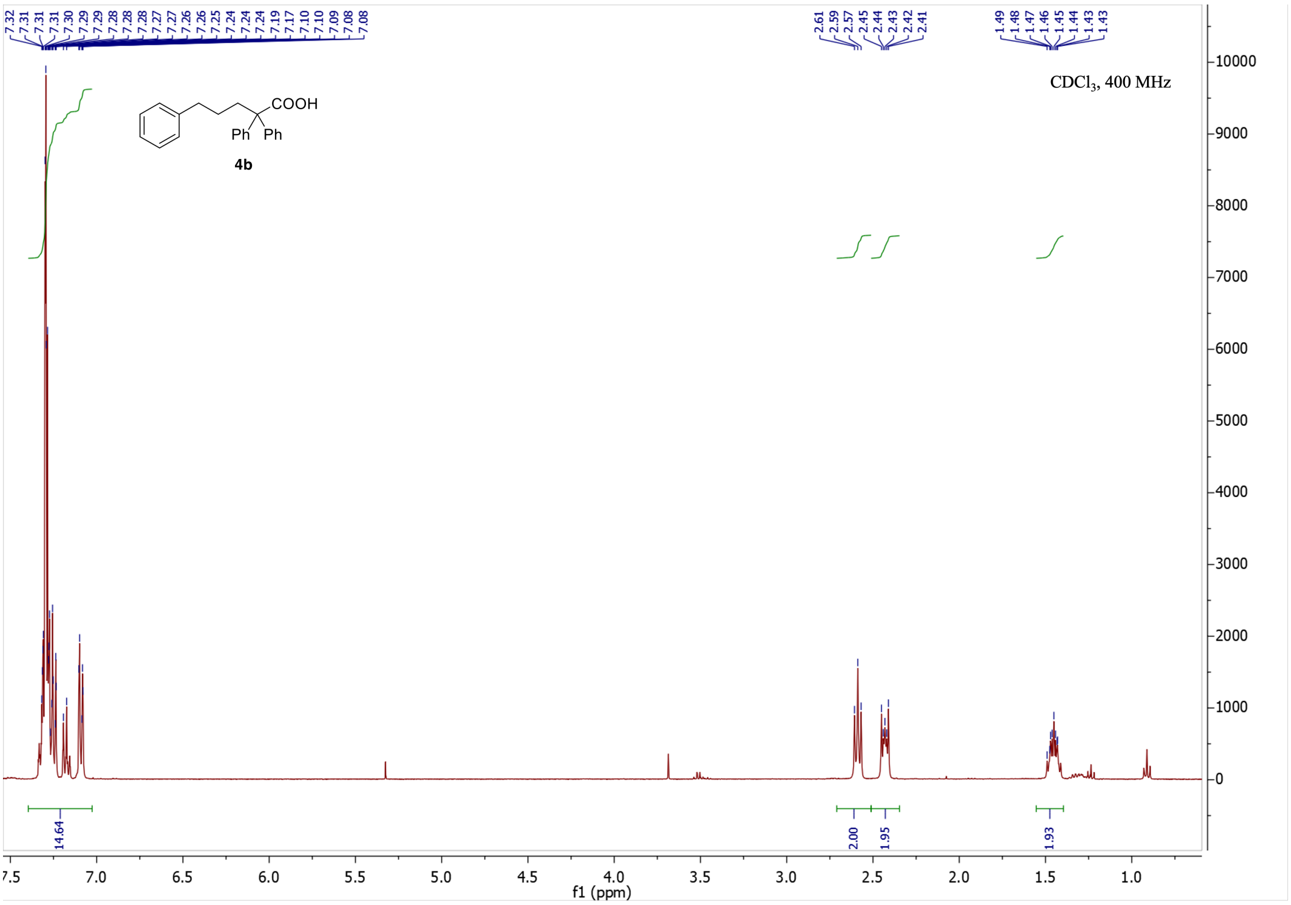
CDCl3, 400 MHz
COOH
Ph
Ph
4b
14.64
2.00
1.95
1.93
f1 (ppm)

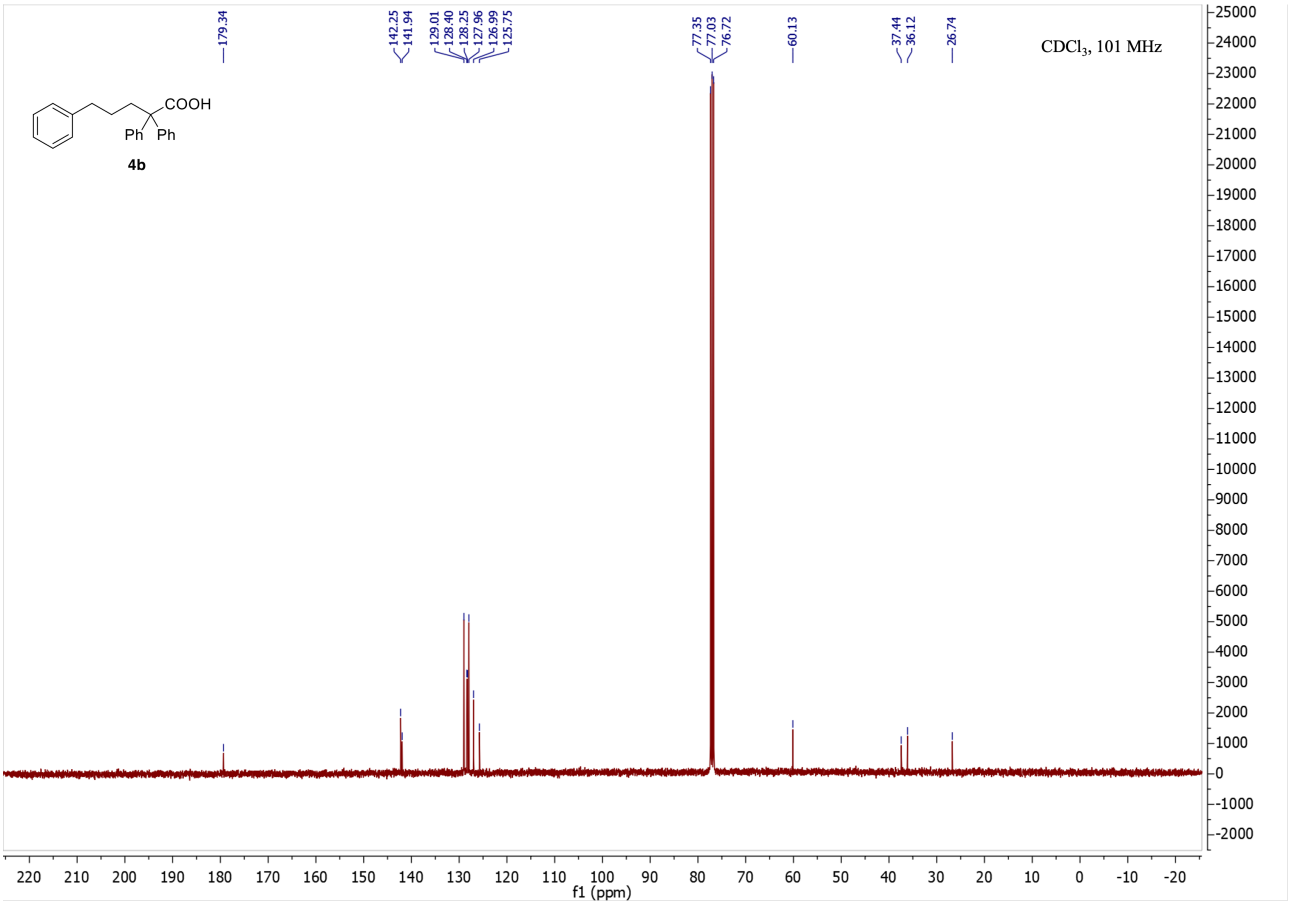
179.34
142.25
141.94
129.01
128.40
128.25
127.96
126.99
125.75
77.35
77.03
76.72
60.13
37.44
36.12
26.74
CDCl3, 101 MHz
COOH
Ph
Ph
4b
f1 (ppm)

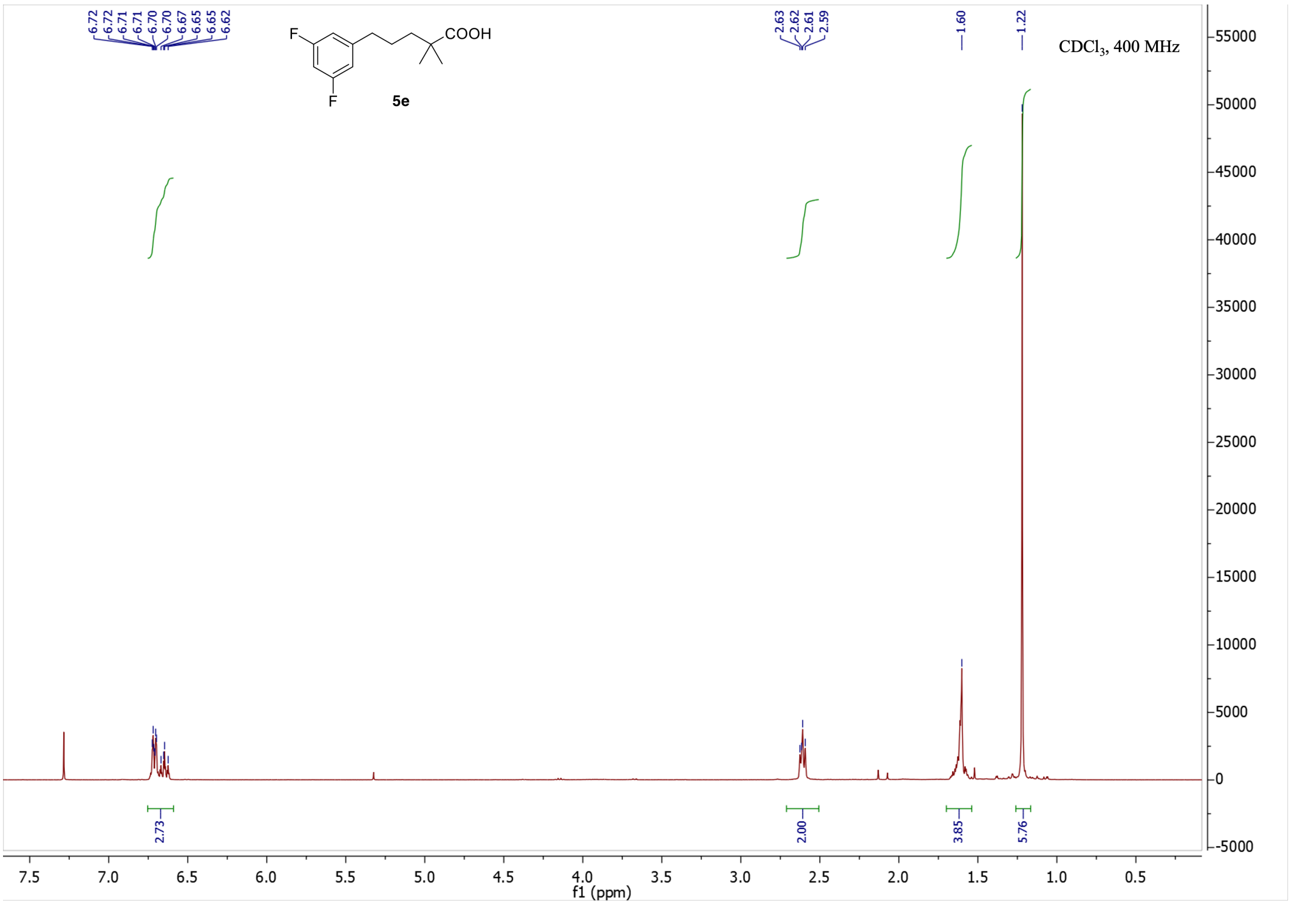
6.72
6.72
6.71
6.71
6.70
6.70
6.67
6.65
6.65
6.62
F
F
COOH
5e
2.63
2.62
2.61
2.59
1.60
1.22
CDCl3, 400 MHz
2.73
2.00
3.85
5.76
f1 (ppm)

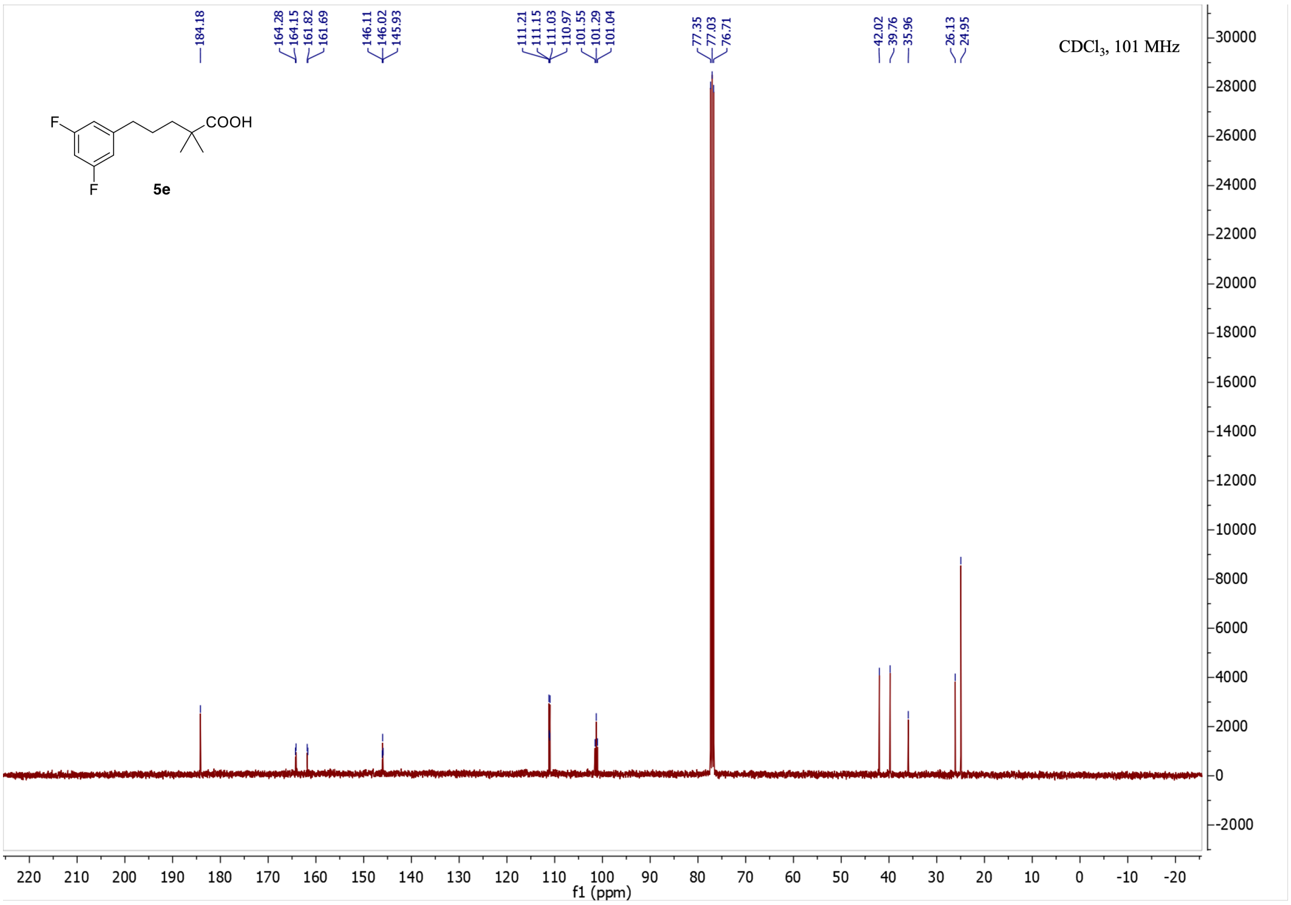
184.18
164.28
164.15
161.82
161.69
146.11
146.02
145.93
111.21
111.15
111.03
110.97
101.55
101.29
101.04
77.35
77.03
76.71
42.02
39.76
35.96
26.13
24.95
CDCl3, 101 MHz
COOH
F
F
5e
f1 (ppm)

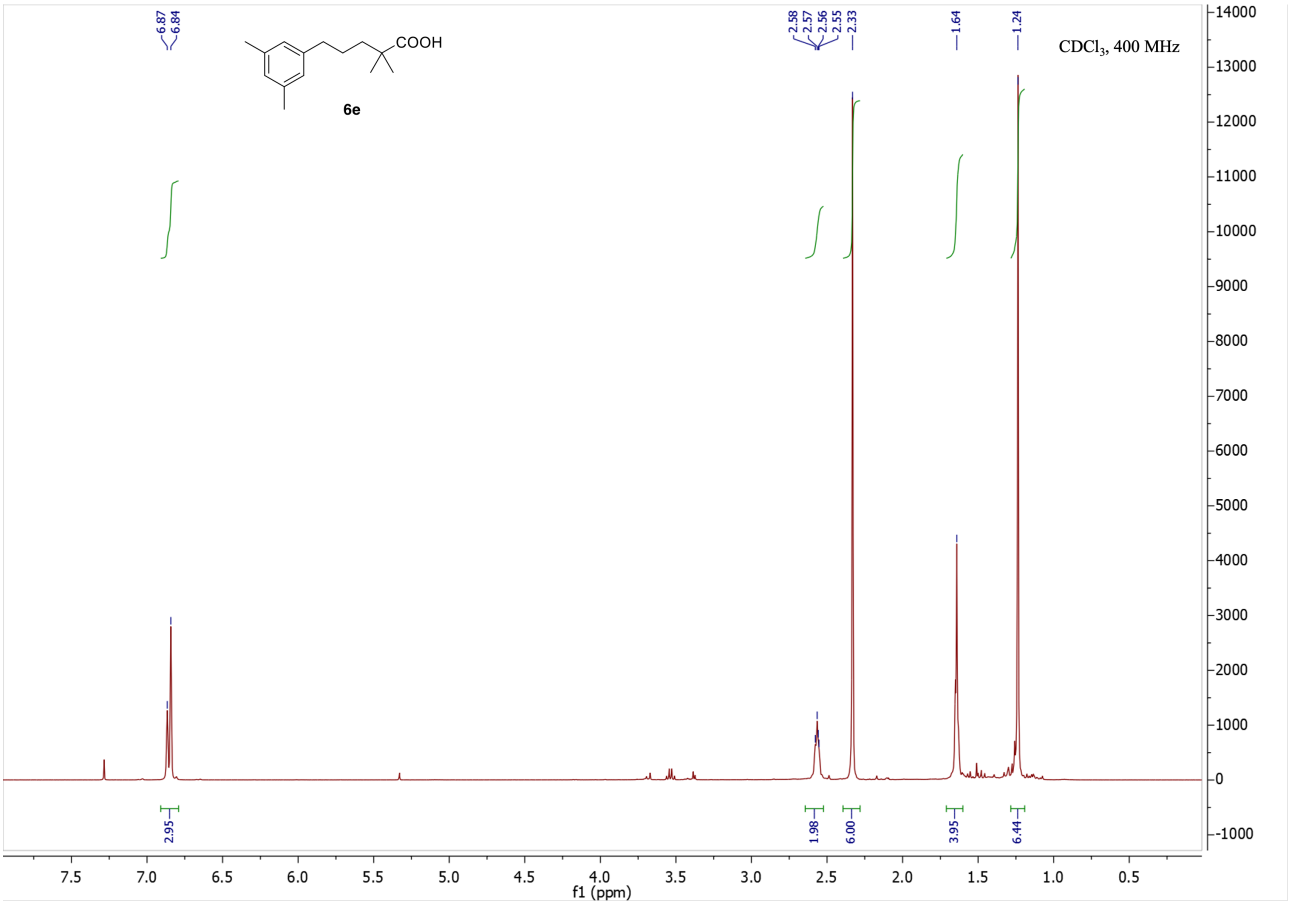
6.87
6.84
2.58
2.57
2.56
2.55
2.33
1.64
1.24
CDCl3, 400 MHz
COOH
6e
2.95
1.98
6.00
3.95
6.44
f1 (ppm)

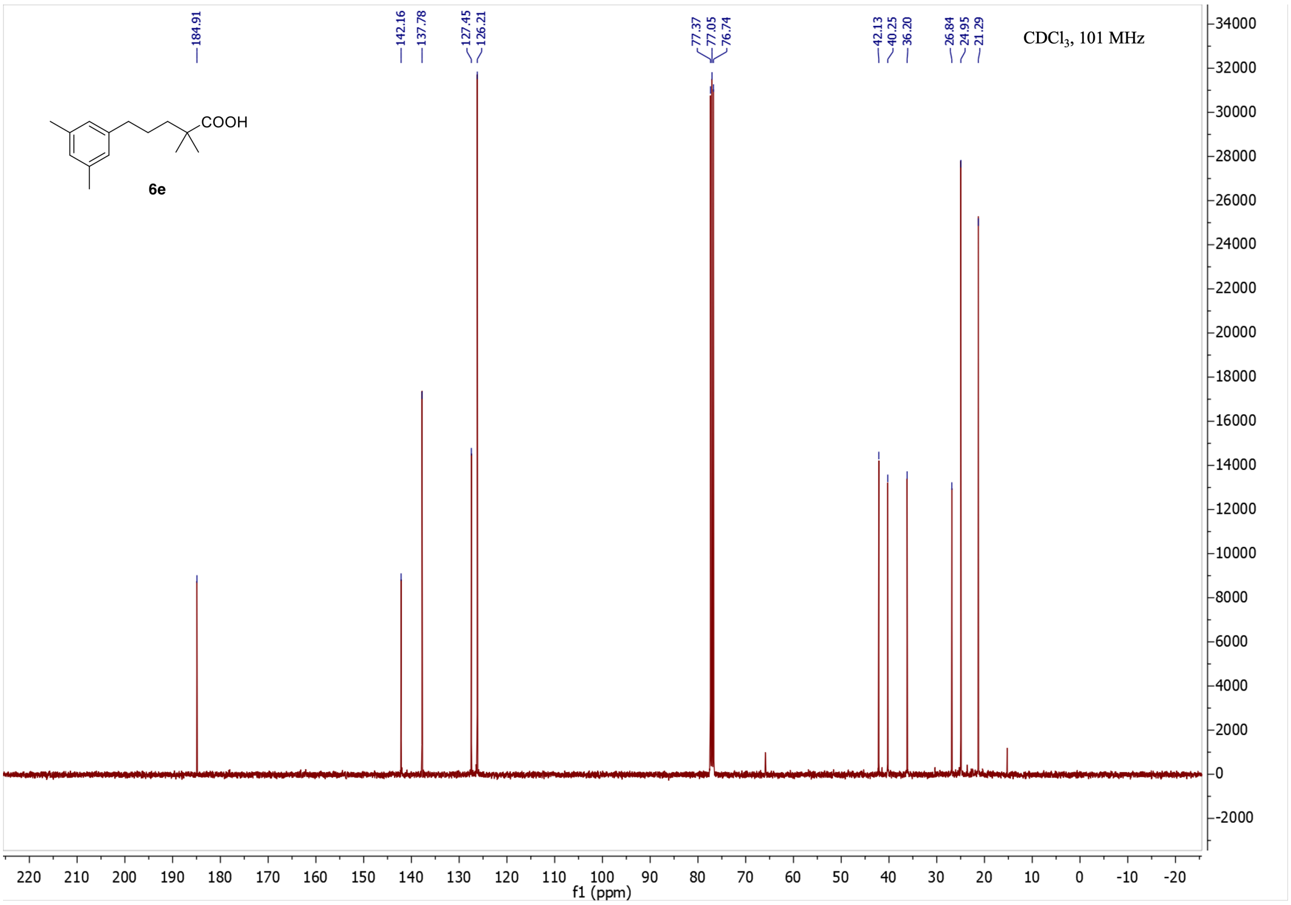
184.91
142.16
137.78
127.45
126.21
77.37
77.05
76.74
42.13
40.25
36.20
26.84
24.95
21.29
CDCl3, 101 MHz
COOH
6e
f1 (ppm)

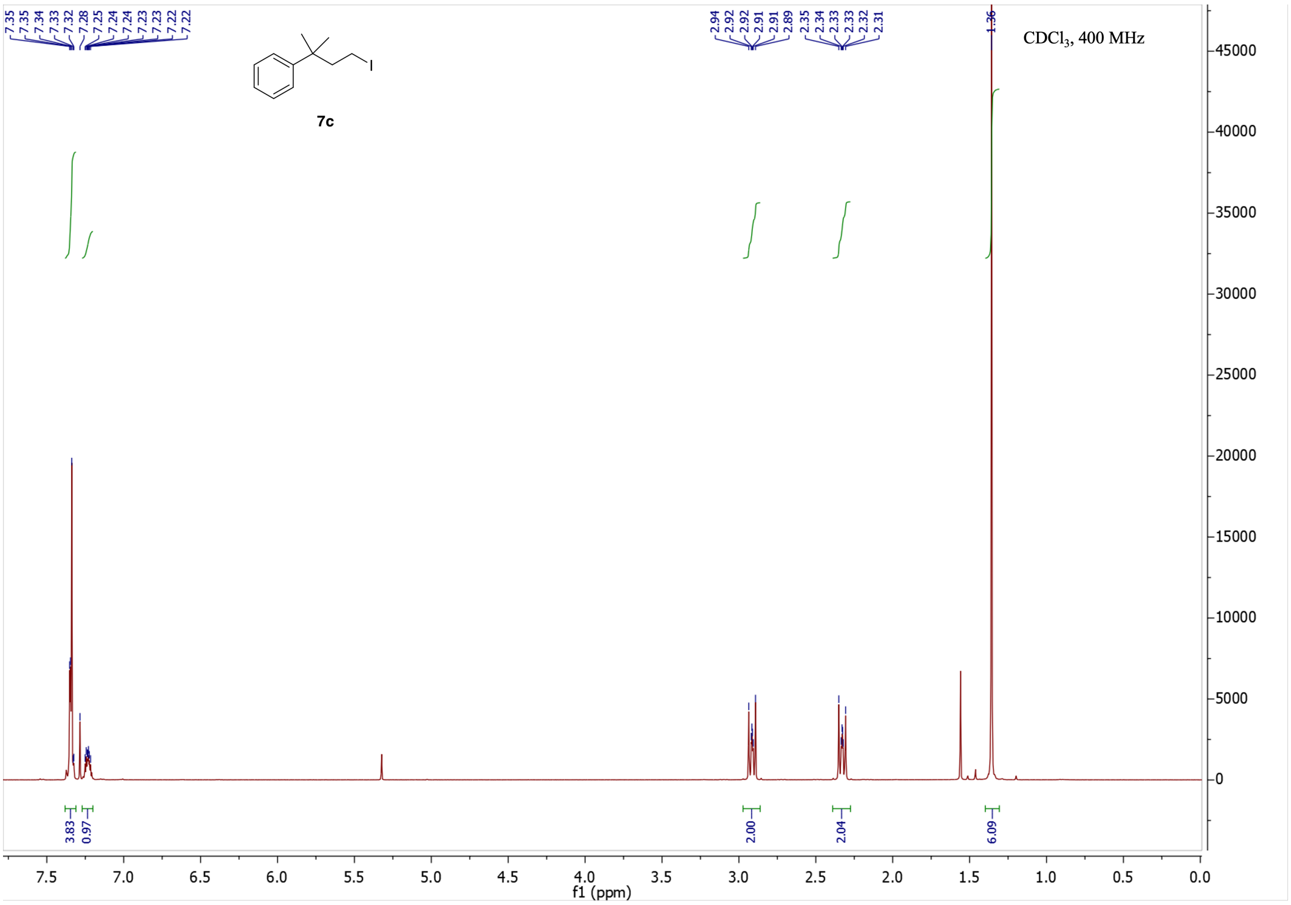
7.35
7.35
7.34
7.33
7.32
7.28
7.25
7.24
7.24
7.23
7.23
7.22
7.22
2.94
2.92
2.92
2.91
2.91
2.89
2.35
2.34
2.33
2.33
2.32
2.31
1.36
CDCl3, 400 MHz
7c
3.83
0.97
2.00
2.04
6.09
f1 (ppm)

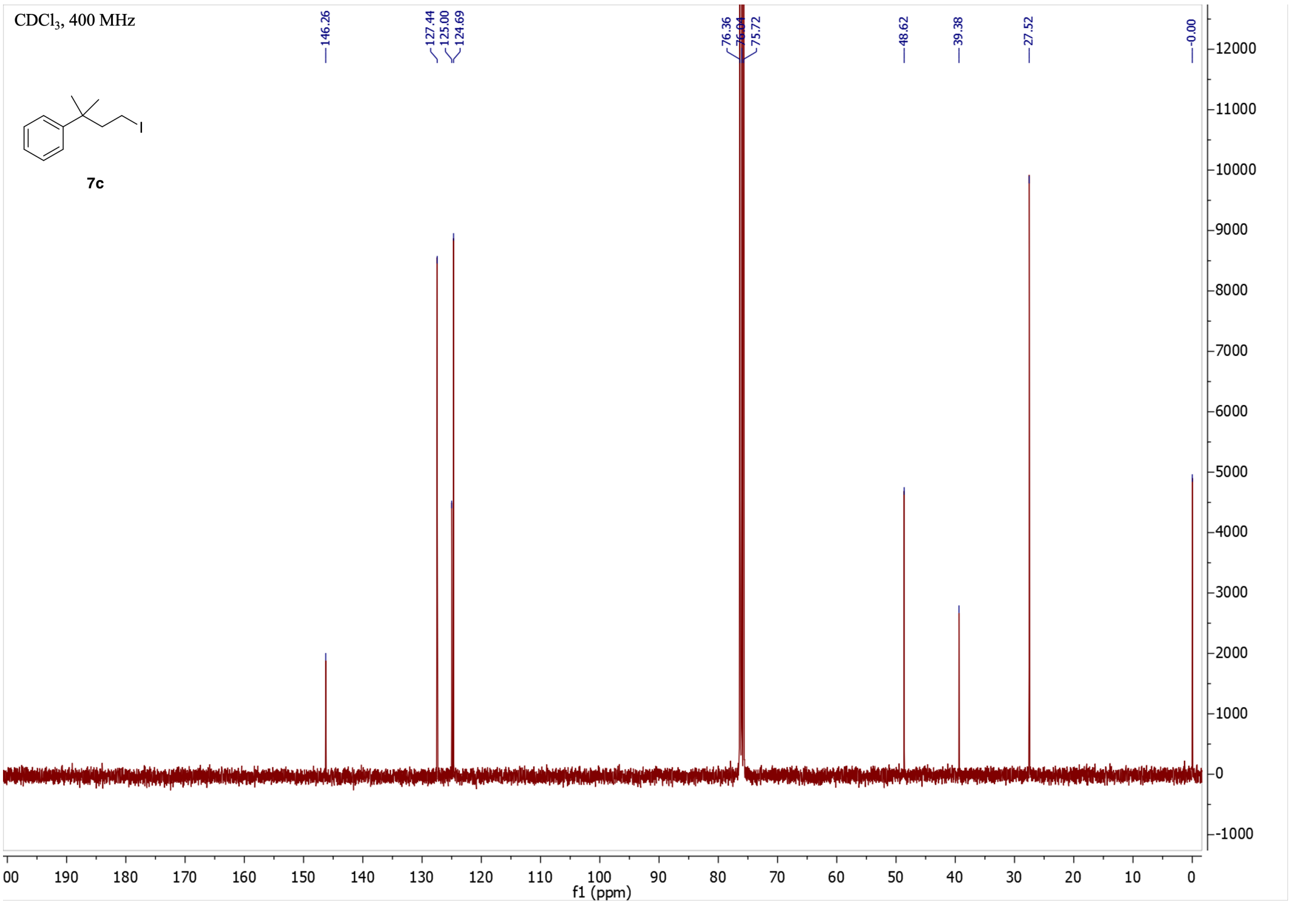
CDCl3, 400 MHz
7c
146.26
127.44
125.00
124.69
76.36
76.04
75.72
48.62
39.38
27.52
-0.00
f1 (ppm)

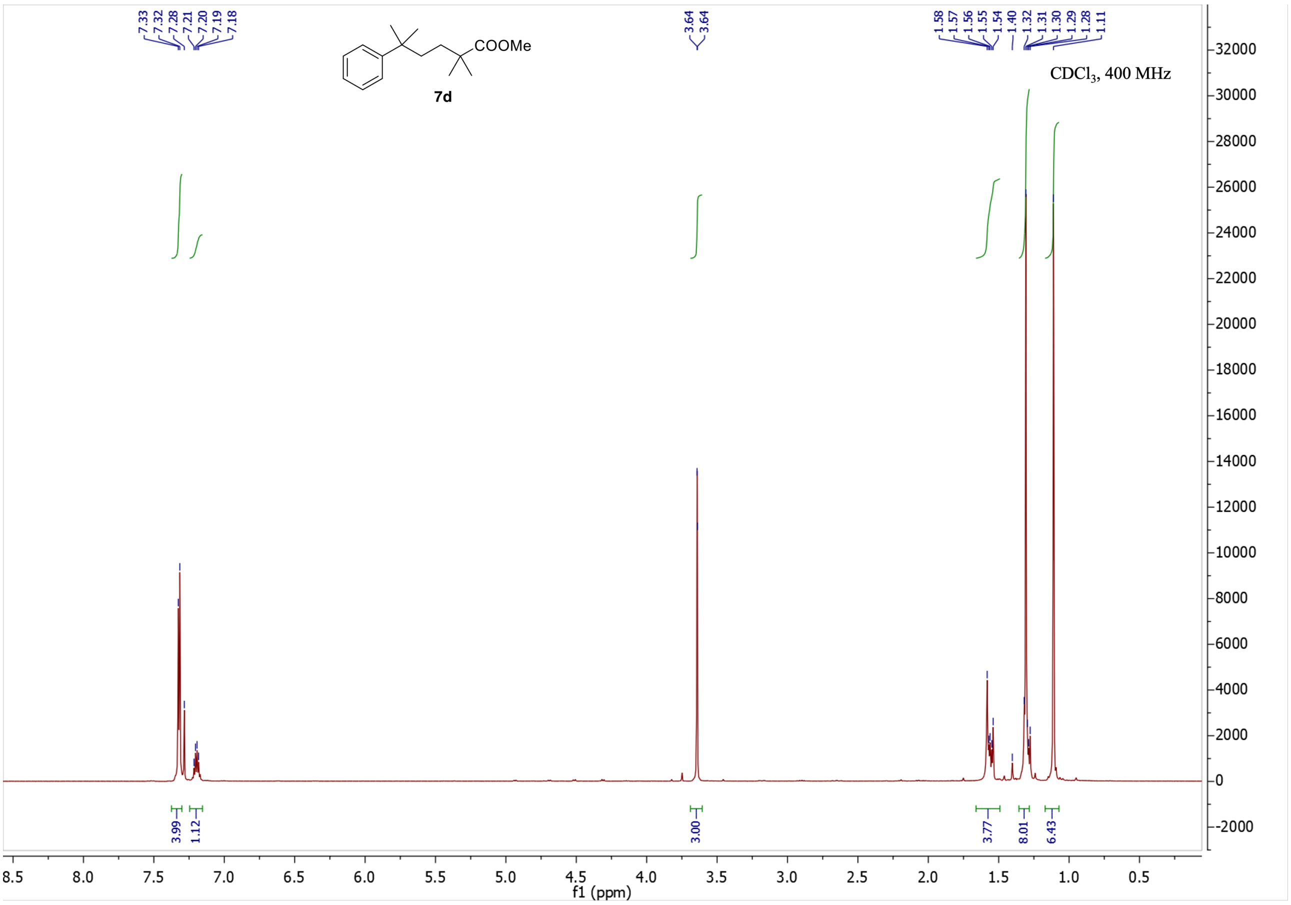
7.33
7.32
7.28
7.21
7.20
7.19
7.18
3.64
3.64
1.58
1.57
1.56
1.55
1.54
1.40
1.32
1.31
1.30
1.29
1.28
1.11
COOMe
7d
CDCl3, 400 MHz
3.99
1.12
3.00
3.77
8.01
6.43
f1 (ppm)

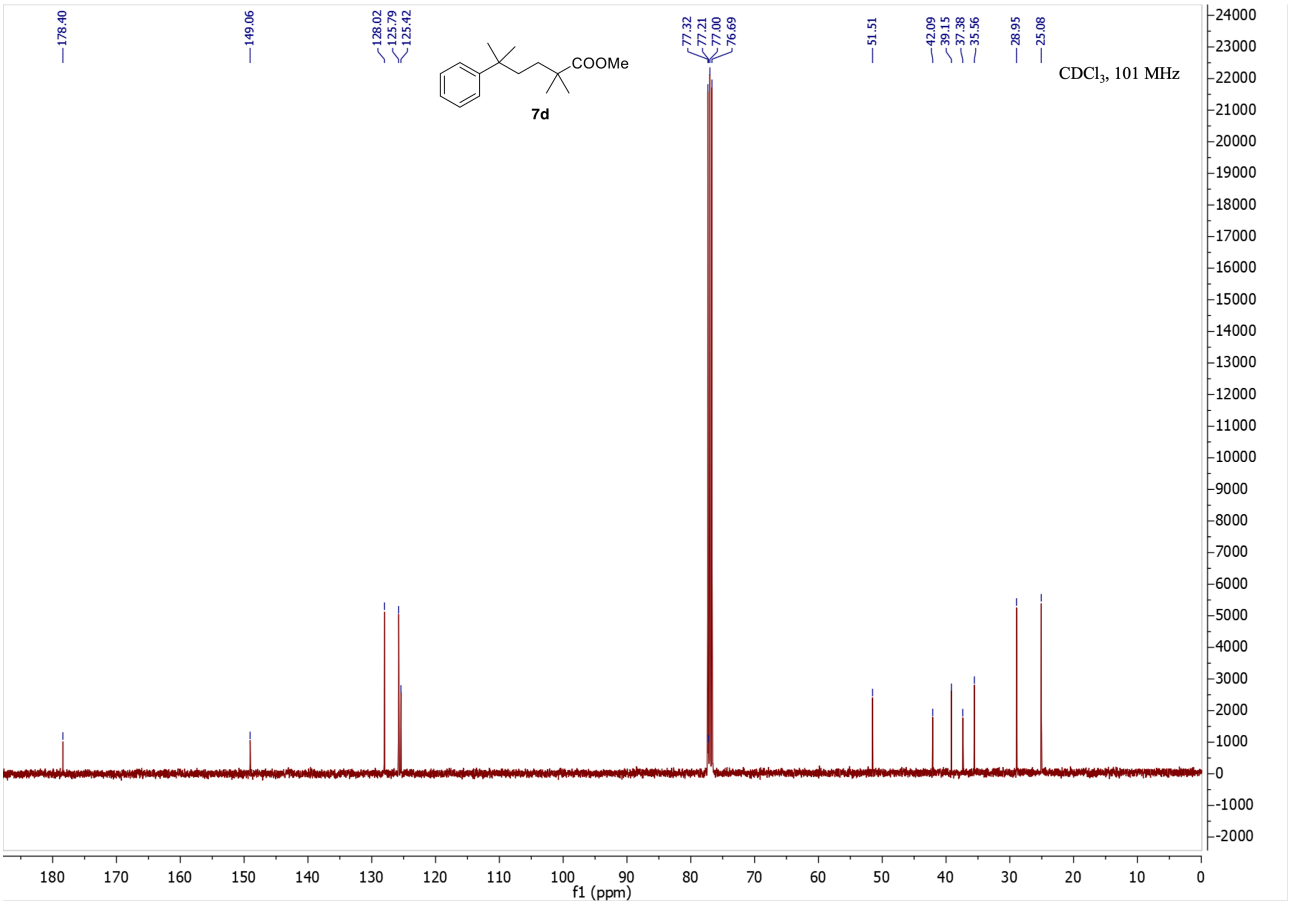
178.40
149.06
128.02
125.79
125.42
77.32
77.21
77.00
76.69
51.51
42.09
39.15
37.38
35.56
28.95
25.08
COOMe
7d
CDCl3, 101 MHz
f1 (ppm)

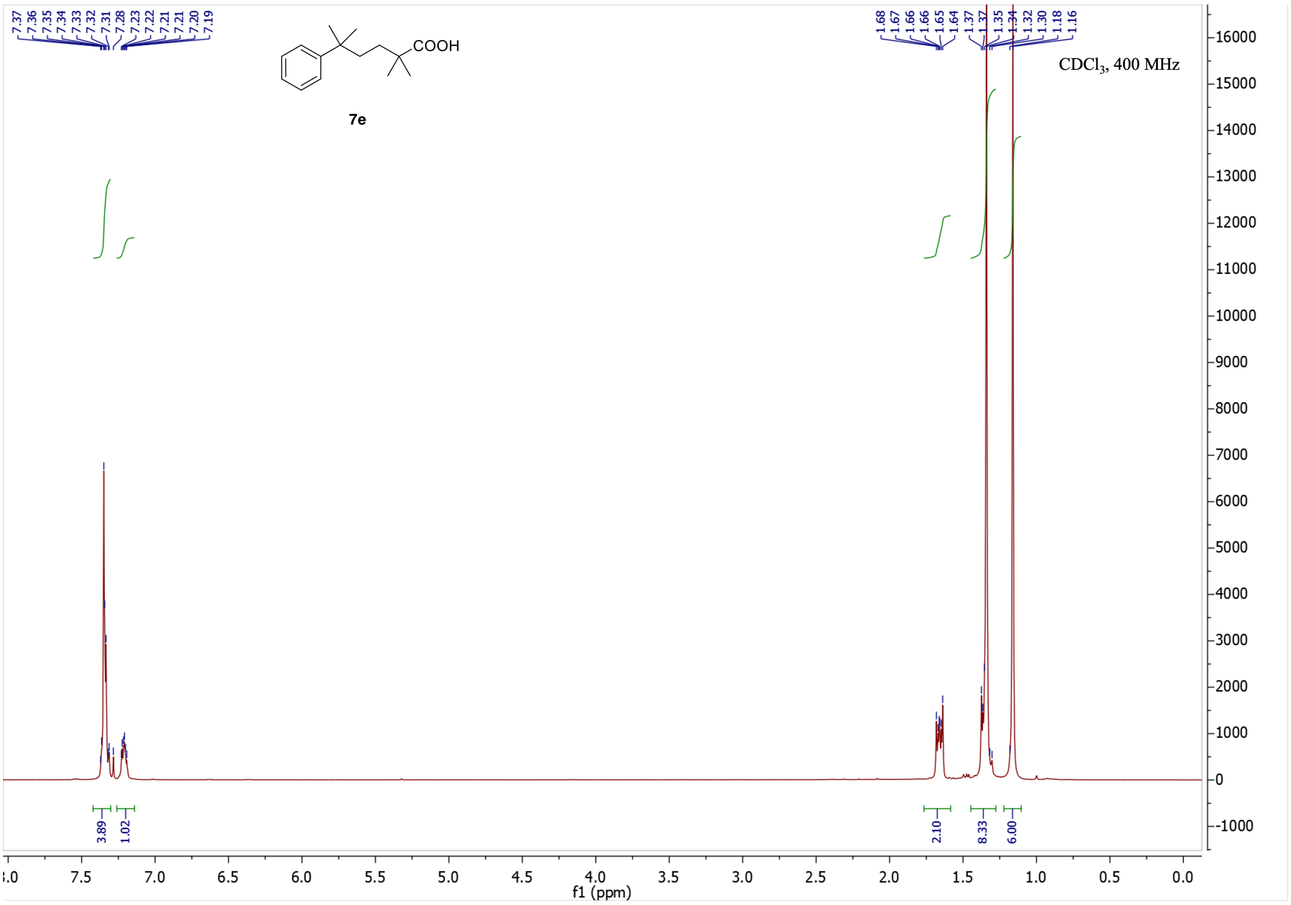
7.37
7.36
7.35
7.34
7.33
7.32
7.31
7.28
7.23
7.22
7.21
7.21
7.20
7.19
COOH
7e
1.68
1.67
1.66
1.66
1.65
1.64
1.37
1.37
1.35
1.34
1.32
1.30
1.18
1.16
CDCl3, 400 MHz
3.89
1.02
2.10
8.33
6.00
f1 (ppm)

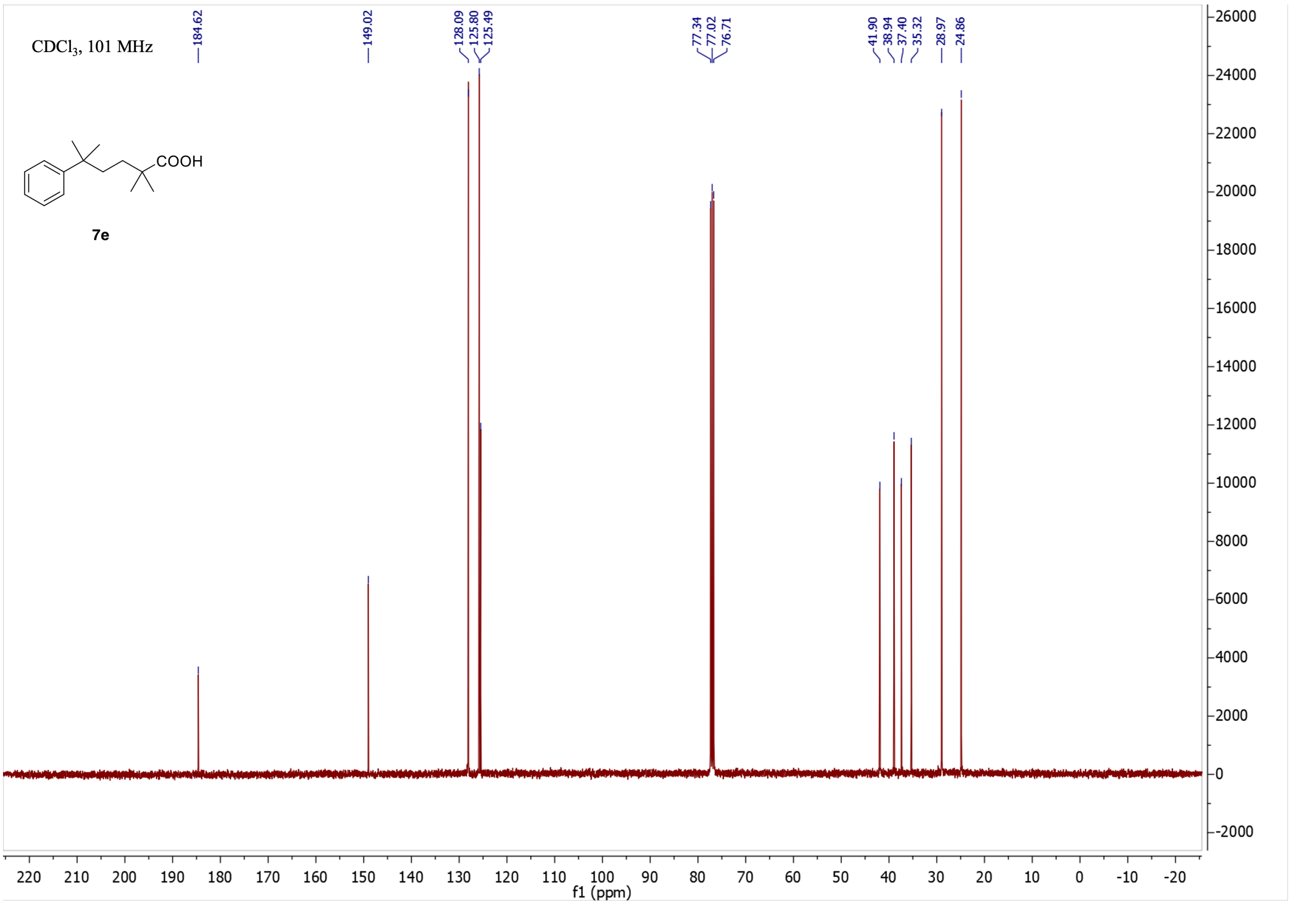
CDCl3, 101 MHz
184.62
149.02
128.09
125.80
125.49
77.34
77.02
76.71
41.90
38.94
37.40
35.32
28.97
24.86
COOH
7e
f1 (ppm)

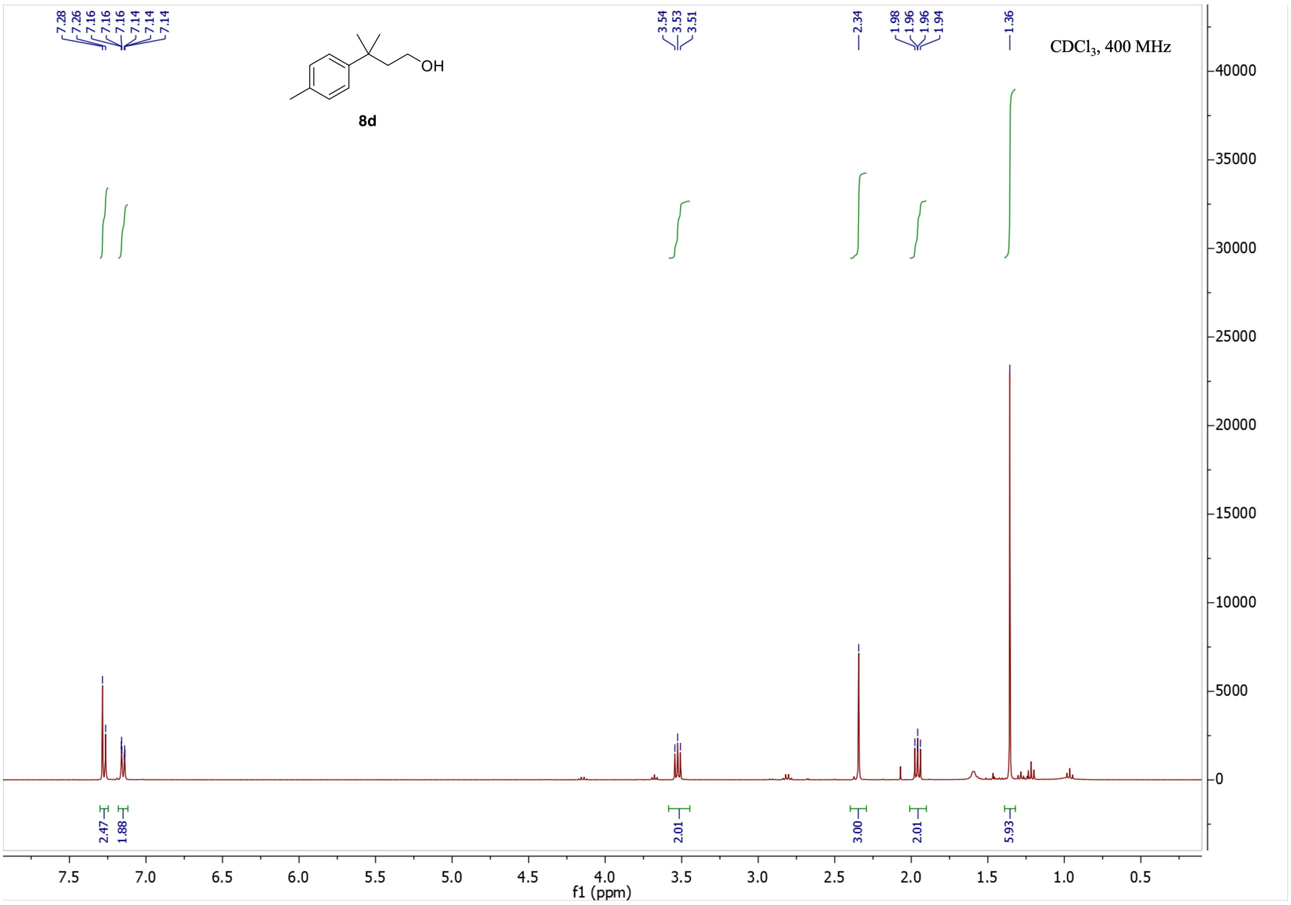
CDCl$_3$, 400 MHz
8d
OH
f1 (ppm)

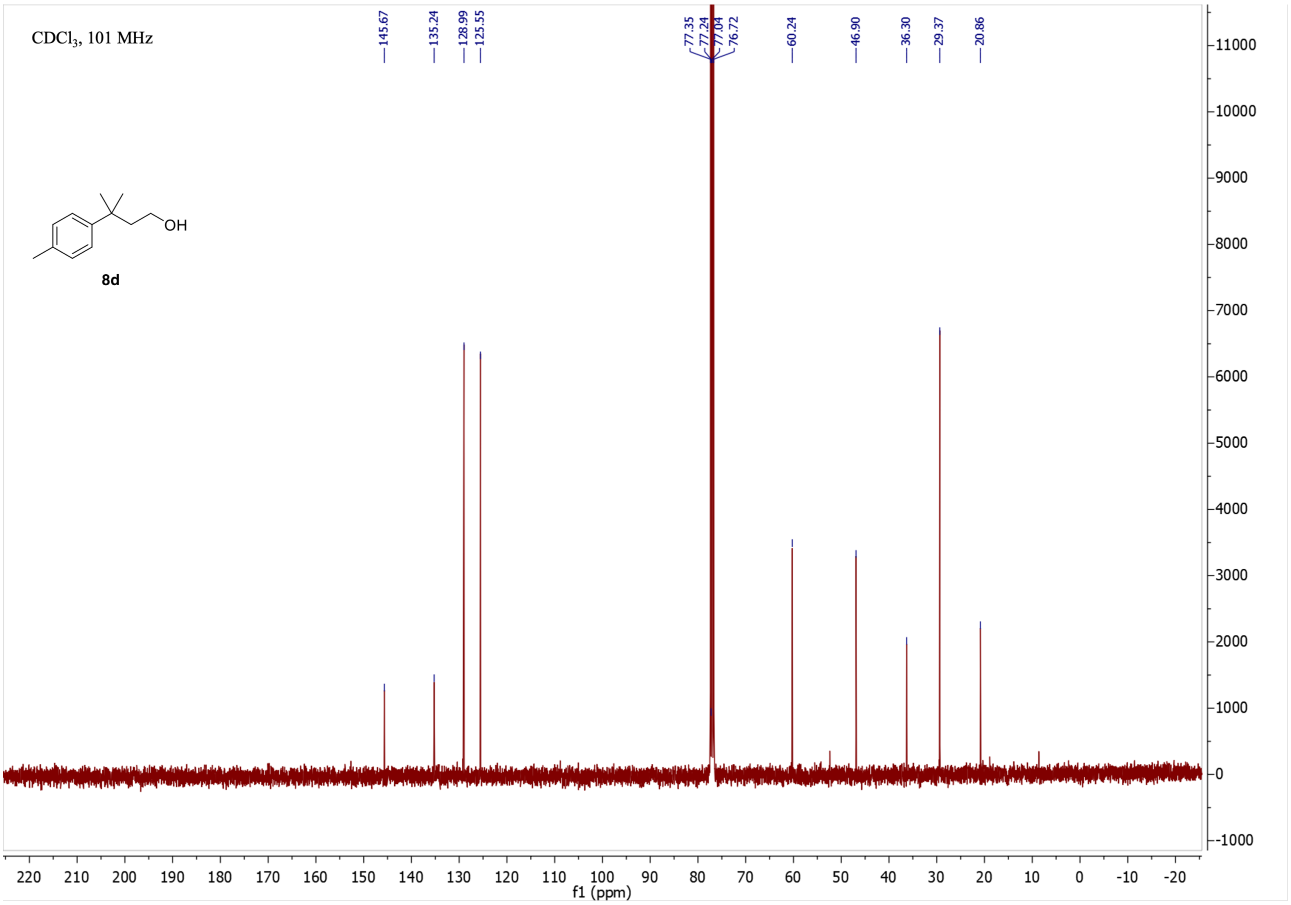
CDCl$_3$, 101 MHz
8d
OH
f1 (ppm)

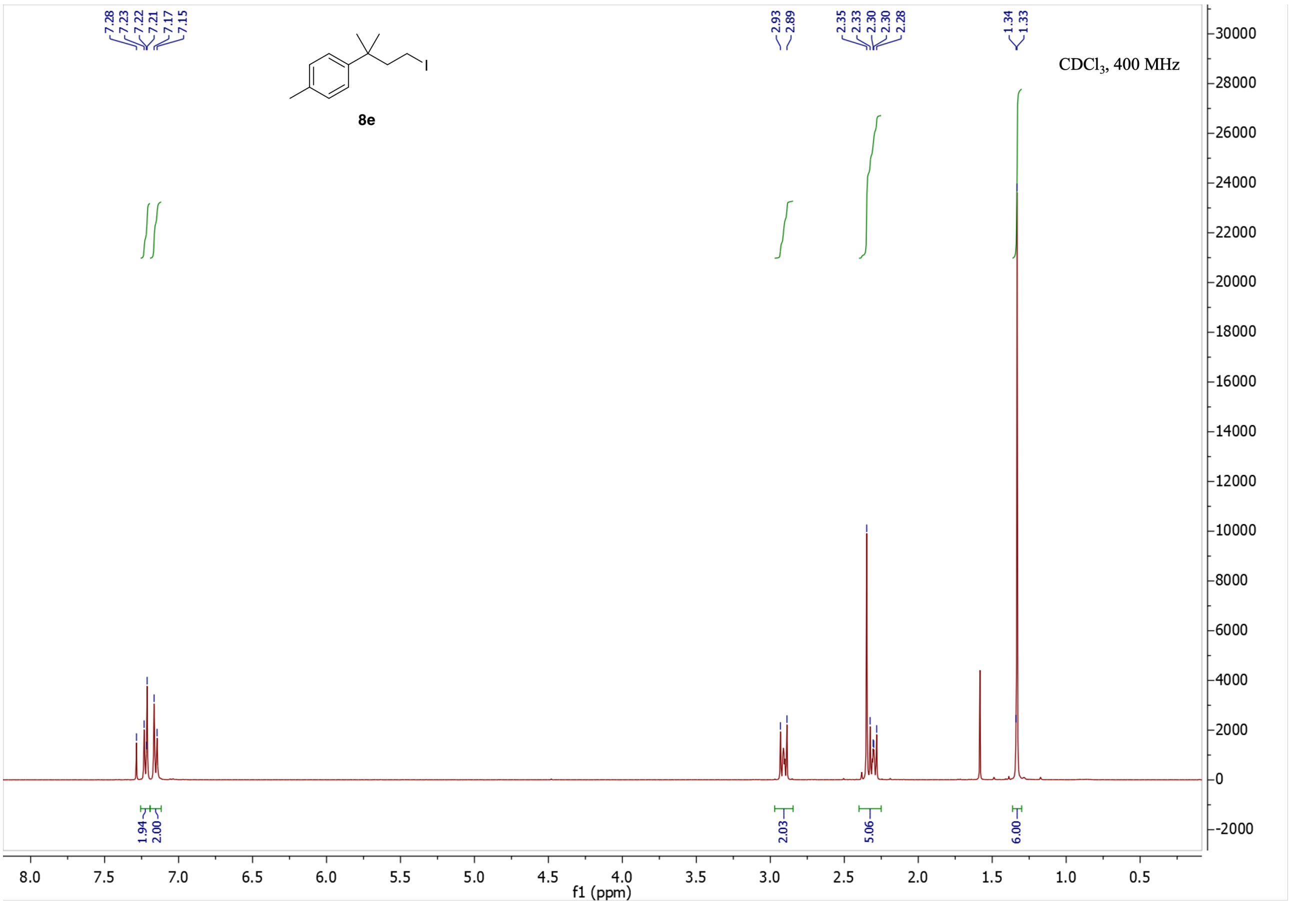

CDCl3, 400 MHz
8e
7.28
7.23
7.22
7.21
7.17
7.15
2.93
2.89
2.35
2.33
2.30
2.30
2.28
1.34
1.33
1.94
2.00
2.03
5.06
6.00
f1 (ppm)

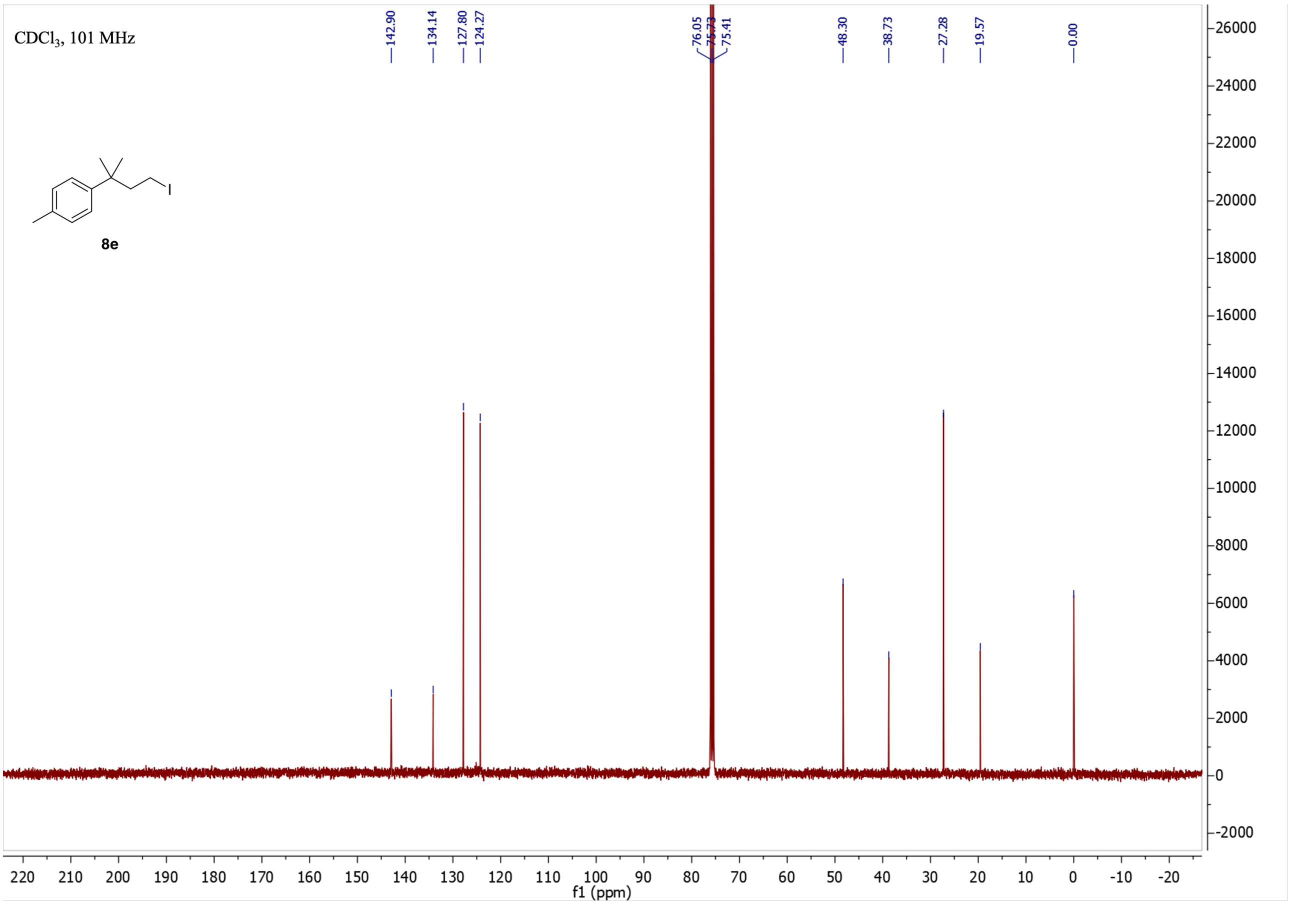

CDCl3, 101 MHz
8e
142.90
134.14
127.80
124.27
76.05
75.73
75.41
48.30
38.73
27.28
19.57
0.00
f1 (ppm)

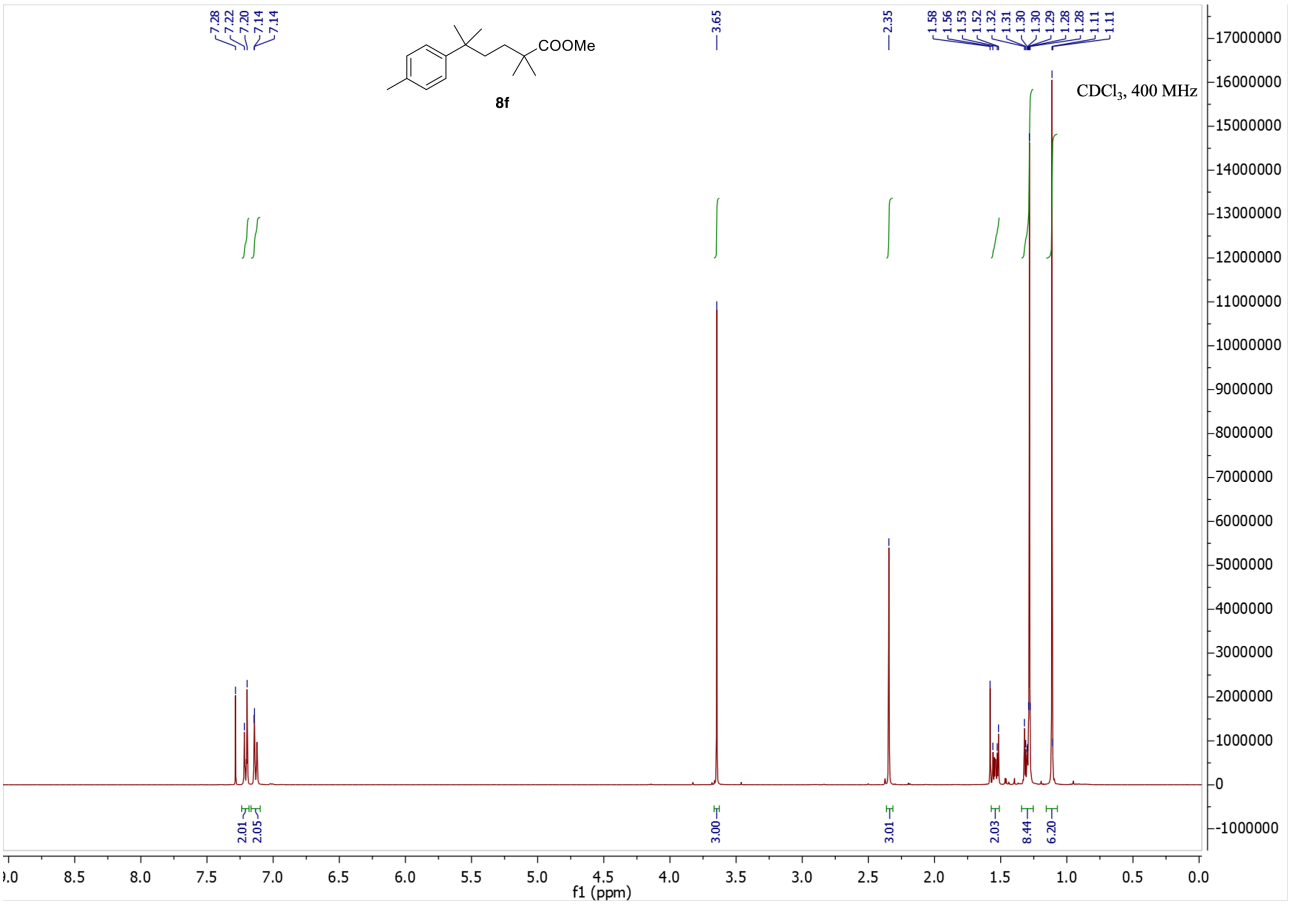

COOMe
8f
CDCl3, 400 MHz
f1 (ppm)

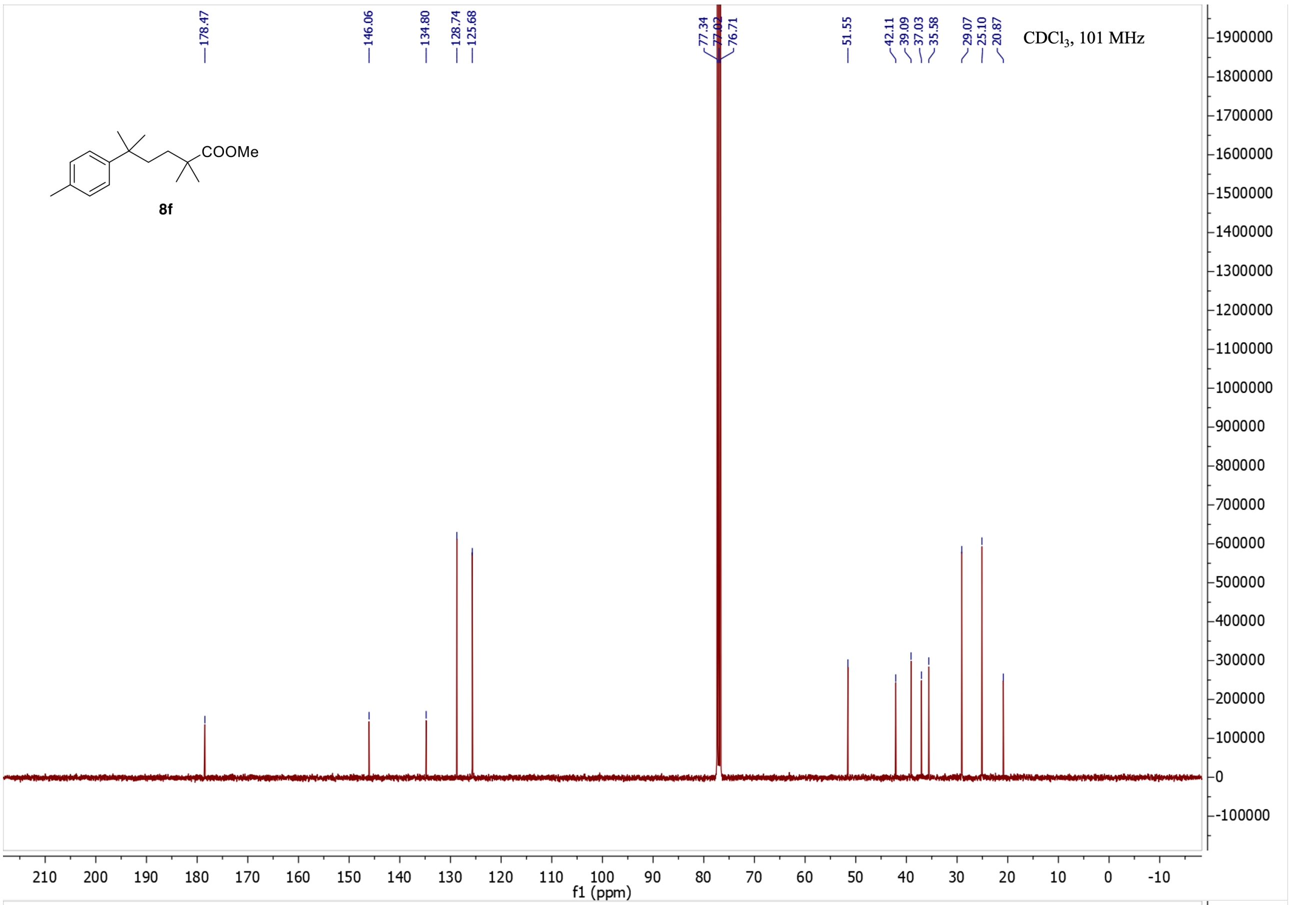
178.47
146.06
134.80
128.74
125.68
77.34
77.02
76.71
51.55
42.11
39.09
37.03
35.58
29.07
25.10
20.87
CDCl3, 101 MHz
COOMe
8f
f1 (ppm)

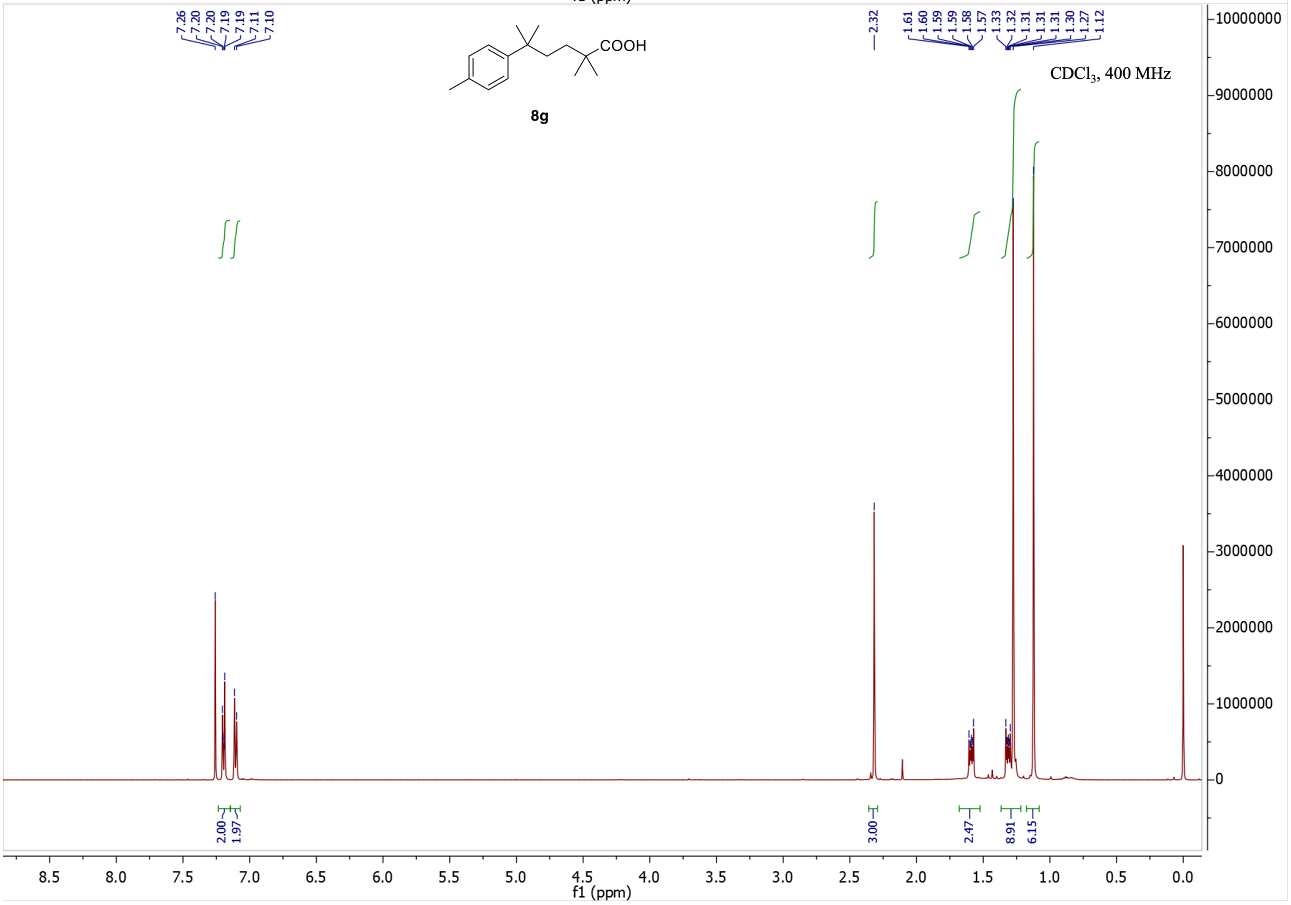
7.26
7.20
7.20
7.19
7.19
7.11
7.10
2.32
1.61
1.60
1.59
1.59
1.58
1.57
1.33
1.32
1.31
1.31
1.30
1.27
1.12
COOH
8g
CDCl3, 400 MHz
2.00
1.97
3.00
2.47
8.91
6.15
f1 (ppm)

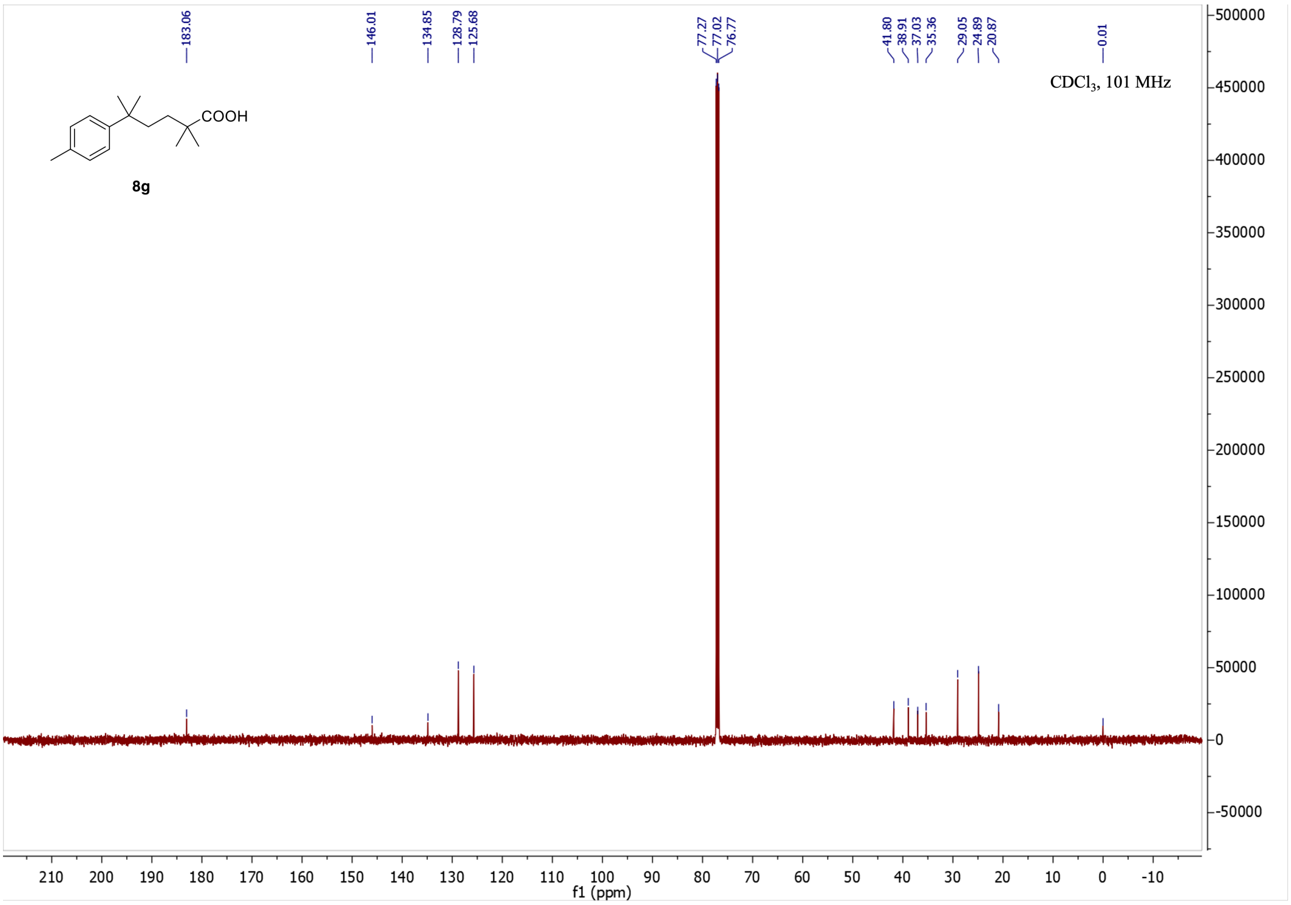
183.06
146.01
134.85
128.79
125.68
77.27
77.02
76.77
41.80
38.91
37.03
35.36
29.05
24.89
20.87
0.01
COOH
8g
CDCl3, 101 MHz
f1 (ppm)

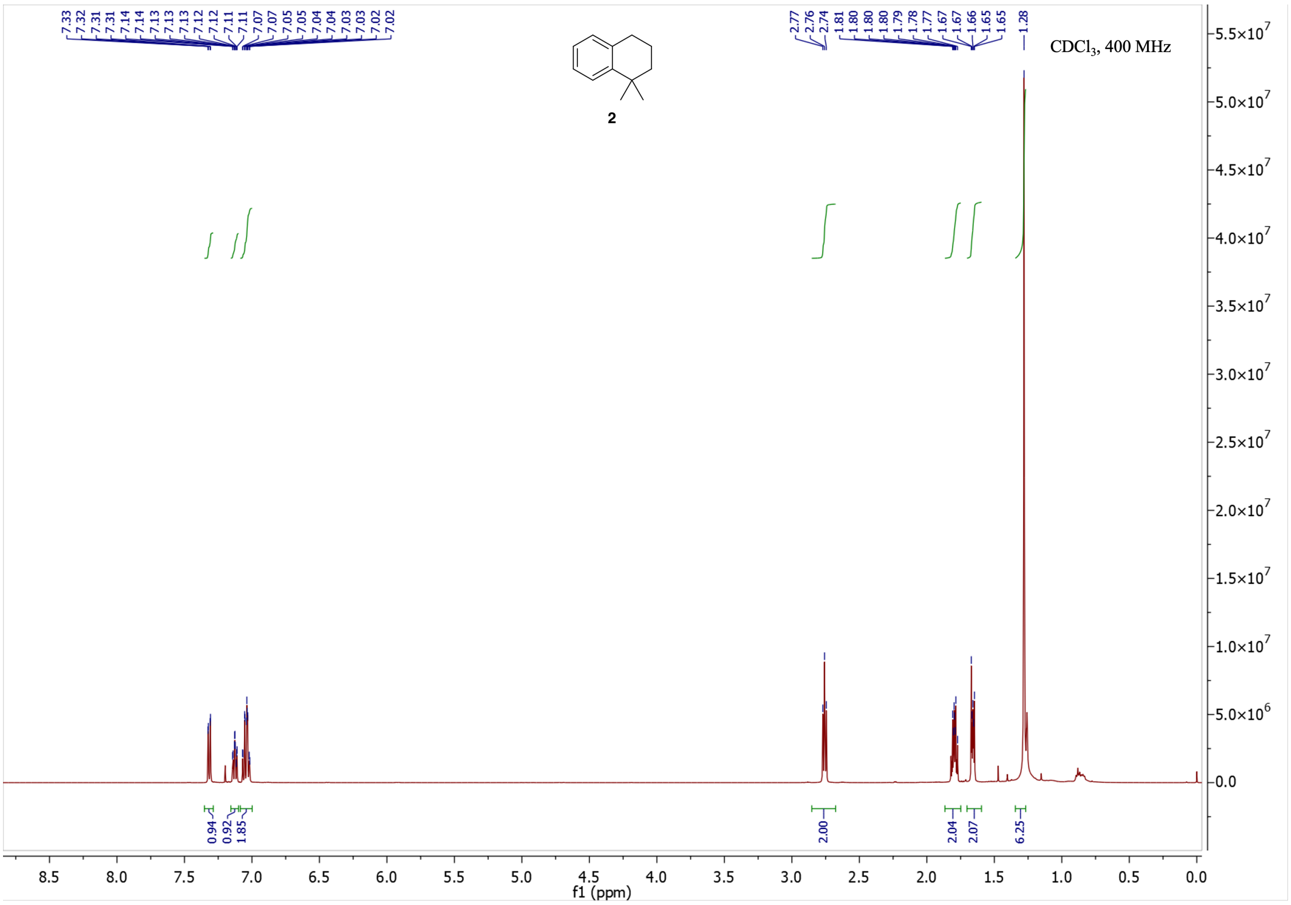
7.33
7.32
7.31
7.31
7.14
7.14
7.13
7.13
7.13
7.12
7.11
7.11
7.07
7.07
7.05
7.04
7.04
7.03
7.03
7.02
7.02
2.77
2.76
2.74
1.81
1.80
1.80
1.79
1.78
1.77
1.67
1.67
1.66
1.65
1.65
1.28
2
CDCl3, 400 MHz
0.94
0.92
1.85
2.00
2.04
2.07
6.25
f1 (ppm)

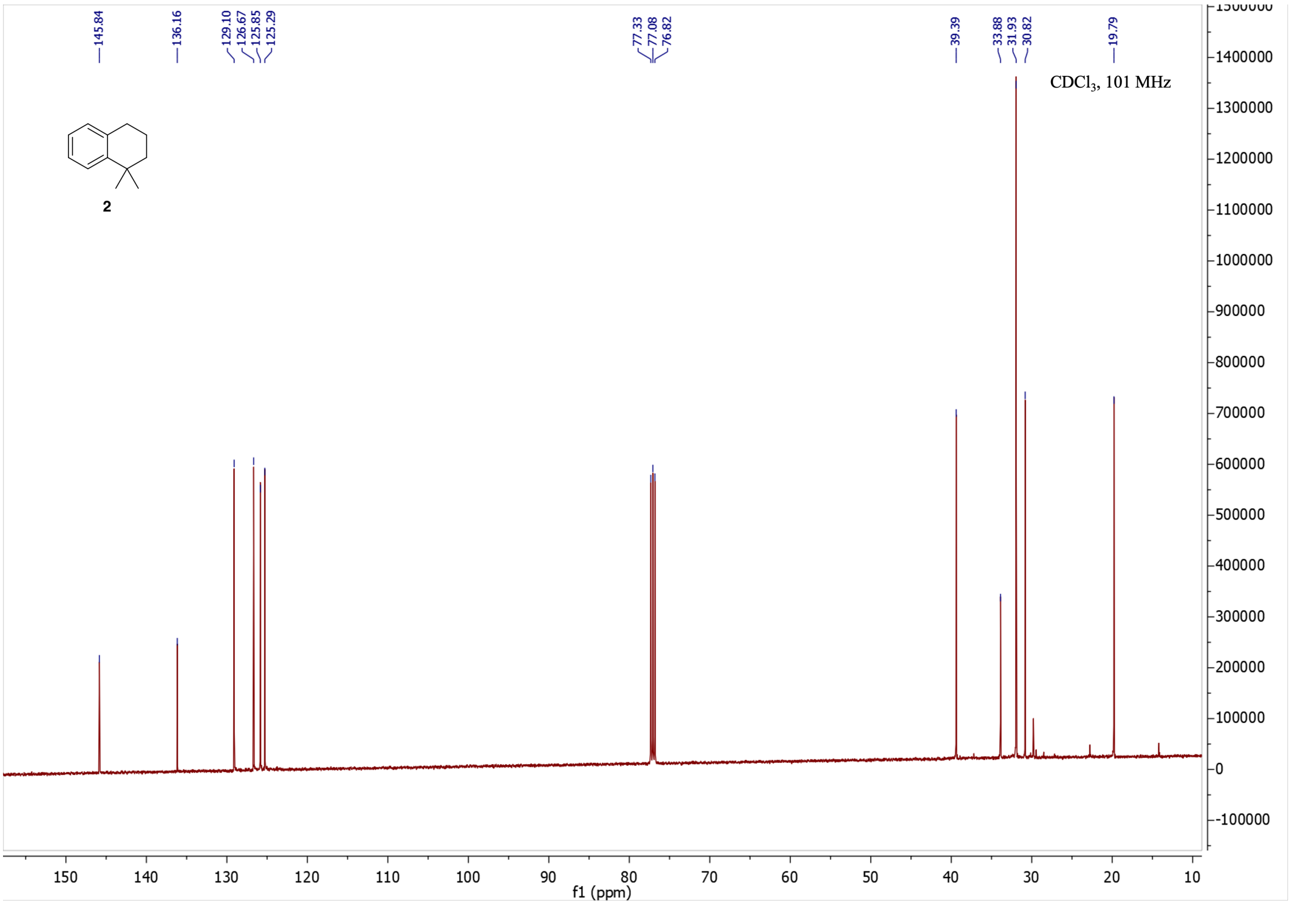
145.84
136.16
129.10
126.67
125.85
125.29
77.33
77.08
76.82
39.39
33.88
31.93
30.82
19.79
CDCl3, 101 MHz
2
f1 (ppm)

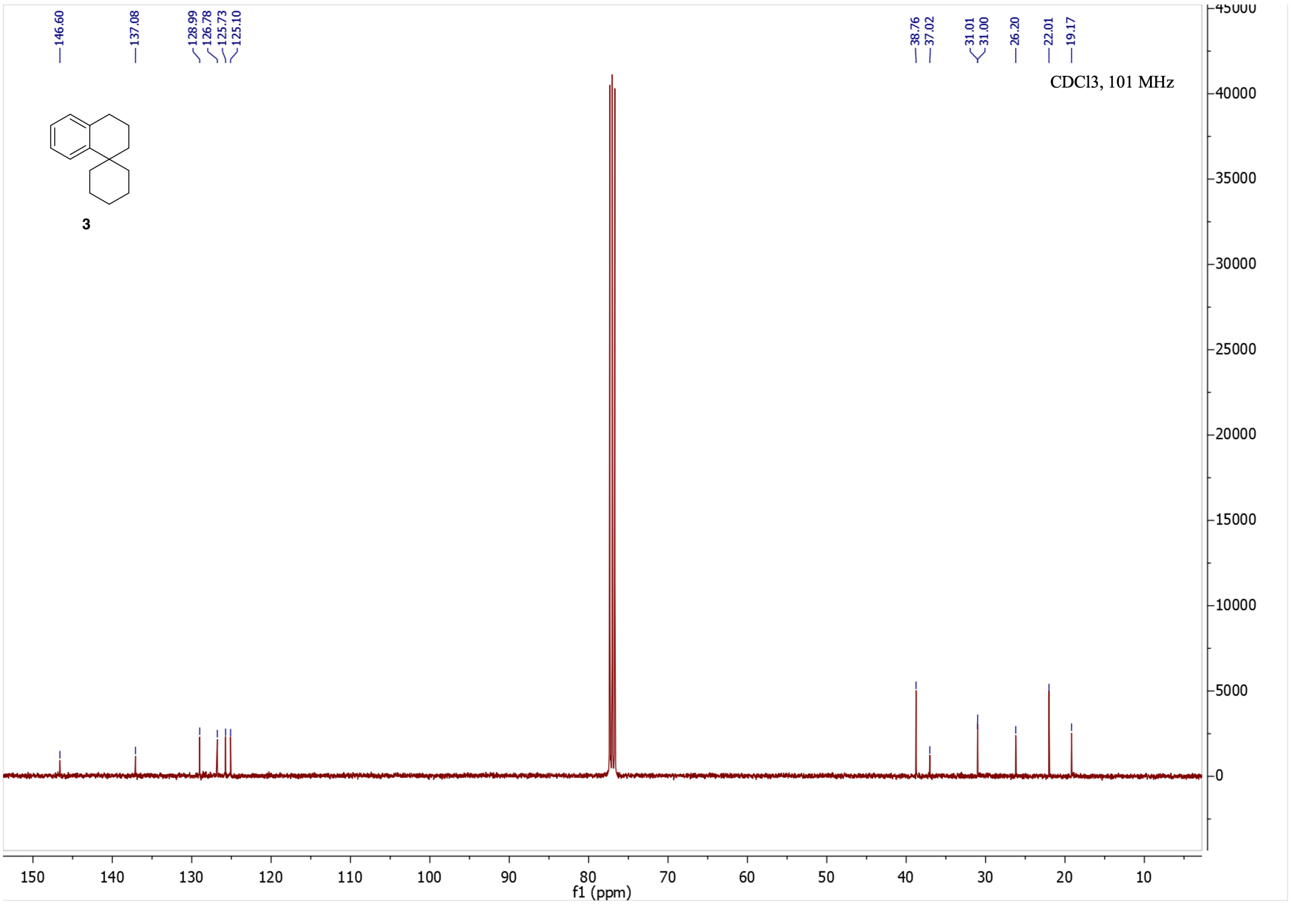
146.60
137.08
128.99
126.78
125.73
125.10
38.76
37.02
31.01
31.00
26.20
22.01
19.17
CDCl3, 101 MHz
3
f1 (ppm)

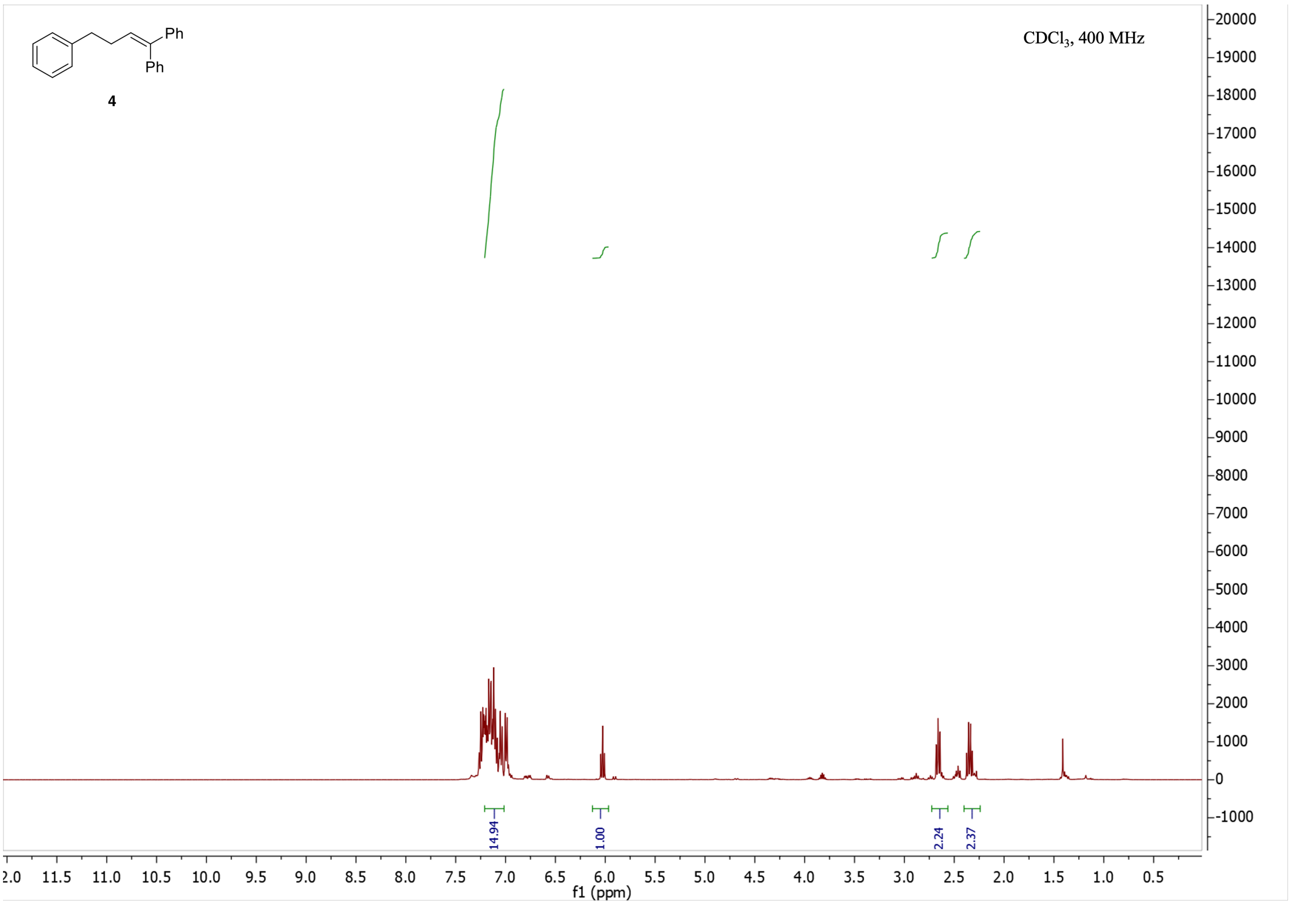
Ph
Ph
4
CDCl$_3$, 400 MHz
14.94
1.00
2.24
2.37
f1 (ppm)

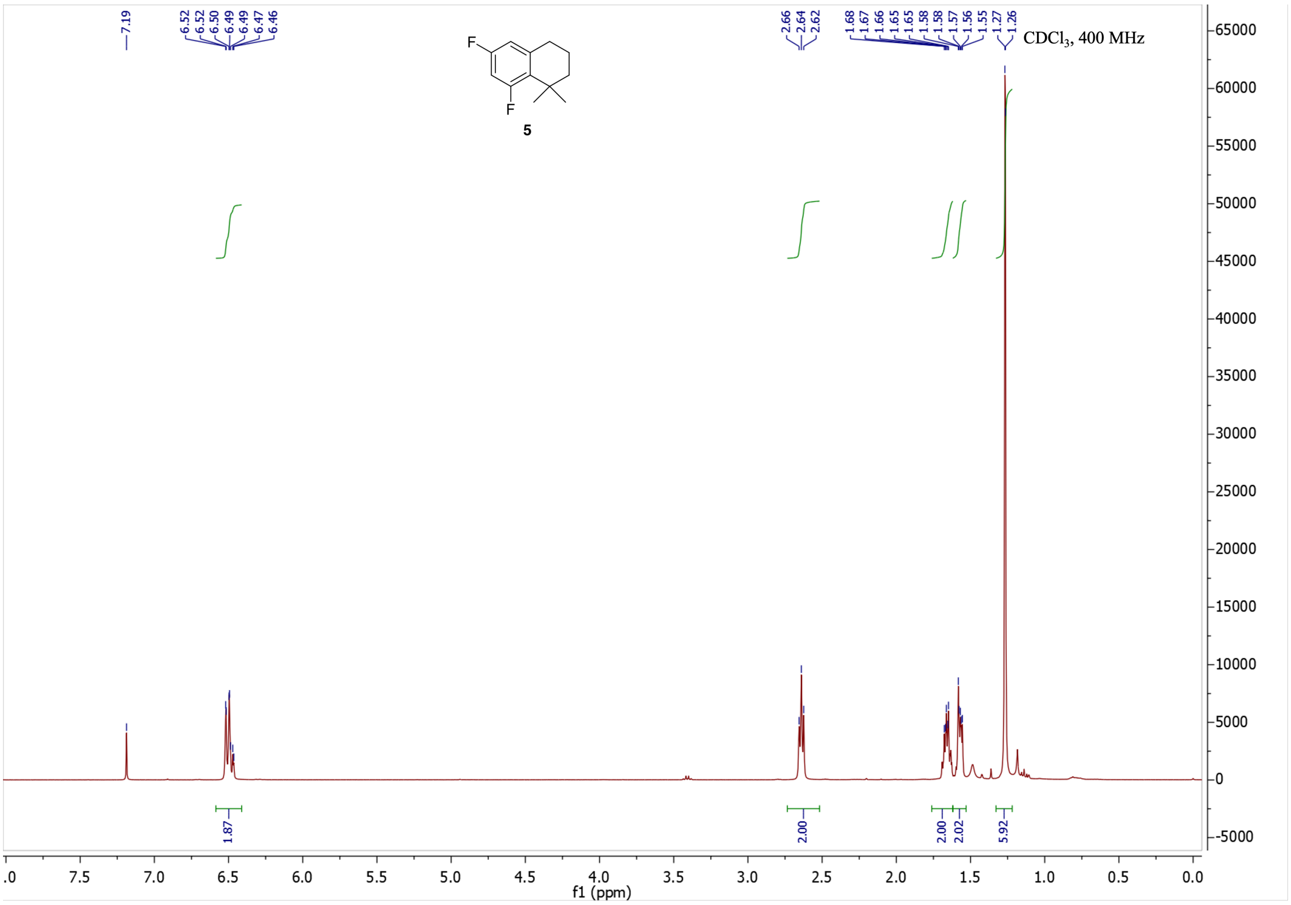
CDCl3, 400 MHz
5
F
F
7.19
6.52
6.52
6.50
6.49
6.49
6.47
6.46
2.66
2.64
2.62
1.68
1.67
1.66
1.65
1.65
1.58
1.58
1.57
1.56
1.55
1.27
1.26
1.87
2.00
2.00
2.02
5.92
f1 (ppm)

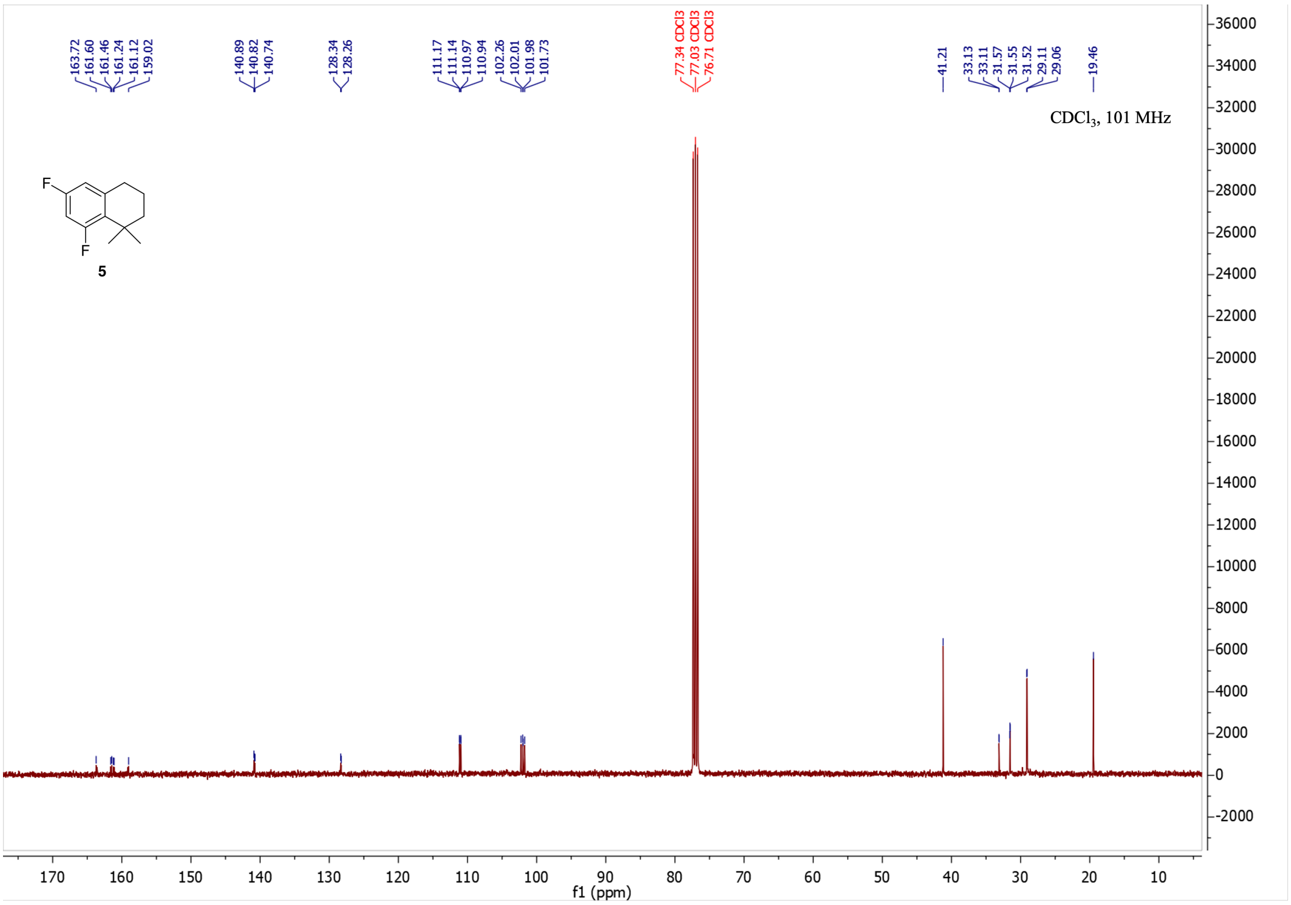
163.72
161.60
161.46
161.24
161.12
159.02
140.89
140.82
140.74
128.34
128.26
111.17
111.14
110.97
110.94
102.26
102.01
101.98
101.73
77.34 CDCl3
77.03 CDCl3
76.71 CDCl3
41.21
33.13
33.11
31.57
31.55
31.52
29.11
29.06
19.46
CDCl$_3$, 101 MHz
F
F
5
f1 (ppm)

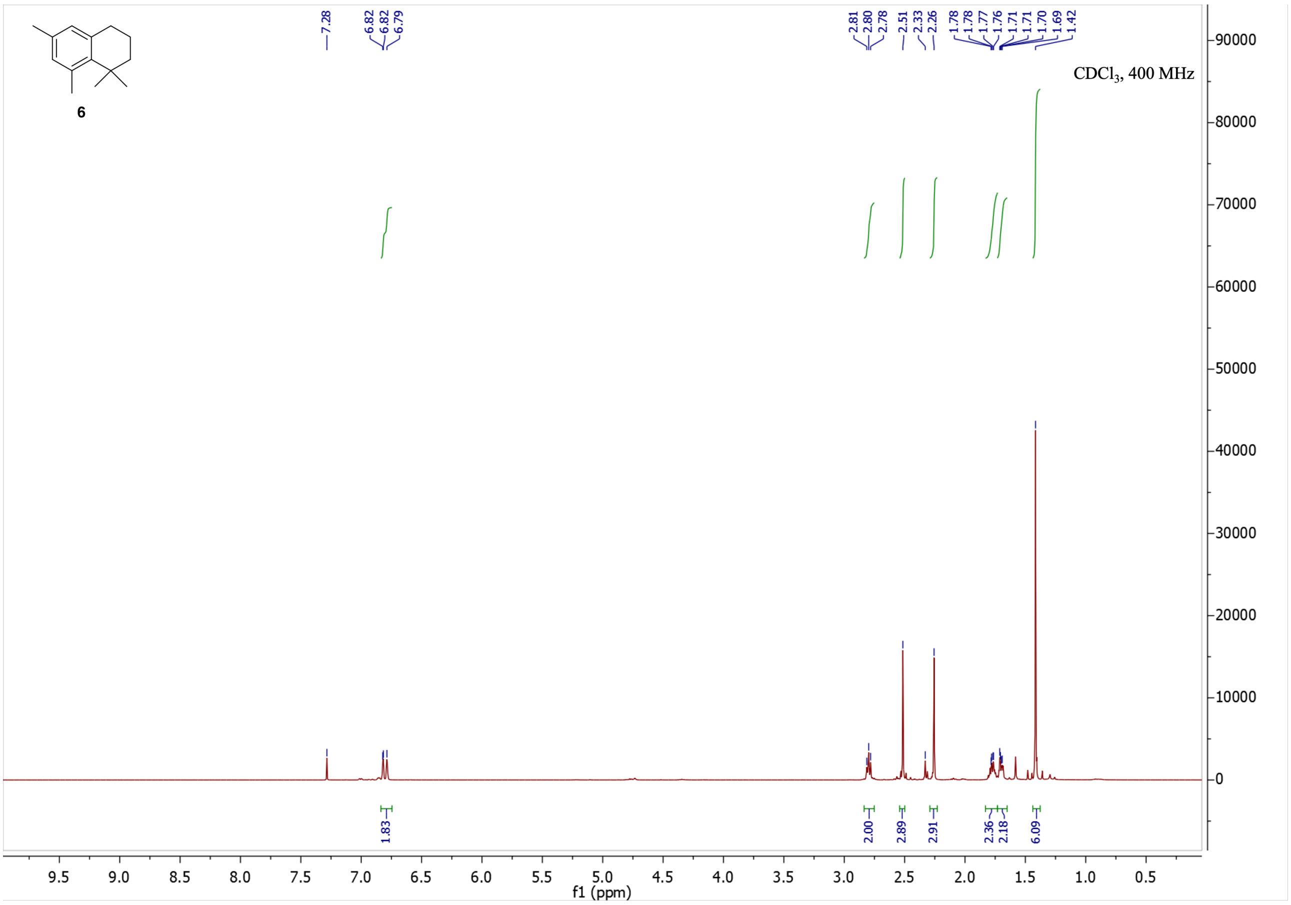
7.28
6.82
6.82
6.79
2.81
2.80
2.78
2.51
2.33
2.26
1.78
1.78
1.77
1.76
1.71
1.71
1.70
1.69
1.42
CDCl$_3$, 400 MHz
6
1.83
2.00
2.89
2.91
2.36
2.18
6.09
f1 (ppm)

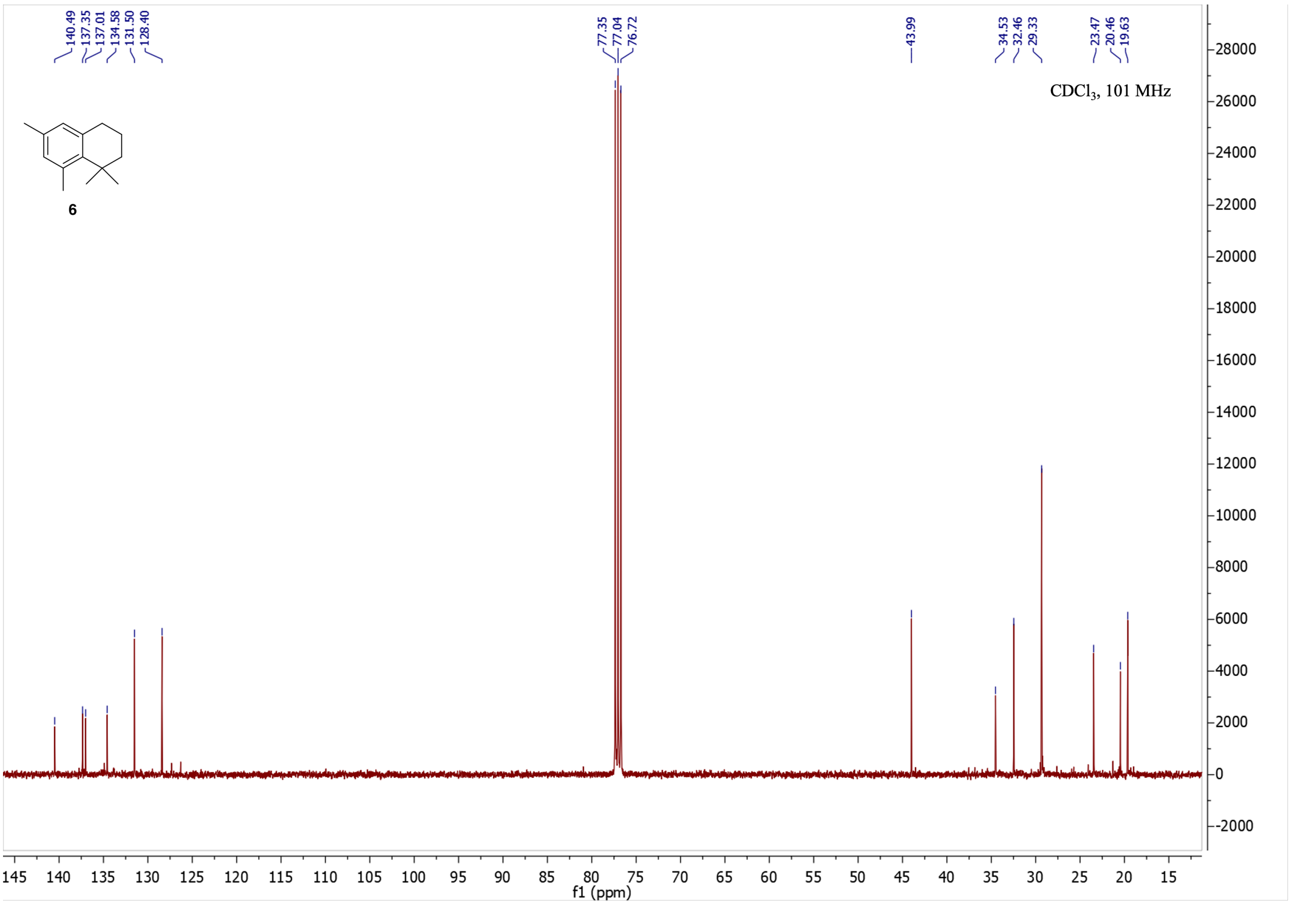
140.49
137.35
137.01
134.58
131.50
128.40
77.35
77.04
76.72
43.99
34.53
32.46
29.33
23.47
20.46
19.63
CDCl3, 101 MHz
6
f1 (ppm)

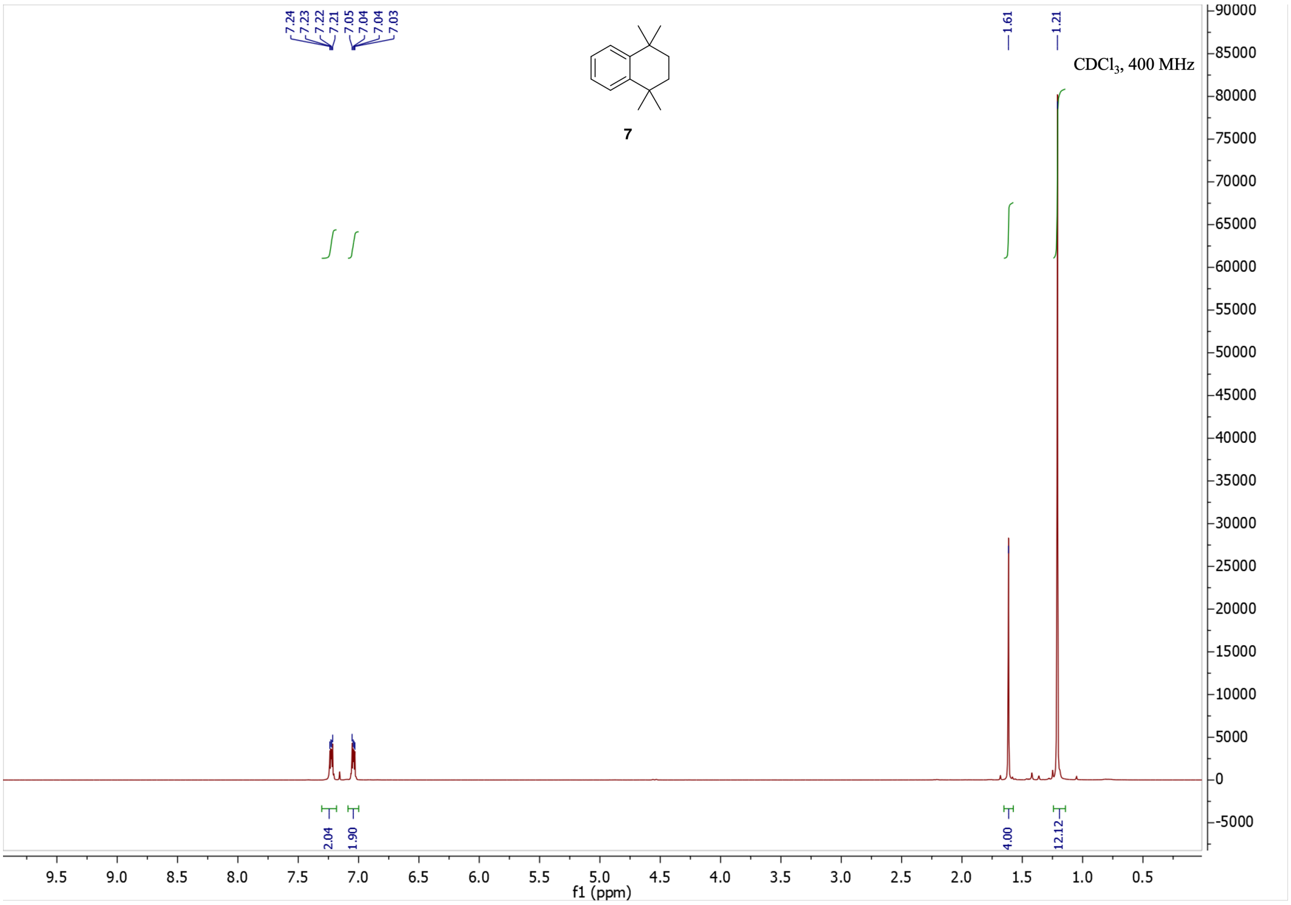
7.24
7.23
7.22
7.21
7.05
7.04
7.04
7.03
1.61
1.21
CDCl$_3$, 400 MHz
7
2.04
1.90
4.00
12.12
f1 (ppm)

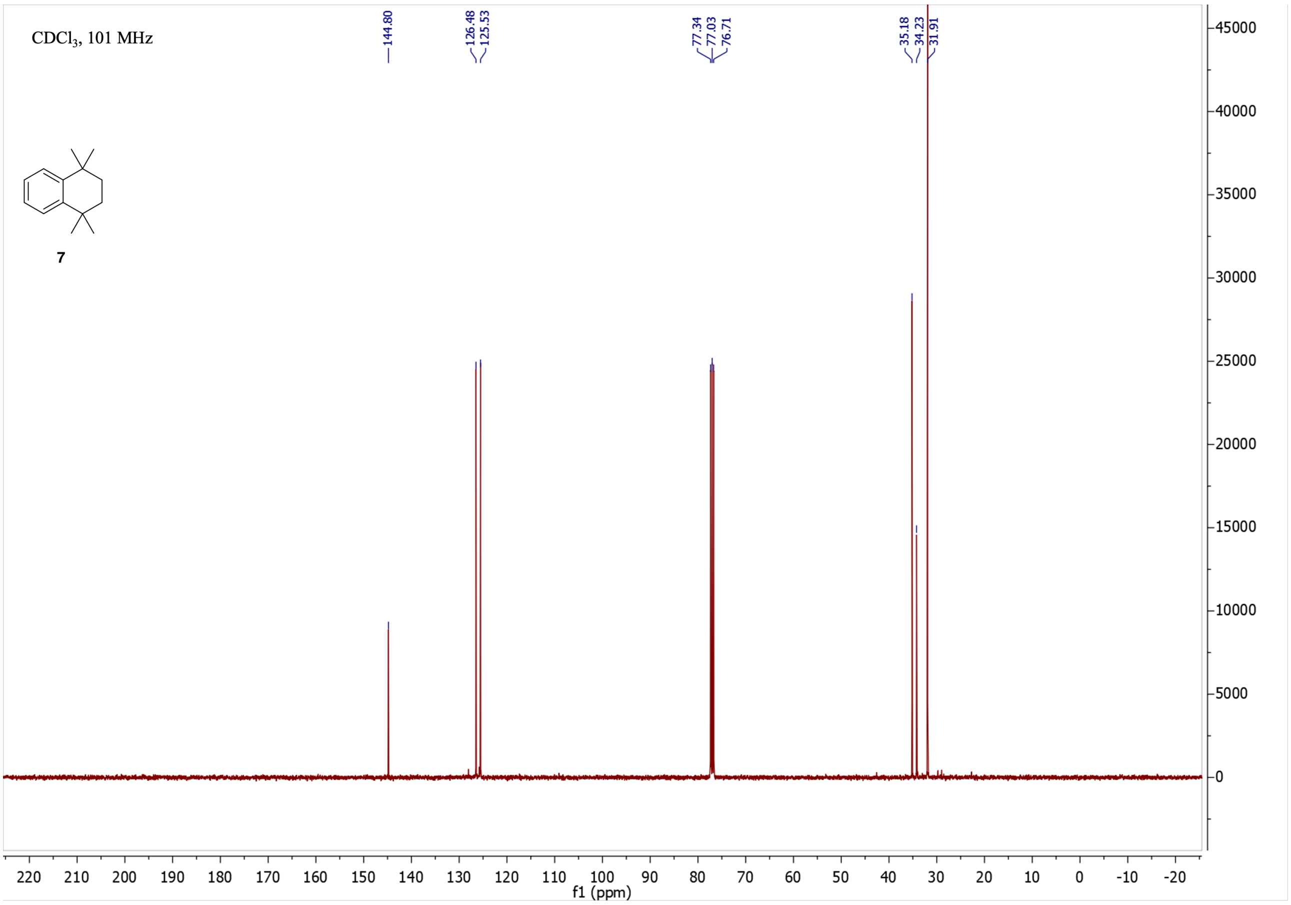
CDCl3, 101 MHz
7
144.80
126.48
125.53
77.34
77.03
76.71
35.18
34.23
31.91
45000
40000
35000
30000
25000
20000
15000
10000
5000
0
220 210 200 190 180 170 160 150 140 130 120 110 100 90 80 70 60 50 40 30 20 10 0 -10 -20
f1 (ppm)

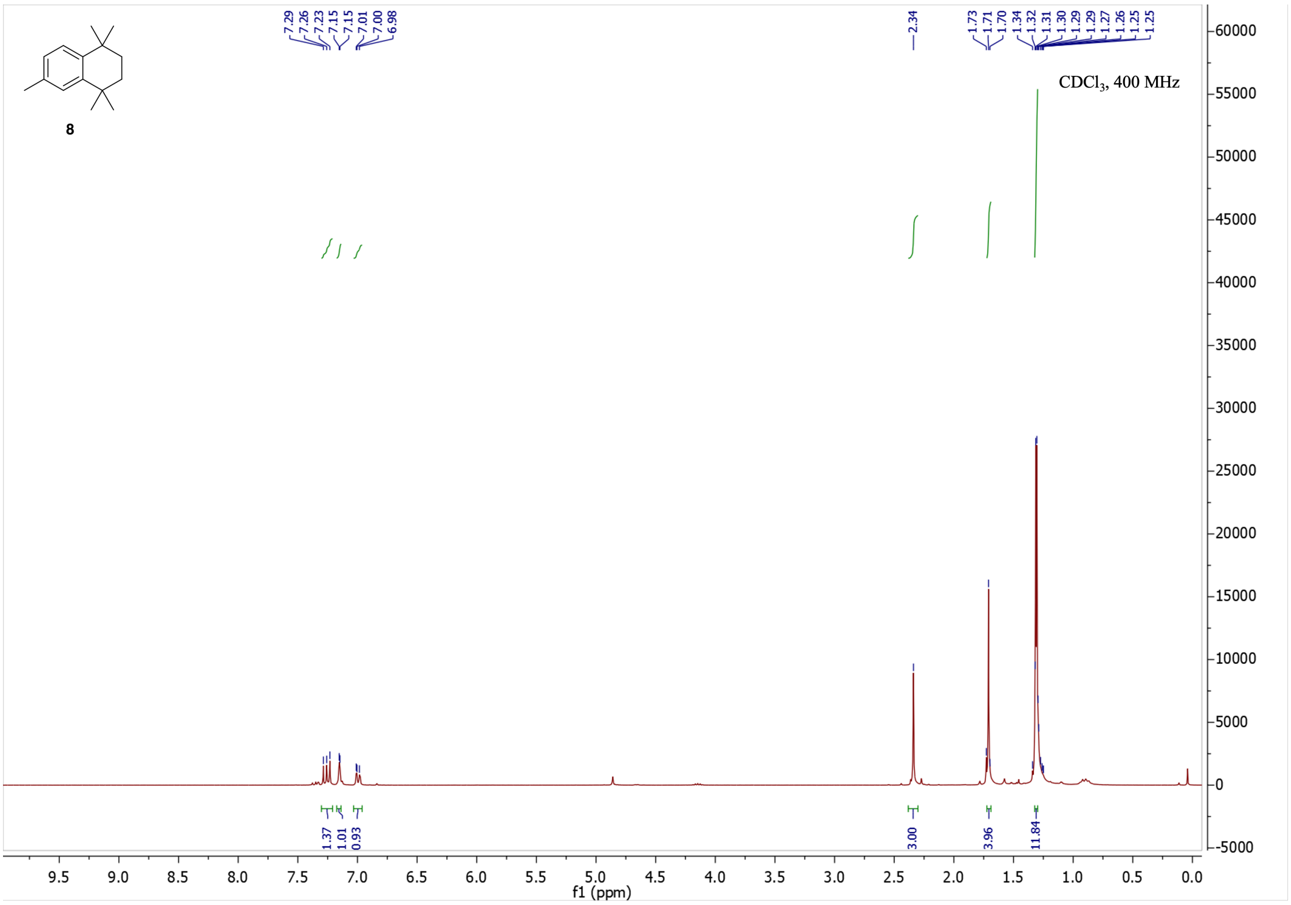
8
CDCl3, 400 MHz
7.29
7.26
7.23
7.15
7.15
7.01
7.00
6.98
2.34
1.73
1.71
1.70
1.34
1.32
1.31
1.30
1.29
1.29
1.27
1.26
1.25
1.25
1.37
1.01
0.93
3.00
3.96
11.84
f1 (ppm)

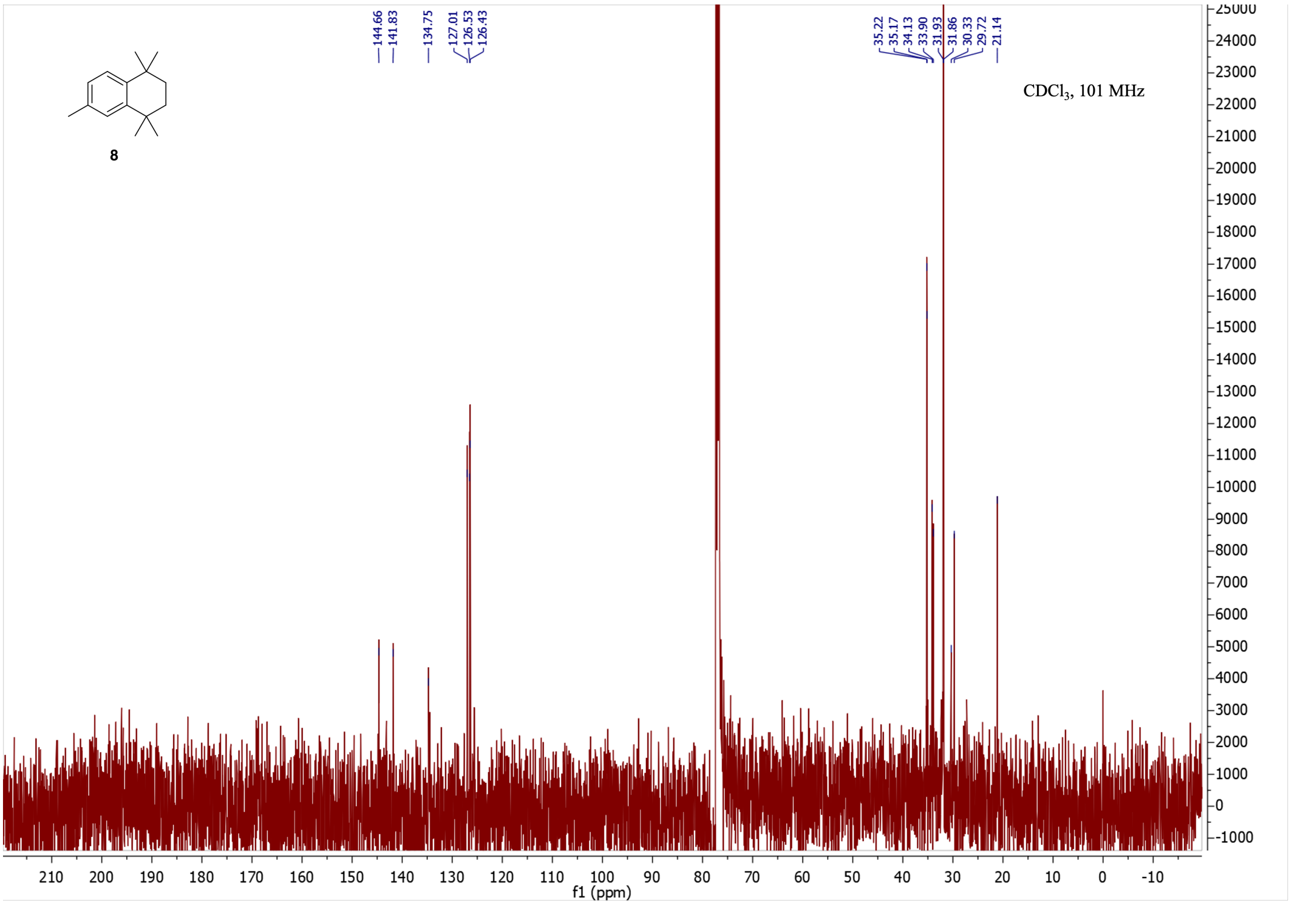
8
CDCl3, 101 MHz
144.66
141.83
134.75
127.01
126.53
126.43
35.22
35.17
34.13
33.90
31.93
31.86
30.33
29.72
21.14
f1 (ppm)

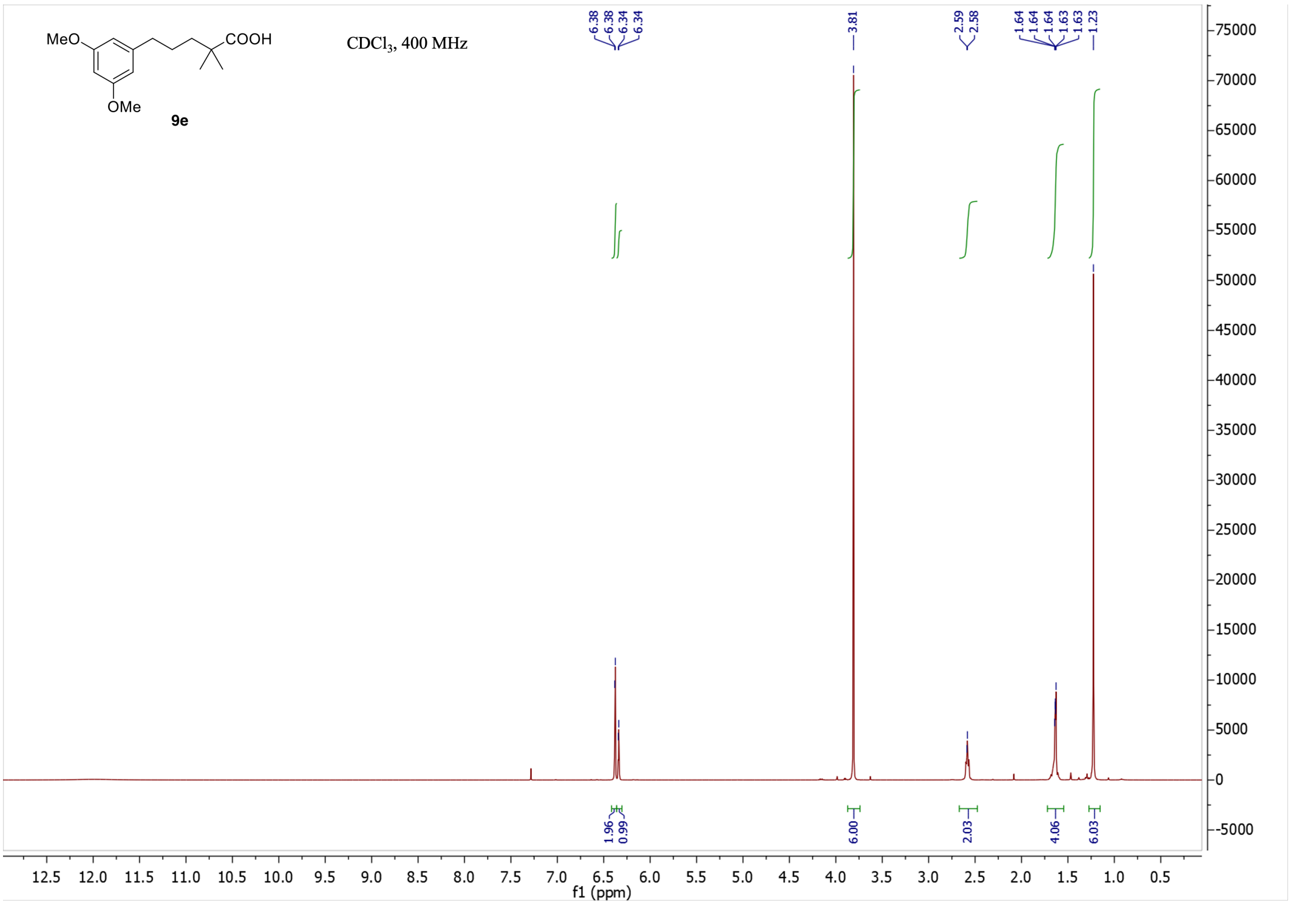
MeO
COOH
OMe
9e
CDCl3, 400 MHz
6.38
6.38
6.34
6.34
3.81
2.59
2.58
1.64
1.64
1.64
1.63
1.63
1.23
1.96
0.99
6.00
2.03
4.06
6.03
f1 (ppm)

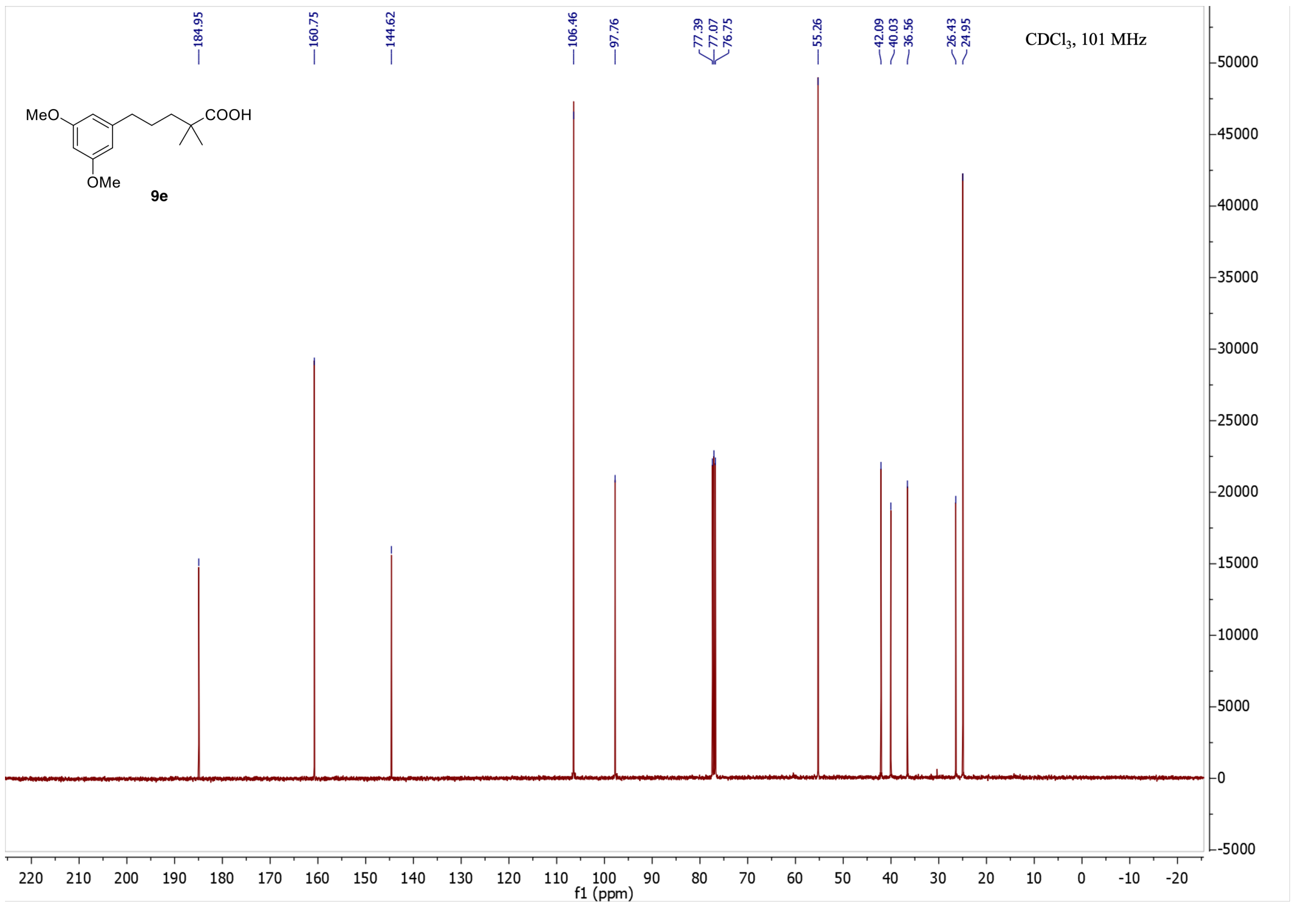
184.95
160.75
144.62
106.46
97.76
77.39
77.07
76.75
55.26
42.09
40.03
36.56
26.43
24.95
CDCl3, 101 MHz
MeO
COOH
OMe
9e
f1 (ppm)

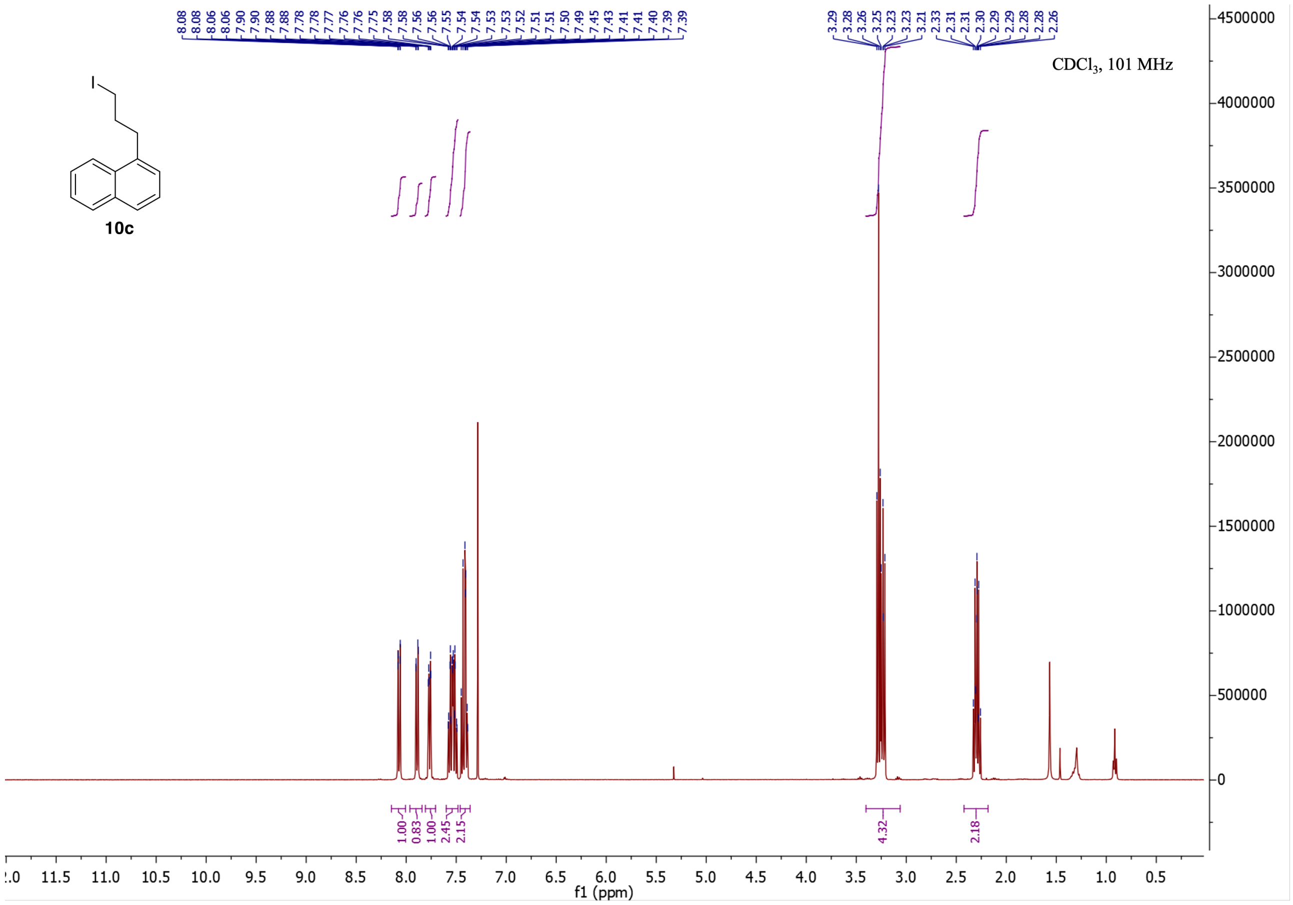

10c

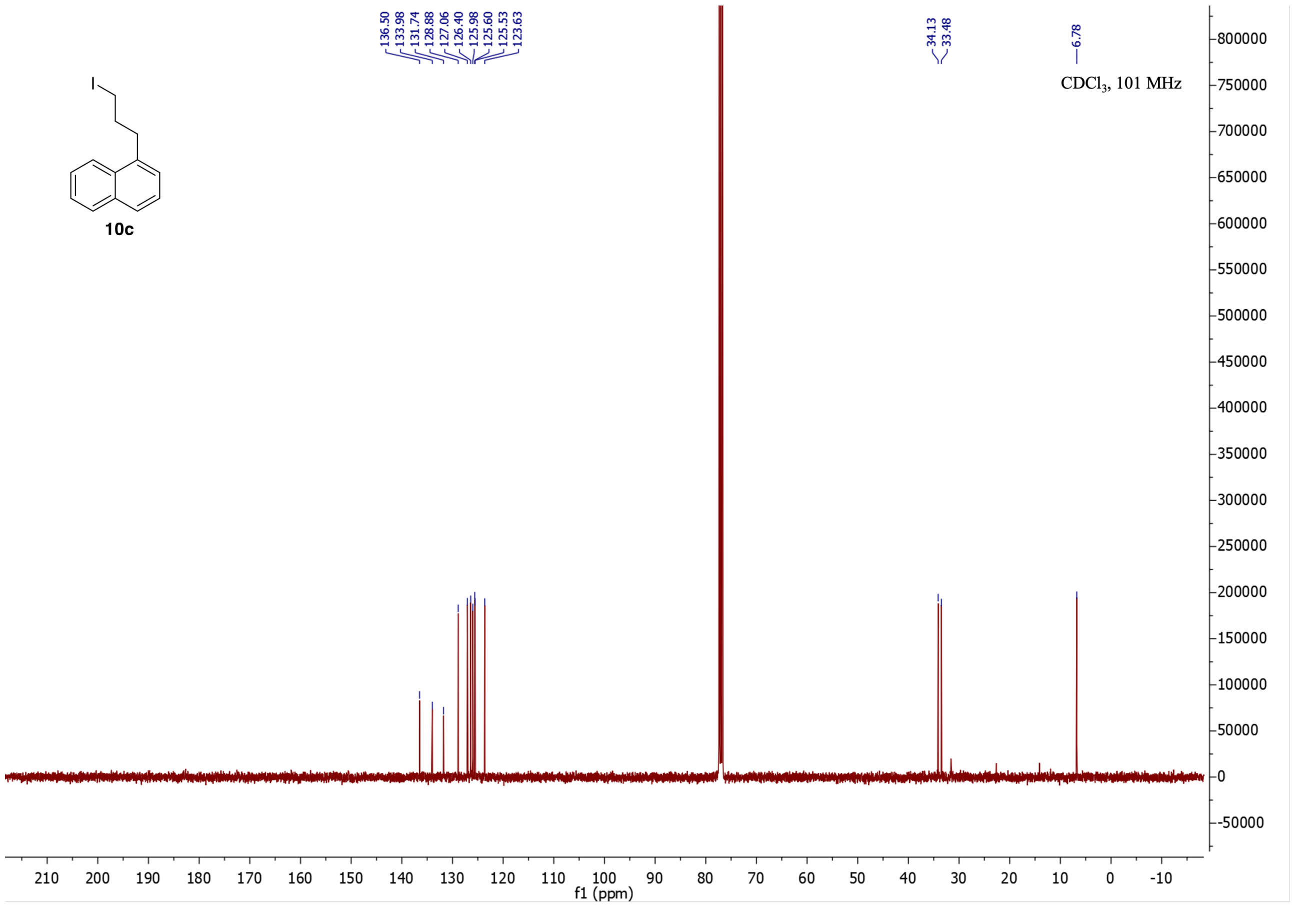

10c
CDCl3, 101 MHz
136.50
133.98
131.74
128.88
127.06
126.40
125.98
125.60
125.53
123.63
34.13
33.48
6.78
f1 (ppm)

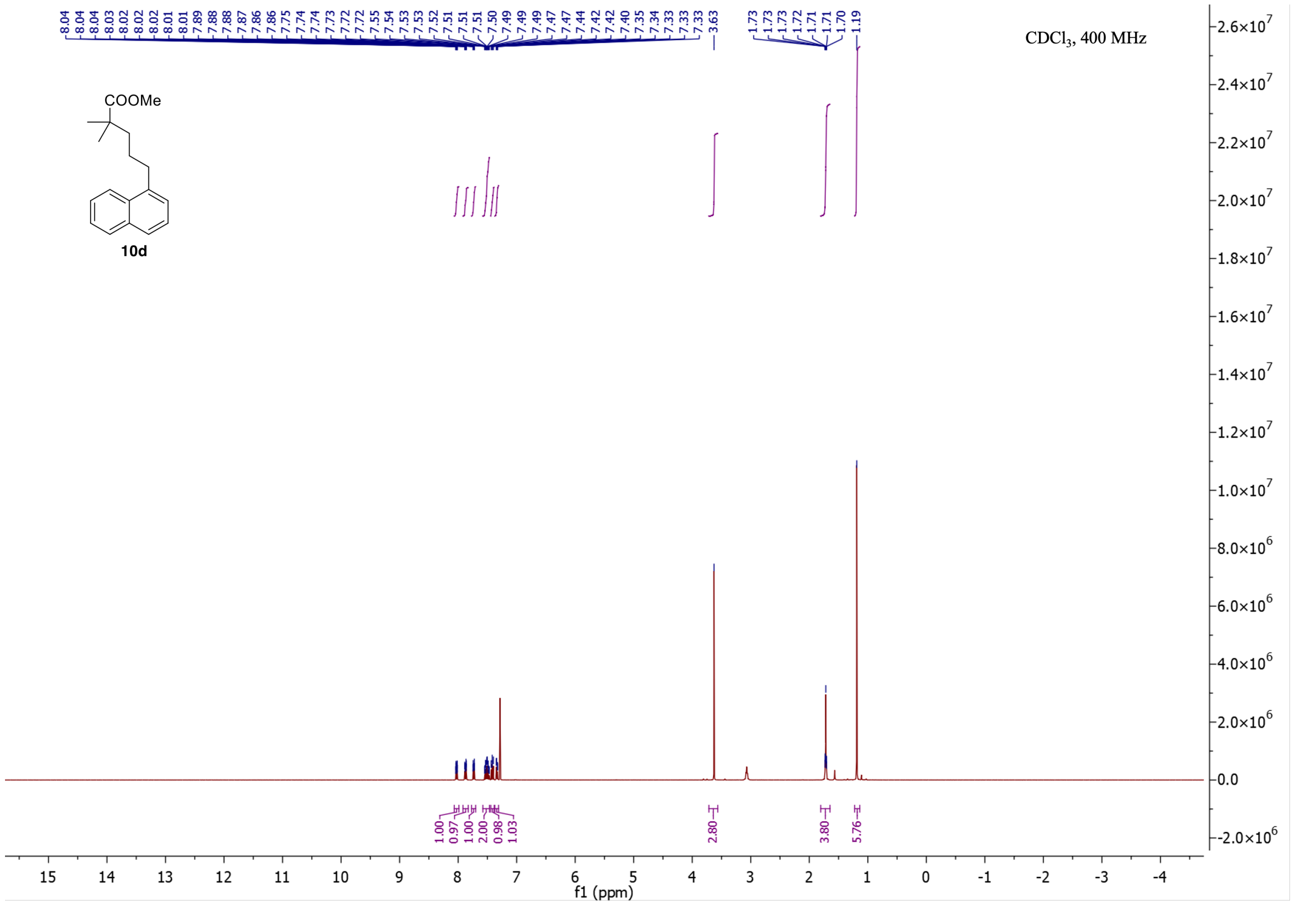

COOMe
10d
CDCl3, 400 MHz
3.63
1.19
2.80
3.80
5.76
f1 (ppm)

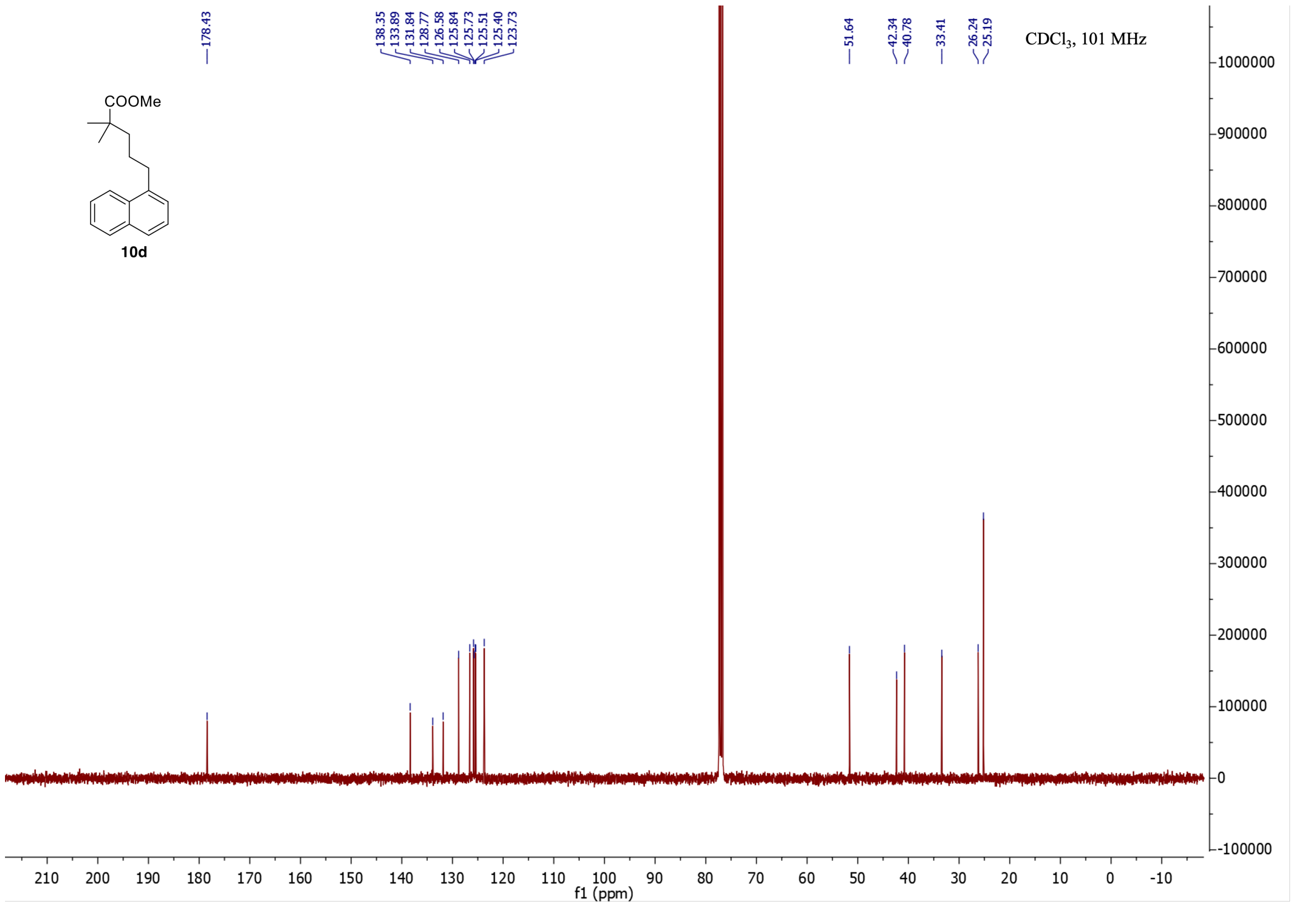

178.43
138.35
133.89
131.84
128.77
126.58
125.84
125.73
125.51
125.40
123.73
51.64
42.34
40.78
33.41
26.24
25.19
CDCl3, 101 MHz
COOMe
10d
f1 (ppm)

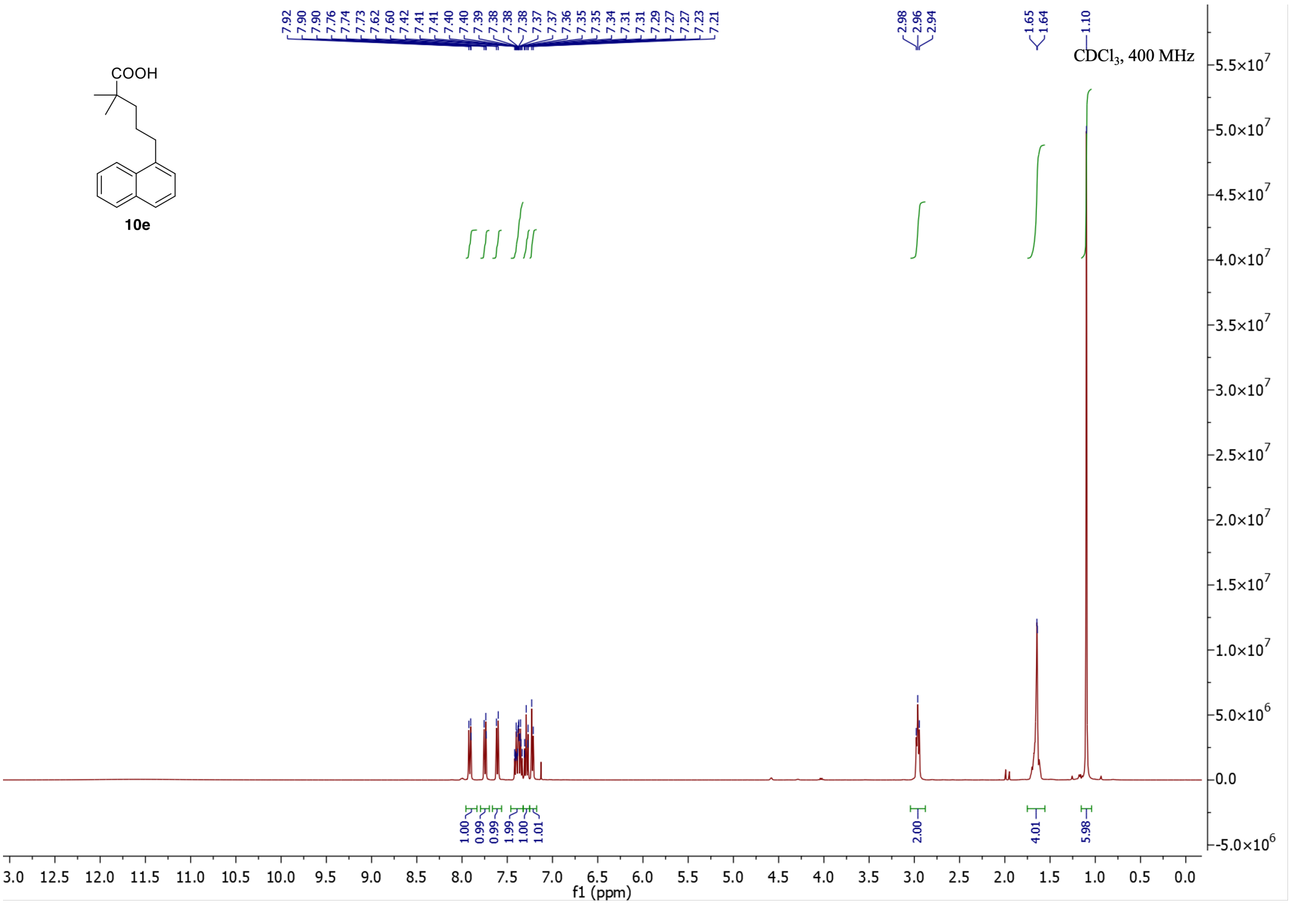

2.98
2.96
2.94
1.65
1.64
1.10
CDCl3, 400 MHz
COOH
10e
1.00
0.99
0.99
1.99
1.00
1.01
2.00
4.01
5.98
f1 (ppm)

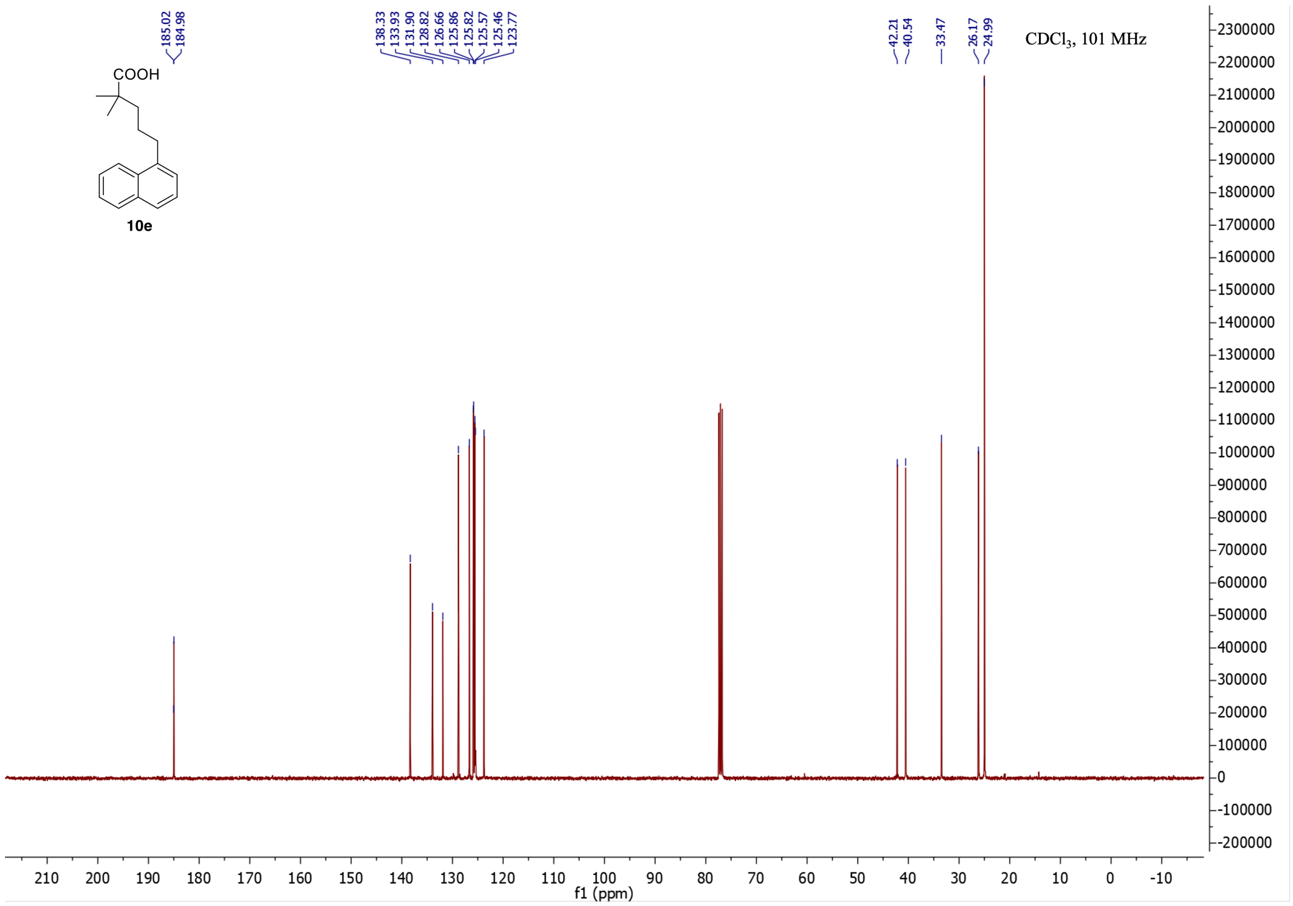
185.02
184.98
138.33
133.93
131.90
128.82
126.66
125.86
125.82
125.57
125.46
123.77
42.21
40.54
33.47
26.17
24.99
CDCl3, 101 MHz
COOH
10e
f1 (ppm)

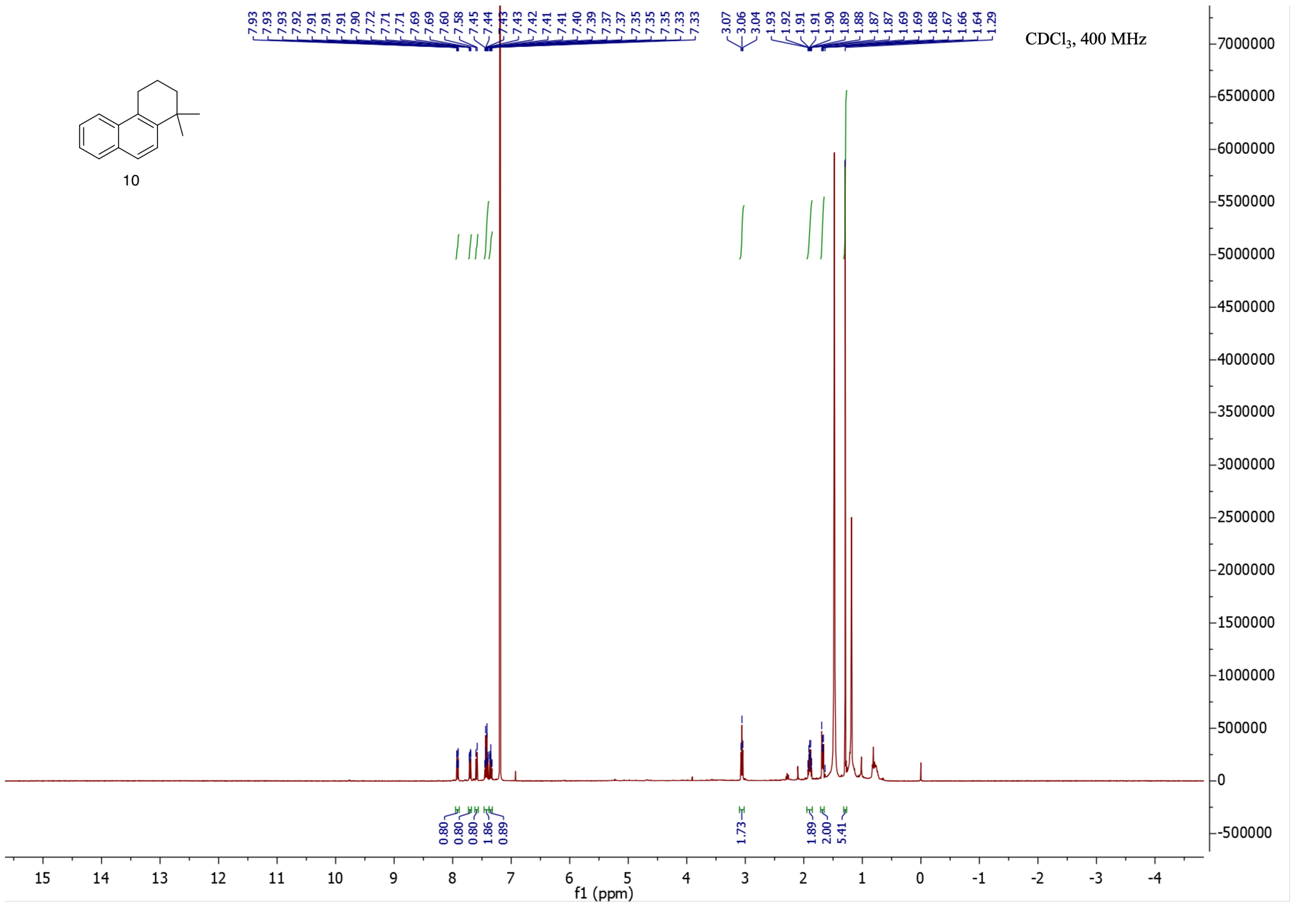

10
CDCl3, 400 MHz
f1 (ppm)

Cartesian coordinates

B

M06-2X/6-31+g(d,p),

el. energy = -467.127663 a.u.

C	3.97729700	-0.01013600	-0.77388500
C	3.38518900	1.19249700	-0.38611500
C	2.15734800	1.18601500	0.27547100
C	1.50580000	-0.01932900	0.56038400
C	2.10763900	-1.21962100	0.16577700
C	3.33528600	-1.21748000	-0.49584300
H	4.93486700	-0.00677100	-1.28576200
H	3.88188900	2.13560000	-0.59454600
H	1.70118500	2.12491300	0.58168800
H	1.61218900	-2.16269000	0.38625400
H	3.79272700	-2.15774600	-0.78950700
C	0.15771200	-0.02581800	1.23904000
H	0.03638100	-0.94703100	1.81833700
H	0.07765600	0.81947100	1.93068800
C	-0.96839600	0.06669300	0.19958500
H	-0.89290600	-0.76734300	-0.50568200
H	-0.88449800	1.00071300	-0.36432000
C	-2.35854800	0.01944400	0.93452000
H	-2.40490300	-0.89823100	1.52578300
H	-2.44458800	0.91349100	1.55690700
C	-3.33320200	0.01707500	-0.14509700
C	-3.86344000	-1.25106300	-0.63479800
H	-3.16089800	-2.07494200	-0.48713000
H	-4.71549400	-1.44852600	0.04391500
H	-4.25289000	-1.19494700	-1.65172200
C	-3.73347200	1.26334000	-0.79319500
H	-3.44164700	2.15693400	-0.24370100
H	-3.24679300	1.25569200	-1.78371700
H	-4.80756000	1.24171100	-1.01254900

Py

M06-2X/6-31+g(d,p),

el. energy = -248.197867 a.u.

C	1.14383500	-0.72117000	0.00006200
C	1.19750900	0.67155600	-0.00005500
C	-0.00010400	1.38227000	0.00000700
C	-1.19759300	0.67139000	0.00002700
C	-1.14374500	-0.72132900	-0.00009300
N	0.00010800	-1.41582000	0.00002000
H	-0.00021000	2.46821200	0.00004900
H	2.06045400	-1.30593200	-0.00000600
H	2.15618500	1.17936800	-0.00004100
H	-2.15635000	1.17906600	0.00010900
H	-2.06025000	-1.30626700	0.00006500

TS-conf

M06-2X/6-31+g(d,p),

el. energy = -467.120942 a.u.

im. frequency -47.31

C	3.45401100	0.80593700	0.39120500
C	3.26926300	-0.56476300	0.58656700
C	2.10490700	-1.18492600	0.13779700
C	1.10598800	-0.44810300	-0.51179800
C	1.30181300	0.92304900	-0.70310600
C	2.46766600	1.54852400	-0.25579400
H	4.36283100	1.28891600	0.73715100
H	4.03611200	-1.15091800	1.08458900
H	1.96810600	-2.25376100	0.28843600
H	0.53741700	1.50498300	-1.21466600
H	2.60592000	2.61353800	-0.41812700
C	-0.17057100	-1.12054700	-0.96099300
H	-0.69869400	-0.44616900	-1.64308200

H	0.07200800	-2.01471300	-1.54359200
C	-1.07723000	-1.53136800	0.22428000
H	-0.68812500	-1.14022900	1.16855600
H	-1.11267500	-2.61809500	0.32293900
C	-2.57320700	-1.08649200	0.06754100
H	-2.95412900	-1.40038900	-0.90653300
H	-3.13349100	-1.55966200	0.88139000
C	-2.64889400	0.36487400	0.23663400
C	-2.92669700	1.23143300	-0.89876400
H	-2.62008000	0.79794200	-1.85304800
H	-4.03649000	1.23912300	-0.91531600
H	-2.59734500	2.26144200	-0.75484500
C	-2.43116000	0.96389200	1.55135400
H	-2.47710000	0.24952000	2.37249700
H	-1.41777300	1.40212200	1.50552300
H	-3.10182000	1.81727900	1.69858100

INT-1

M06-2X/6-31+g(d,p),

el. energy = -467.133408 a.u.

C	-2.60135200	-0.84049400	0.04689500
C	-2.47531600	0.28714500	0.86871600
C	-1.48457200	1.23052500	0.61646600
C	-0.60059600	1.06820900	-0.45985600
C	-0.74655800	-0.05476600	-1.28581000
C	-1.74222300	-1.00486900	-1.03588500
H	-3.37601000	-1.57455300	0.24636500
H	-3.15413900	0.42496900	1.70518400
H	-1.38546200	2.10088200	1.26118000
H	-0.08488600	-0.17406000	-2.14157600
H	-1.84180500	-1.86744400	-1.68834300
C	0.54571800	2.02178600	-0.66042400
H	0.85667100	2.02145200	-1.71054400
H	0.24186300	3.04079200	-0.40139100
C	1.74302100	1.63037900	0.22326000
H	1.46740500	1.70243900	1.28008500
H	2.56665500	2.32914400	0.05529200
C	2.24982400	0.22287500	-0.10924800
H	2.53479300	0.13082300	-1.16116900
H	3.17207900	0.04071500	0.48445200
C	1.46527000	-0.94155500	0.31929700
C	1.53886400	-2.18119400	-0.46834300
H	1.59180200	-1.99707700	-1.54170400
H	2.50149200	-2.63330000	-0.16866200
H	0.75116400	-2.88929100	-0.20654800
C	0.81951400	-1.00880700	1.63254000
H	0.94200000	-0.11574300	2.24123500
H	-0.25123500	-1.21412800	1.47020300
H	1.19804300	-1.90102700	2.15028500

INT-1–Py Adduct

M06-2X/6-31+g(d,p),

el. energy = -715.337504 a.u.

C	3.17684700	-1.69293000	-1.36404100
C	3.86197100	-1.40993500	-0.17525200
C	3.16636400	-0.97001300	0.94642100
C	1.77516500	-0.80084100	0.90336900
C	1.09719900	-1.09861500	-0.28658500
C	1.79364400	-1.54551900	-1.41450300
H	3.72290200	-2.03670300	-2.23730600
H	4.93972000	-1.53594000	-0.12894500
H	3.70265500	-0.74559100	1.86584400
H	0.01582400	-0.97626600	-0.32694200
H	1.25175700	-1.77197000	-2.32842400
C	1.03767700	-0.20941000	2.07439400
H	-0.01078000	-0.52777900	2.05883300
H	1.48036000	-0.55330400	3.01449700
C	1.09725700	1.32754900	2.03761900
H	2.13643800	1.66413100	2.10885200
H	0.56384600	1.73569500	2.90018000

C	0.43079700	1.87995500	0.77330800
H	-0.60595400	1.54103200	0.68247200
H	0.40436600	2.98840400	0.85029600
C	1.12222000	1.74682700	-0.51573900
C	-2.74792700	-1.30158300	0.18633000
C	-4.09292300	-1.59694100	0.40017100
C	-5.04204900	-0.62040600	0.10861000
C	-4.60791800	0.60820300	-0.38358600
C	-3.24030900	0.80519200	-0.56207300
N	-2.31858500	-0.12489200	-0.28544600
H	-6.09958500	-0.81313400	0.26164600
H	-1.98072600	-2.04279400	0.40247200
H	-4.38177400	-2.56910700	0.78501400
H	-5.30833100	1.40022600	-0.62595800
H	-2.86779500	1.75410600	-0.94433100
C	0.31040700	1.64163800	-1.73643600
H	-0.54302200	0.97253400	-1.59191300
H	-0.10060300	2.65672700	-1.88103100
H	0.90234500	1.38821900	-2.61667500
C	2.56485000	1.95993900	-0.65828200
H	2.99818600	1.06206000	-1.12821000
H	2.70984700	2.76006000	-1.39760900
H	3.08079000	2.18497600	0.27300700

TS-cycl

M06-2X/6-31+g(d,p),

el. energy = -715.334118 a.u.

im. frequency -178.50

C	-2.93995600	1.82702800	-1.24265200
C	-3.67309900	1.62276500	-0.05820200
C	-3.08465600	1.03910400	1.05734100
C	-1.74540200	0.63438300	1.01635700
C	-1.02379000	0.78717400	-0.19039600
C	-1.61948900	1.42744400	-1.30188000
H	-3.41219000	2.30449200	-2.09502700
H	-4.71237500	1.93542000	-0.01348400
H	-3.65898500	0.89841200	1.96902600
H	0.05145100	0.61024800	-0.18866600
H	-1.03298300	1.58617100	-2.20222900
C	-1.09575800	-0.07605800	2.16219000
H	-0.04846600	0.23271800	2.24929200
H	-1.60723600	0.15803500	3.09966800
C	-1.15034300	-1.59738800	1.90792200
H	-2.18540800	-1.94778000	1.97192500
H	-0.58396100	-2.11732100	2.68503400
C	-0.54789000	-1.93220200	0.54897700
H	0.51044400	-1.65413800	0.50578900
H	-0.60226400	-3.02411100	0.38906900
C	-1.24647300	-1.41183500	-0.66453400
C	2.75373700	1.30456600	0.18454600
C	4.10051800	1.57956600	0.41331800
C	5.03393200	0.57615300	0.16562100
C	4.58329400	-0.65720300	-0.29939000
C	3.21531300	-0.83277600	-0.49632100
N	2.30923100	0.12386700	-0.26171600
H	6.09247800	0.75180600	0.33175300
H	1.99738400	2.06567900	0.36744600
H	4.40282500	2.55625500	0.77580200
H	5.27201100	-1.46915400	-0.50697300
H	2.82903700	-1.78372400	-0.85873900
C	-0.44433900	-1.39870500	-1.92390600
H	0.52439600	-0.91263100	-1.78794300
H	-0.25765100	-2.45865400	-2.15489600
H	-0.99202200	-0.96090400	-2.75930200
C	-2.70648500	-1.63461700	-0.82022400
H	-3.16275300	-0.88855300	-1.47675700
H	-2.79304900	-2.59984300	-1.34229500
H	-3.24998900	-1.69388300	0.12091200

2

M06-2X/6-31+g(d,p),

el. energy = -466.754896 a.u.

C	-2.50351100	-1.26278000	0.08077800
C	-3.02548700	0.03074200	0.02011400
C	-2.15387700	1.11089200	-0.05612400
C	-0.76244400	0.93419800	-0.08250500
C	-0.23229800	-0.36808500	-0.03544600
C	-1.12526200	-1.44861600	0.05629300
H	-3.16594200	-2.12083700	0.14933000
H	-4.09906000	0.19417300	0.04004200
H	-2.55079400	2.12350300	-0.09133300
H	-0.73286300	-2.46066100	0.11301200
C	0.12443800	2.15905900	-0.16728000
H	0.15801000	2.50089000	-1.21139700
H	-0.32954500	2.97127400	0.41059500
C	1.54599900	1.87483900	0.30540700
H	1.55531000	1.72677200	1.39238500
H	2.19096000	2.73367200	0.09292800
C	2.06692200	0.62915200	-0.40295200
H	2.00060200	0.79433000	-1.48808100
H	3.12489900	0.45522500	-0.17190000
C	1.27623800	-0.64302500	-0.04296800
C	1.62808300	-1.72384100	-1.07780700
H	1.26439600	-1.44720200	-2.07331700
H	2.71704700	-1.83855900	-1.13180000
H	1.20603700	-2.69901900	-0.81709100
C	1.69979200	-1.14380000	1.35005700
H	1.15453800	-2.05479300	1.61866100
H	2.77159200	-1.37443900	1.35682300
H	1.50325300	-0.39674000	2.12573600

Py-H

M06-2X/6-31+g(d,p),

el. energy = -248.643935 a.u.

C	-0.66698100	-1.18402200	0.00000400
C	0.71490100	-1.20876800	-0.00000100
C	1.41179000	-0.00058100	-0.00000400
C	0.71591500	1.20819200	0.00000400
C	-0.66599200	1.18456500	-0.00000400
N	-1.30204800	0.00053300	0.00000300
H	2.49691600	-0.00104100	-0.00000700
H	-1.29108800	-2.06881700	0.00000800
H	1.22989200	-2.16139000	0.00000000
H	1.23168500	2.16039100	0.00001100
H	-1.28938500	2.06987800	0.00000300
H	-2.32148600	0.00093300	-0.00003000

TS-elim

M06-2X/6-31+g(d,p),

el. energy = -715.332160 a.u.

im. frequency -8.13

C	4.82248100	-0.56115500	0.51496400
C	3.69381300	-1.30325400	0.86521100
C	2.44130900	-0.94954300	0.36351900
C	2.29633100	0.14722300	-0.49374900
C	3.43509800	0.88374200	-0.83884900
C	4.68990400	0.53382400	-0.33969600
H	5.79898200	-0.83734900	0.90129200
H	3.78908100	-2.16041200	1.52550500
H	1.56390000	-1.53406200	0.63363900
H	3.33722200	1.73467700	-1.50966900
H	5.56449300	1.11293000	-0.62169500
C	0.93184900	0.55230300	-0.99563100
H	1.01780000	0.99098100	-1.99648000
H	0.29279000	-0.33449500	-1.07847900
C	0.26133000	1.56647700	-0.05921500
H	0.86849600	2.47878300	-0.00936000
H	0.21687900	1.15132800	0.95419400
C	-1.14212900	1.90706400	-0.54440900

H	-1.22690200	2.08724000	-1.62217400
H	-1.79411200	0.98154400	-0.37698300
C	-1.96551700	2.80781600	0.20953400
C	-3.15331800	3.40384600	-0.40783200
H	-3.38611800	2.98770600	-1.38782200
H	-4.00752800	3.36579100	0.27557300
H	-2.90957300	4.47472100	-0.51828500
C	-1.66177900	3.09895600	1.61619900
H	-2.39530100	3.75241500	2.08532400
H	-1.59132000	2.14671000	2.16130100
H	-0.65439000	3.53479300	1.67107500
C	-2.36406700	-1.63979300	-1.15611600
C	-2.33966300	-3.03164600	-1.19149300
C	-2.16728700	-3.72316000	0.00542900
C	-2.02898600	-2.99711400	1.18598400
C	-2.06871900	-1.60618400	1.11981300
N	-2.23289100	-0.93463800	-0.02607000
H	-2.14109500	-4.80853700	0.01774200
H	-2.48880600	-1.06516200	-2.07144300
H	-2.45182700	-3.55375100	-2.13551600
H	-1.89237200	-3.49221200	2.14133900
H	-1.96396400	-1.00758200	2.02144600
C	3.64522900	-0.97488200	-0.16445400
H	5.13440800	0.54681600	-0.50960000
H	3.54915900	2.42155300	-0.11649800
H	1.18383800	1.94982700	0.44936900
H	1.96576000	-2.26378200	0.23465600
H	4.33220700	-1.79969100	-0.33128500
C	-0.04319200	-0.47019600	0.67275600
H	-0.13865500	-1.41645800	1.21793300
H	-0.45292100	0.31865300	1.31450400
C	-0.88886300	-0.54468600	-0.61598300
H	-0.47240100	-1.32737800	-1.26109200
H	-0.78325600	0.39959500	-1.16109200
C	-2.33514500	-0.84926700	-0.32776300
H	-2.59334100	-1.90676800	-0.25416900
C	-3.31161900	0.04509700	-0.11284500
C	-4.72161200	-0.38957600	0.19162800
H	-4.81765800	-1.47870200	0.20062600
H	-5.04358500	-0.00461200	1.16703000
H	-5.41947300	0.01384300	-0.55245900
C	-3.11128100	1.53775400	-0.14042300
H	-3.75937900	1.99698300	-0.89695800
H	-3.39977600	1.97139900	0.82495400
H	-2.08008500	1.83069400	-0.34763400

O1a

M06-2X/6-31+g(d,p),

el. energy = -466.723279 a.u.

C	4.09648400	0.34181600	-0.26466900
C	3.20594400	1.39344000	-0.04398700
C	1.87436200	1.12700200	0.27486500
C	1.40981600	-0.18957700	0.37977100
C	2.31240400	-1.23558600	0.15472000

References

(1) Cala, L.; Rubio-Presa, R.; García-Pedrero, O.; Fañanás, F. J.; Rodríguez, F., Synthesis of Spirocyclic Compounds by a Ring-Expansion/Cationic Cyclization Cascade Reaction of Chlorosulfate Derivatives, *Org. Lett.*, **2020**, *22*, 3846-3849.

(2) Natori, Y.; Sakuma, T.; Yoshimura, Y.; Kinami, K.; Hirokami, Y., Arabinoiminofuranoses, a New Class of a -Glucosidase Inhibitors, *Bioorg. Med. Chem. Lett.*, **2014**, *24*, 3298-3301.

(3) Cheung, F. K.; Lin, C.; Minissi, F.; Crivillé, A. L.; Graham, M. A.; Fox, D. J.; Wills, M., An investigation into the tether length and substitution pattern of arene-substituted complexes for asymmetric transfer hydrogenation of ketones, *Org. Lett.*, **2007**, *9*, 4659-4662.

(4) Herold, S.; Bafaluy, D.; Muñiz, K., Anodic Benzylic C(sp^3)–H Amination: Unified Access to Pyrrolidines and Piperidines, *Green Chem.*, **2018**, *20*, 3191-3196.

(5) Nieman, J. A.; Coleman, J. E.; Wallace, D. J.; Piers, E.; Lim, L. Y.; Roberge, M.; Andersen, R. J., Synthesis and antimitotic/cytotoxic activity of hemiasterlin analogues, *J. Nat. Prod.*, **2003**, *66*,183-199.

(6) Bartkovitz, D. J.; Brook, P.; Us, N. J.; Liu, J.; Ross, M., Substituted Pyrrolidine-2-Carboxamides, US 2010/0152190 Al, **2010**.

(7) Chen, W.; Walker, J. C. L.; Oestreich, M. Metal-Free Transfer Hydroiodination of C−C Multiple Bonds,*J. Am. Chem. Soc.*, **2019**, 141, *2*, 1135-1140.

(8) Hao, H. Y.; Mao, Y. J.; Xu, Z. Y.; Lou, S. J.; Xu, D. Q., Selective Cross-Dehydrogenative C(sp^3)-H Arylation with Arenes, *Org. Lett.*, **2020**, *22*, 2396-2402.

(9) Dabrowski, J. A.; Villaume, M. T.; Hoveyda, A. H., Enantioselective synthesis of quaternary carbon stereogenic centers through copper-catalyzed conjugate additions of aryl- and alkylaluminum reagents to acyclic trisubstituted enones, *Angew. Chem. Int. Ed Engl.*, **2013**, *52*, 8156-8159.

(10) Yu, X.; Wang, P.; Yan, D.; Lu, B.; Chen, J., Photocatalytic Neophyl Rearrangement and Reduction of Distal Carbon Radicals by Iminyl Radical-Mediated C-C Bond Cleavage, *Adv. Synth. Catal.,* **2018**, *360*, 3601-3606.

(11) Jud, W.; Kappe, C. O.; Cantillo, D., Development and Assembly of a Flow Cell for Single-Pass Continuous Electroorganic Synthesis Using Laser-Cut Components, *Chem.: Methods*, **2021**, *1*, 36-41.

(12) Plamondon, S. J.; Gleason, J. L., Total Synthesis of (-)-3-Oxoisotaxodione, *Org. Lett.*, **2022**, *24*, 2305-2309.

(13) Yilmaz, S.; Göksu, S., First Synthesis of Dopamine and Rotigotin Analogue 2-Amino-6,8-dimethoxy-1,2,3,4-tetrahydronaphthalene, *Synthetic Communications*, **2014**, *44*, 1058-1065.

(14) a) Grimme, S.; Neese, F., Fully Automated Quantum-Chemistry-Based Computation of Spin–Spin-Coupled Nuclear Magnetic Resonance Spectra, *Angew. Chem. Int. Ed.*, **2017**, *56*, 14763-14769; b) Bannwarth, C.; Ehlert, S.; Grimme, S., GFN2-xTB–An Accurate and Broadly Parametrized Self-Consistent Tight-Binding Quantum Chemical Method with Multipole Electrostatics and Density-Dependent Dispersion Contributions, *J. Chem. Theory Comput.*, **2019**, *15*, 1652-1671; c) Pracht, P.; Bohle, F.; Grimme, S., Automated exploration of the low-energy chemical space with fast quantum chemical methods, *Phys. Chem. Chem. Phys.*, **2020**, *22*, 7169-7192.

(15) Frisch, M.J.; Trucks, G- W.; Schlegel, H. B.; Scuseria, G. E.; Robb, M. A.; Cheeseman, J. R.; Scalmani, G.; Barone, V.; Petersson, G. A.; Nakatsuji, H.; Li, X.; Caricato, M.; Marenich, A. V.; Bloino, J.; Janesko, B. G.; Gomperts, R.; Mennucci, B.; Hratchian, H. P.; Ortiz, J. V.; Izmaylov, A. F.; Sonnenberg, J. L.; Williams-Young, D.; Ding, F.; Lipparini, F.; Egidi, F.; Goings, J.; Peng, B.; Petrone, A.; Henderson, T.; Ranasinghe, D.; Zakrzewski, V. G.; Gao, J.; Rega, N.; Zheng, G.; Liang, W.; Hada, M.; Ehara, M.; Toyota, K.; Fukuda, R.; Hasegawa, J.; Ishida, M.; Nakajima, T.; Honda, Y.; Kitao, O.; Nakai, H.; Vreven, T.; Throssell, K.; Montgomery, Jr. J. A.; Peralta, J. E.; Ogliaro, F.; Bearpark, M. J.; Heyd, J. J.; Brothers, E. N.; Kudin, K. N.; Staroverov, V. N.; Keith, T. A.; Kobayashi, R.; Normand, J.; Raghavachari, K.; Rendell, A. P.; Burant, J. C.; Iyengar, S. S.; Tomasi, J.; Cossi, M.; Millam, J. M.; Klene, M.; Adamo, C.; Cammi, R.; Ochterski, J. W.; Martin, R. L.; Morokuma, K.; Farkas, O.; Foresman, J. B.; Fox, D. J. Gaussian16, Revision C.01; Gaussian, Inc., Wallingford, CT, **2019**.

(16) a) Becke, A. D., Density-functional thermochemistry. III. The role of exact exchange, *J. Chem. Phys.*, **1993**, *98*, 5648-5652; b) Lee, C.; Yang, W.; Parr, R. G., Development of the Colle-Salvetti correlation-energy formula into a functional of the electron density, *Phys. Rev. B*, **1988**, *37*, 785-789.

(17) a) Ditchfield, R.; Hehre, W. J.; Pople, J. A., Self-Consistent Molecular-Orbital Methods. IX. An Extended Gaussian-Type Basis for Molecular-Orbital Studies of Organic Molecule, *J. Chem. Phys.*, **1971**, *54*, 724-728; b) Hariharan, P. C.; Pople, J. A., The influence of polarization functions on molecular orbital hydrogenation energies, *Theor. Chim. Acta*, **1973**, *28*, 213-222; c) Hehre, W. J.; Ditchfield, R.; Pople, J. A., Self-Consistent Molecular Orbital Methods. XII. Further Extensions of Gaussian-Type Basis Sets for Use in Molecular Orbital Studies of Organic Molecules, *J. Chem. Phys.*, **1972**, *56*, 2257-2261.

(18) Marenich, A. V.; Cramer, C. J.; Truhlar, D. G., Universal Solvation Model Based on Solute Electron Density and on a Continuum Model of the Solvent Defined by the Bulk Dielectric Constant and Atomic Surface Tensions, *J. Phys. Chem. B*, **2009**, *113*, 6378-6396.

(19) Zhao, Y.; Truhlar, D. G., The M06 suite of density functionals for main group thermochemistry, thermochemical kinetics, noncovalent interactions, excited states, and transition elements: two new functionals and systematic testing of four M06-class functionals and 12 other functionals, *Theor. Chem. Acc.*, **2008**, *120*, 215-241.

(20) Clark, T.; Chandrasekhar, J.; Spitznagel, G. W.; Schleyer, P. V. R., Efficient diffuse function-augmented basis sets for anion calculations. III. The 3-21+G basis set for first-row elements, Li-F, *J. Comput. Chem*., **1983**, *4*, 294-301.

(21) a) Gonzalez, C.; Schlegel, H. B., Reaction path following in mass-weighted internal coordinates, *J. Chem. Phys*., **1990**, *94*, 5523-5527; b) Gonzalez, C.; Schlegel, H. B., An improved algorithm for reaction path following, *J. Chem. Phys*., **1989**, *90*, 2154-2161.

(22) Chai, J.-D.; Head-Gordon, M., Long-range corrected hybrid density functionals with damped atom–atom dispersion corrections, *Phys. Chem. Chem. Phys.*, **2008**, *10*, 6615-6620.

(23) a) Weigend, F.; Ahlrichs, R., Balanced basis sets of split valence, triple zeta valence and quadruple zeta valence quality for H to Rn: Design and assessment of accuracy, *Phys. Chem. Chem. Phys.*, **2005**, *7*, 3297-3305; b) Weigend, F., Accurate Coulomb-fitting basis sets for H to Rn, *Phys. Chem. Chem. Phys.*, **2006**, *8*, 1057-1065.

(24) Yao, Y.-X.; Zhang, H.-W.; Lu, C.-B.; Shang, H.-Y.; Tian, Y.-Y., Highly Selective and Practical Iron-Catalyzed Formal Hydrogenation of Epoxides to Primary Alcohols Using Formic Acid, *Eur. J. Org. Chem*., **2023**, *26*, e2023001.
